# Non-Covalent Interactions Mimic the Covalent: An Electrode-Orthogonal Self-Assembled Layer

**DOI:** 10.1021/jacs.3c04387

**Published:** 2023-08-07

**Authors:** Deepak Badgurjar, Madison Huynh, Benjamin Masters, Anna Wuttig

**Affiliations:** Department of Chemistry, University of Chicago, Chicago, Illinois 60637, United States

## Abstract

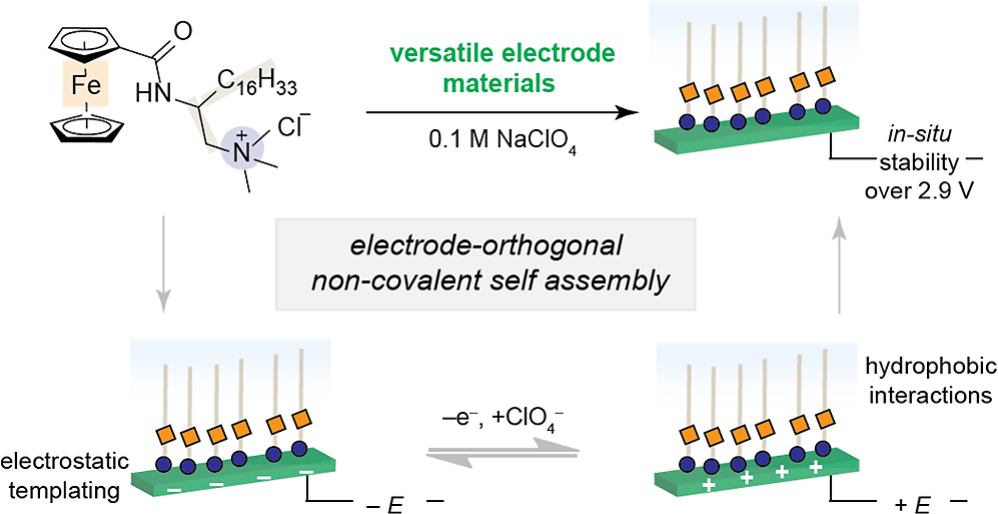

Charge-transfer events central to energy conversion and storage and molecular sensing occur at electrified interfaces. Synthetic control over the interface is traditionally accessed through electrode-specific covalent tethering of molecules. Covalent linkages inherently limit the scope and the potential stability window of molecularly tunable electrodes. Here, we report a synthetic strategy that is agnostic to the electrode’s surface chemistry to molecularly define electrified interfaces. We append ferrocene redox reporters to amphiphiles, utilizing non-covalent electrostatic and van der Waals interactions to prepare a self-assembled layer stable over a 2.9 V range. The layer’s voltammetric response and *in situ* infrared spectra mimic those reported for analogous covalently bound ferrocene. This design is electrode-orthogonal; layer self-assembly is reversible and independent of the underlying electrode material’s surface chemistry. We demonstrate that the design can be utilized across a wide range of electrode material classes (transition metal, carbon, carbon composites) and morphologies (nanostructured, planar). Merging atomically precise organic synthesis of amphiphiles with *in situ* non-covalent self-assembly at polarized electrodes, our work sets the stage for predictive and non-fouling synthetic control over electrified interfaces.

## Introduction

**Scheme 1:**
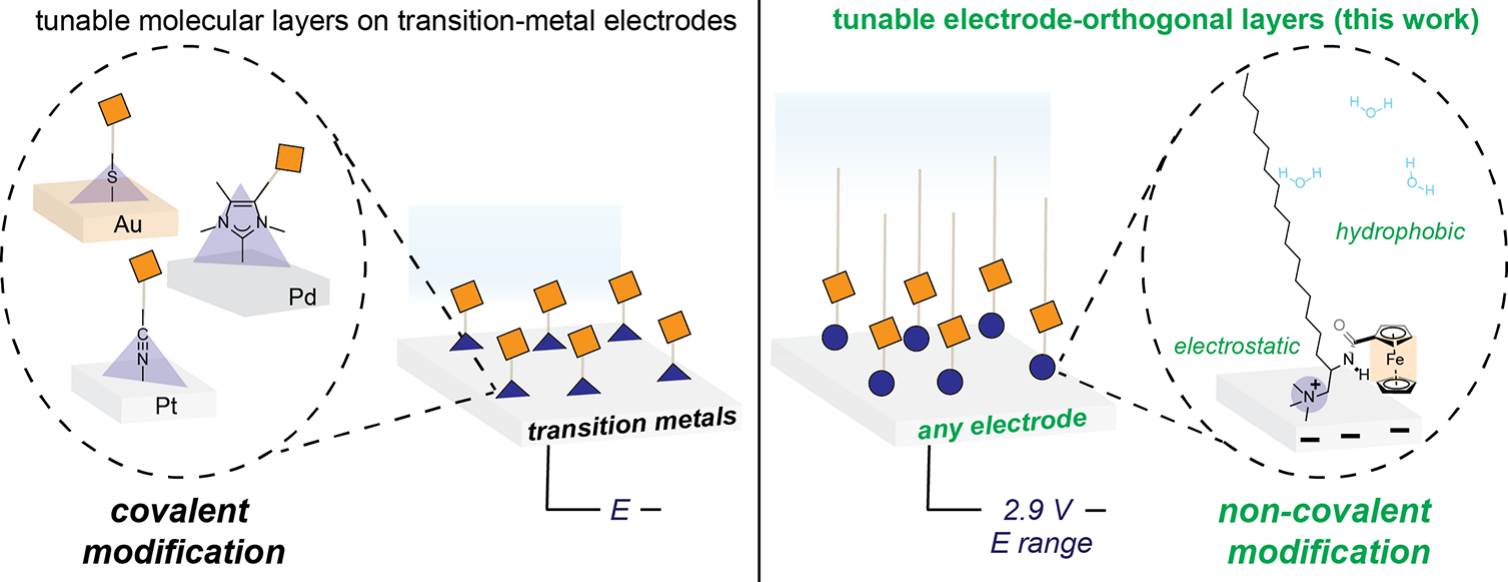
Predominant Synthetic Strategy to Produce Molecularly Tunable Layers on Electrodes Utilize Covalent Interactions to Modify Transition-Metal Surfaces (Left) This work describes an alternative, non-covalent approach for modification of a diverse array of electrode materials, yielding electrochemical charge-transfer properties that mimic those of covalently modified systems over an expanded 2.9 V range. The electrode-orthogonal layer (right) is easily removed by rinsing with water.

Covalent bond formation between molecules and electrode surfaces serves as a predominant synthetic strategy to molecularly define the structure of electrified interfaces. For example, by leveraging the robust metal-sulfur bond,^1−3^ redox-active or field-sensitive reporters containing a thiol precursor can be installed at an electrode surface (Scheme 1, left). This synthetic approach has been used to answer key mechanistic questions in energy conversion and storage, biomolecular sensing, and electronic devices, such as the electron transfer properties of biomolecules,^4,5^ interfacial acid–base equilibria,^6−8^ and the directionality of electron transfer reactions,^9,10^ as well as enabling new modalities to enhance electrochemical reactivity,^11−13^ among numerous other advances. However, the reliance of specific covalent bond-forming events (*e.g.*, using thiol, isocyanide, and carbene precursors)^14−17^ on select metal surfaces (Au, Pd, Pt, Cu, Ag, and Hg) precludes generalizability across: (1) a wider range of electrode materials and (2) applied potentials due to competitive oxidative and reductive desorption.^18−22^ While the use of π–π stacking of pyrene with carbon electrodes or diazonium grafting onto electrode surfaces has enabled systematic and tunable modifications,^23−29^ these linkages are specific to their underlying electrode material and exhibit competitive potential-dependent desorption, electropolymerization, or multilayer formation.^30−33^ The identification of new material compositions for electrochemical applications, namely, non-precious metal and metal-free electrodes, necessitates a general synthetic technology that is independent of the potential-dependent linkage stability *in situ*. Such a strategy would enable us to molecularly define the structure of the electrified interface without irreversibly modifying the electrode itself; this electrode-orthogonal approach would be agnostic to the surface chemistry of the electrode material yet retain the modular and predictive nature of covalent synthetic strategies.

The addition of amphiphiles to electrochemical systems is known to modulate key charge-transfer events.^30,34−40^ It is theorized that these charge-transfer events are driven by the non-covalent electrostatic attraction of the charged amphiphile to the oppositely charged electrode surface, which creates a hydrophobic pocket.^41−44^ This self-assembly has been posited to be potential-dependent,^41,43−45^*e.g.*, for a cationic amphiphile, the structure is formed negative of the potential of zero free charge (PZFC, the potential at which the interfacial field is the weakest). Yet, this mechanistic model cannot explain prior work that suggests that cationic amphiphiles are still positioned at the polarized interface at potential values where electrostatic repulsion should repel them (i.e., applied potential values are far positive of the PZFC).^42^ Thus, we hypothesized that non-covalent van der Waals interactions between aliphatic chains of the amphiphile could override electrostatic repulsion.

Here, we reveal that the combination of non-covalent electrostatic and van der Waals interactions enables the formation of a self-assembling molecular layer. We report that the latter non-covalent interaction enables the layer to remain intact even at applied potential values where electrostatic repulsion should repel the layer. We append a ferrocene (Fc) redox reporter onto a series of cationic amphiphiles with varying aliphatic chains to enable us to track and quantify layer formation. We leverage this insight in a synthetic strategy to produce self-assembled electrode-orthogonal layers at electrode interfaces that are stable over an expanded potential range (Scheme 1, right). As the layer is formed solely due to non-covalent interactions, self-assembly occurs on a wide array of electrode material compositions without permanent surface structural changes, i.e., the layer is removed by rinsing the electrode with water and can be restored by reintroduction of the amphiphile to the electrolyte. The *in situ* self-assembled layer is maintained in electrolytes that contain the amphiphile over potential ranges from −1.8 to 1.1 V vs Ag/AgCl, a window 0.9 V wider than the potential stability range of covalent linkers^18−22^ and 0.3 V greater than existing non-covalent^23,29^ linkers.

## Results and Discussion

### Structure-Dependent Ferrocene Redox Features Observed in the Absence of Covalent Tethering

**Scheme 2:**
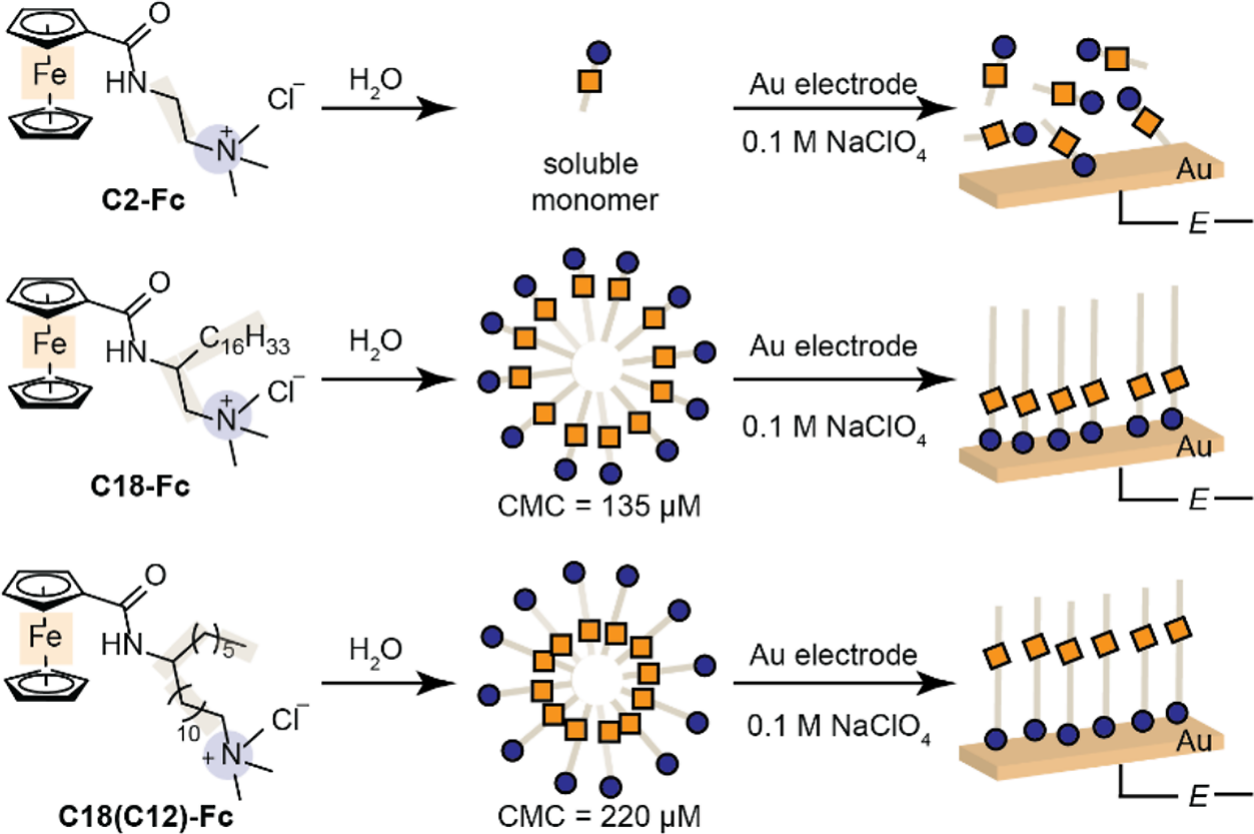
Summary of **C2-Fc**, **C18-Fc**, and **C18(C12)-Fc** Compounds Synthesized and Supramolecular Aggregates Thereof Micelles formed in the bulk aqueous solution (middle). Charge-balancing anions omitted for clarity. Simplified schematic of proposed interfacial structure upon contact with charged polycrystalline Au working electrode in aqueous electrolytes (right). Water, charge-balancing anions, and possible structural disorder of aliphatic chains omitted for clarity.

To track and quantify layer formation, we appended a ferrocene (Fc) redox reporter to cationic amphiphiles. Scheme 2 summarizes the synthesized probes (see Supporting Information (SI), for details): (1) **C2-Fc**, a control monomer without a long aliphatic chain; (2) **C18-Fc**, a monomer containing a long aliphatic chain with the Fc moiety close to the ammonium; and (3) **C18(C12)-Fc**, a monomer containing an identical aliphatic chain but with the Fc moiety positioned further away from the cationic group. We determined each monomer’s critical micelle concentration (CMC) and surface tension (Scheme 2, middle, Table S1, Table S2, Figures S1 and S2). These results demonstrate that the hydrophobic interactions induced by the presence of the long alkyl chains serve as the primary non-covalent interaction to drive supramolecular aggregate formation in aqueous solution at μM quantities, Scheme 2, middle.

**Figure 1:**
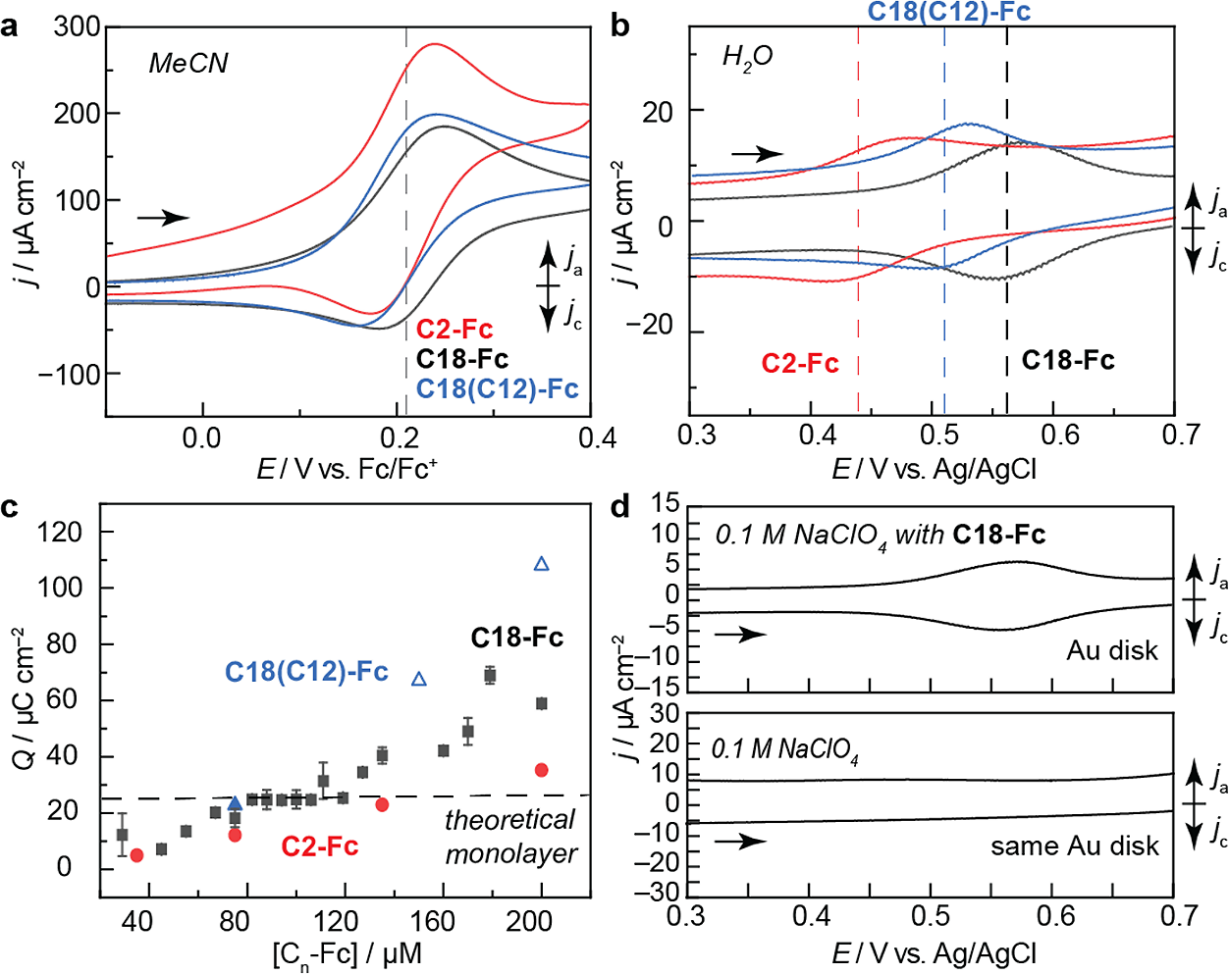
Electrochemical response of **C2-Fc**, **C18-Fc**, and **C18(C12)-Fc** in varying electrolyte media. (a) CV of 1 mM **C2-Fc** (red), 1 mM **C18(C12)-Fc** (blue), or 1 mM **C18-Fc** (black) in 0.1 M TBAClO_4_ in MeCN collected at 50 mV s^–1^ on a Au working disk electrode. (b) CV of 75 μM **C2-Fc** (red), 75 μM **C18(C12)-Fc** (blue), or 75 μM **C18-Fc** (black) in 0.1 M NaClO_4_ in H_2_O collected at 50 mV s^–1^ on a Au working disk electrode. Dashed lines in panels **a** and **b** estimate the *E*_1/2_ of the reversible CV waves observed. (c) Charge integration of the Fc anodic redox in 0.1 M NaClO_4_ for data collected on a Au disk electrode for **C18-Fc** (black squares, error bars represent the average and range of 2–3 independent runs), **C18(C12)-Fc** (blue diamonds, where open blue diamond data points are convoluted with **C18(C12)-Fc** precipitation), and **C2-Fc** (red circles). The calculated theoretical charge for a monolayer of **C18-Fc** is indicated by the black dashed line. (d) Rinse test of **C18-Fc** in an aqueous electrolyte. (**Top**) CV of 200 μM **C18-Fc** in 0.1 M NaClO_4_ in H_2_O collected at 20 mV s^–1^ on a Au disk working electrode. (**Bottom**) CV of the same Au working electrode as the (**top**) in 0.1 M NaClO_4_ in H_2_O that immediately follows rinsing of the Au electrode in H_2_O. CV collected at 20 mV s^–1^. All CVs collected with a positive direction of scan under N_2_ atmosphere.

The redox features of **C2-Fc**, **C18-Fc**, and **C18(C12)-Fc** are nearly identical in nonaqueous media and indicative of diffusion-controlled and reversible single-electron transfer. We chose Au disk electrodes to enable interfacial structural characterization via surface-enhanced infrared absorption spectroscopy (SEIRAS), see below. Figure 1a depicts the cyclic voltammogram (CV) of **C2-Fc** (red), **C18-Fc** (black), and **C18(C12)-Fc** (blue) in acetonitrile (MeCN) containing 0.1 M tetrabutylammonium perchlorate (TBAClO_4_). We chose ClO_4_^–^ because studies show that the reversibility of the Fc redox wave is dependent on the electrolyte anion and is most preserved in the presence of ClO_4_^–^.^46−148,51^ For all molecules examined, near-identical redox features centered at an anodic peak potential value, *E*_pa_, of 232–245 mV vs Fc/Fc^+^ and a cathodic peak potential, *E*_pc_, value of 170–185 mV vs Fc/Fc^+^ are observed. Following, the redox potentials (*E*_1/2_) lie at 210 ± 5 mV vs Fc/Fc^+^ (gray, Figure 1a). The peak potential separation between *E*_pa_ and *E*_pc_ is 60 mV, indicative of diffusion-controlled and reversible single-electron transfer.^48^ Diffusion coefficients for **C2-Fc**, **C18-Fc**, and **C18(C12)-Fc** are similar (Figure S3). These results indicate that, in nonaqueous media, **C2-Fc**, **C18-Fc**, and **C18(C12)-Fc** exist in a monomeric, solution-dissolved form, where changes in the aliphatic structure minimally impact the structure at the electrified Au interface (key electrochemical data summarized in Table S3).

In contrast, the redox behaviors of the molecules are drastically different in aqueous electrolytes and mimic those previously reported for covalently bound ferrocene at varying distances from the electrode surface. Figure 1b shows the CV of **C2-Fc** (red), **C18-Fc** (black), and **C18(C12)-Fc** (blue) in 0.1 M NaClO_4_. For **C2-Fc**, we observe *E*_pa_ at 0.472 V vs Ag/AgCl (all aqueous reference potentials quoted vs Ag/AgCl with resistance correction, Figure S4) and *E*_pc_ at 0.418 V (*E*_1/2_ = 0.445 V, Figures 1b and S5), exhibiting a peak-to-peak separation of ∼60 mV (Δ*E*_p_ = *E*_pa_ – *E*_pc_) that remains constant at varying scan rates (Figure S6). These results are indicative of freely diffusive **C2-Fc** species in a monomeric form (Scheme 1, right). However, the *E*_1/2_ value for **C18(C12)-Fc** exhibits a ∼67 mV positive shift from the redox wave observed for **C2-Fc** (*E*_pa_ at 0.525 V and a *E*_pc_ at 0.499 V, *E*_1/2_ = 0.512 V, Figures 1b and S7). This reproducible shift (Figure S8 and Table S4) is in line with previous work on covalently bound ferrocenylalkane thiols to Au electrodes, separating the Fc by six methylene units or more away from the S–Au linkage.^46,47,50^ In these covalent systems, the positive Fc redox potential shifts are attributed to a potential drop of the Fc in the interfacial layer of low dielectric strength induced by the alkane matrix bound to Au.^46,47,49−51^ In addition, a small Δ*E*_p_ value of 26 mV is observed. This value is inconsistent with the 60-mV value expected for the single-electron redox signal associated with a diffusing species (or its micelle form),^52^ suggesting that **C18(C12)-Fc** organizes to form an immobilized structure at the electrode surface that positions the Fc units within an aliphatic matrix. This hypothesis is supported by the linearity expected and observed between the anodic (*j*_pa_) and cathodic (*j*_pc_) peak current with the CV scan rate, υ, (Figure S9) for surface-adsorbed redox-active species.^53^ For **C18-Fc**, we observe *E*_pa_ at 0.566 V and *E*_pc_ at 0.559 V (*E*_1/2_ = 0.563 V), exhibiting a small Δ*E*_p_ value of 7 mV, and a 51 mV shift more positive from the redox wave observed for **C18(C12)-Fc** (Figure 1b, black, Figures S10 and S11). The small Δ*E*_p_ value (7 mV) observed for **C18-Fc** together with the linearity observed for *j*_pa_ and *j*_pc_ with υ (Figure S9) is consistent with those reported for Fc covalently bound to Au electrode surfaces via thiol linkers separating the Fc by one or two methylene units from the S–Au linkage.^54,57^ As previous work demonstrates that the positive *E*_1/2_ redox shifts of covalently bound ferrocenylalkane thiols track the position of Fc placed inside the bound alkane layer with low dielectric strength,^46,47,49,50^ the increase in *E*_1/2_ for **C18-Fc** relative to **C18(C12)-Fc** further demonstrates that the Fc tethered to the long alkyl chain is in regions of lower dielectric strength than **C18(C12)-Fc** (key electrochemical data summarized in Table S5). The decrease in the normalized double layer capacitance (Figure S5) from data collected in **C2-Fc**, Figure 1b (red) to **C18(C12)-Fc** (blue) and **C18-Fc** (black) further supports the formation of a unique structure around the electrified Au with lower dielectric strength.^59,60^ Together, these electrochemical features in aqueous media are consistent with a structural model where the presence of a long aliphatic chain leads to the formation of immobilized structures with the ammonium head group pointing toward the Au surface, Scheme 2, right, on the timescale of the electrochemical measurement.

Charge integration of the Fc redox wave is consistent with monolayer formation. Ferrocene-terminated thiol monolayers on Au have been estimated to be correlated with a charge integration of ∼43 μC cm^–2^.^51,61−65^ This theoretical value is determined by taking the radius of ferrocene^66^ and assuming a densely packed monolayer. The hypothetical charge integration for the **C18-Fc** monolayer is ∼24 μC cm^–2^ (see SI, Table S6 and Figure S12). Indeed, we observe that the charge integration for the Fc redox wave plateaus at ∼24 μC cm^–2^ for **C18-Fc** at Au disk electrodes for CV experiments conducted in the presence of **C18-Fc** between 67 and 119 μM (Figure 1c, black). At higher concentrations of **C18-Fc** in solution, the charge integration increases, suggesting multilayer formation. This charge integration is different from that observed for freely diffusing **C2-Fc**, which rises linearly with increasing bulk solution concentration (Figure 1c, red) and consistently exhibits lower integrated values. We note that the investigation of a similar charge integration relationship for **C18(C12)-Fc** is convoluted by solubility limitations in the presence of 0.1 M NaClO_4_ (Figure 1c, blue). Therefore, we cannot conclude that monolayer formation occurs on **C18(C12)-Fc**. Together, these results suggest that **C18-Fc** forms a monolayer at the electrified Au interface.

The data are consistent with non-covalent binding of **C18-Fc** to Au electrode surfaces. Figure 1d (top) demonstrates the redox feature for **C18-Fc** observed on a freshly annealed Au electrode surface. Subsequent scans of the Au electrode in the presence of **C18-Fc** alter neither the position nor the shape of the CV feature (Figure S13), demonstrating that the immobilized structure formed is stable over the timescale of the experiment. Following, the Au electrode was rinsed in water and then introduced to an electrolyte solution that does not contain **C18-Fc**. The resultant CV, Figure 1d (bottom), shows a featureless CV, demonstrating that **C18-Fc** is not covalently bound to the Au surface and that the layer can be removed by rinsing the electrode with water. The reintroduction of **C18-Fc** to this new electrolyte solution restores the redox feature (Figure S14). These results highlight that layer formation and maintenance involves the presence of a micromolar concentration of **C18-Fc** in the bulk solution lower than or near our measured CMC values (see Table S1). Indeed, our observation is consistent with literature reports on commercially available surfactants, where surface aggregate formation (hemimicelles) at a non-electrified solid/liquid interface occurs at surfactant bulk concentration values less than or near the reported CMC values.^67−69^ Together, our data point to a self-assembly mechanism of the **C18-Fc** layer at the electrified Au interface that occurs in the absence of covalent bond formation and in the presence of **C18-Fc** in the bulk solution at micromolar concentrations, Scheme 2, right.

### Spectroelectrochemical Data Consistent with Self-Assembled Structure

**Figure 2:**
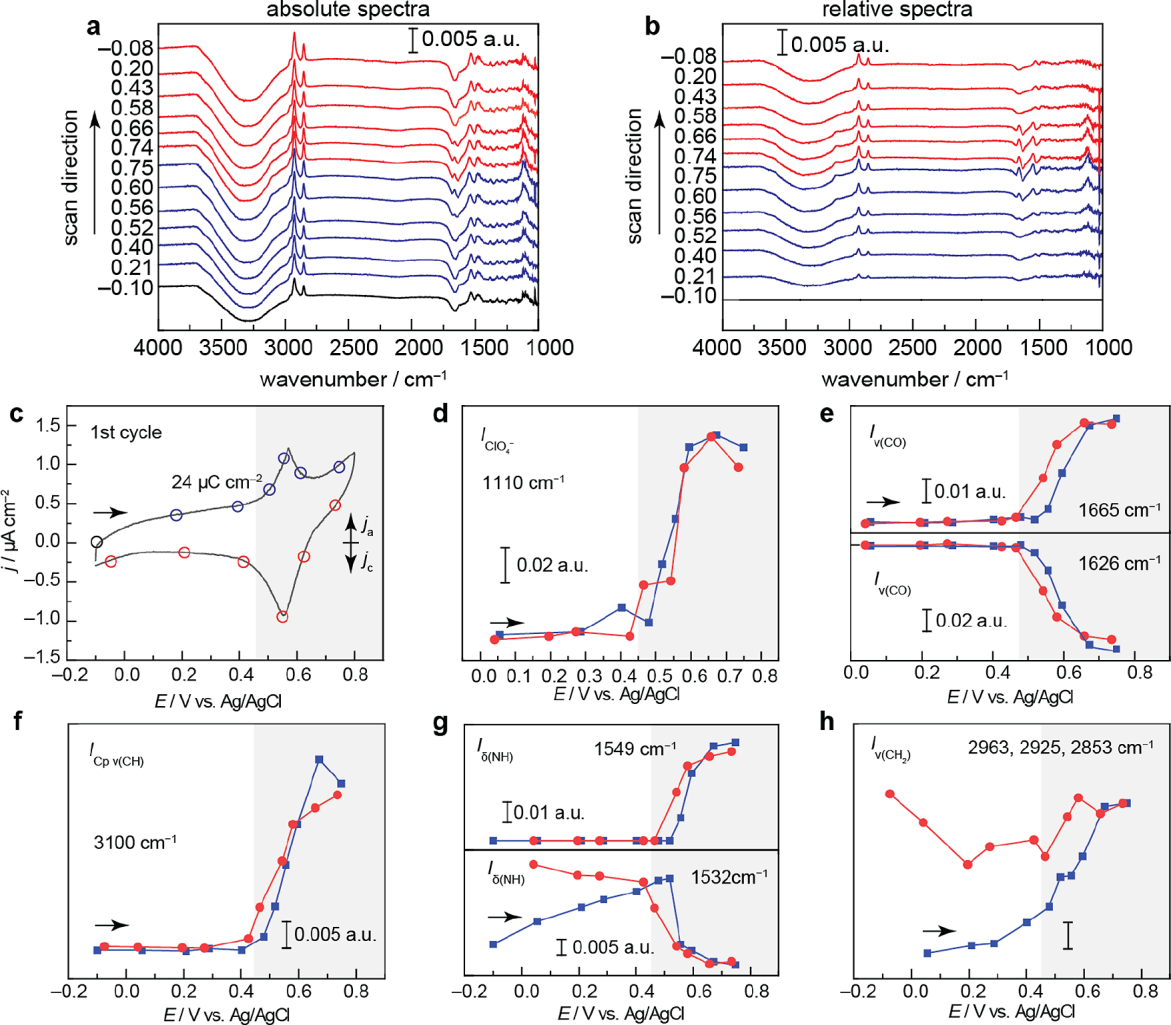
Spectroscopic data of self-assembled layer. (a) SEIRA spectra collected on the first CV cycle at the potential values indicated, where the background spectrum was collected at −0.1 V vs Ag/AgCl in 0.1 M NaClO_4_ in the absence of 200 μM **C18-Fc**. (b) Identical spectra as shown in as **a**, however, the background spectrum was collected at −0.1 V vs Ag/AgCl in 0.1 M NaClO_4_ in the presence of 200 μM **C18-Fc**. (c) The initial, first CV cycle collected upon the addition of 200 μM **C18-Fc** in tandem with **a** and **b** at 2 mV s^–1^ from −0.1 V vs Ag/AgCl. The anodic integrated charge is shown. (d) Integrated band intensity (IBI) taken from **b** of the peak centered at 1110 cm^–1^. (e) IBI taken from **b** of the peak centered at 1626 cm^–1^. (f) IBI taken from **b** of the peak centered at 3100 cm^–1^. (g) IBI taken from **b** of the peak centered at 1532 cm^–1^. (h) IBI taken from **b** of the peak centered at 2963, 2925, and 2853 cm^–1^.

SEIRA spectra are consistent with the formation of a self-assembled **C18-Fc** layer at Au. SEIRAS utilizes nanostructured electrode surfaces to enhance IR absorption of molecules with transition dipole moments perpendicular to the surface.^70,71^Figure 2c depicts the CV of **C18-Fc** collected using a SEIRAS-active Au film (electrochemically active surface area characterized and compared to the Au disk in Figure S15).^72,73^ We observe a near-identical redox feature to that observed on the Au disk electrode, see above (*E*_1/2_ at 0.562 V). We calculate an integrated charge (24 μC cm^–2^) value identical to that estimated for monolayer coverage. These results indicate that, despite the difference in the preparation of Au films required for SEIRAS studies (nanostructured) and the higher **C18-Fc** solution concentration utilized for the spectroscopic measurements (200 μM), the current-voltage profile and the surface population of **C18-Fc** are identical to that observed on Au disks.

**Scheme 3:**
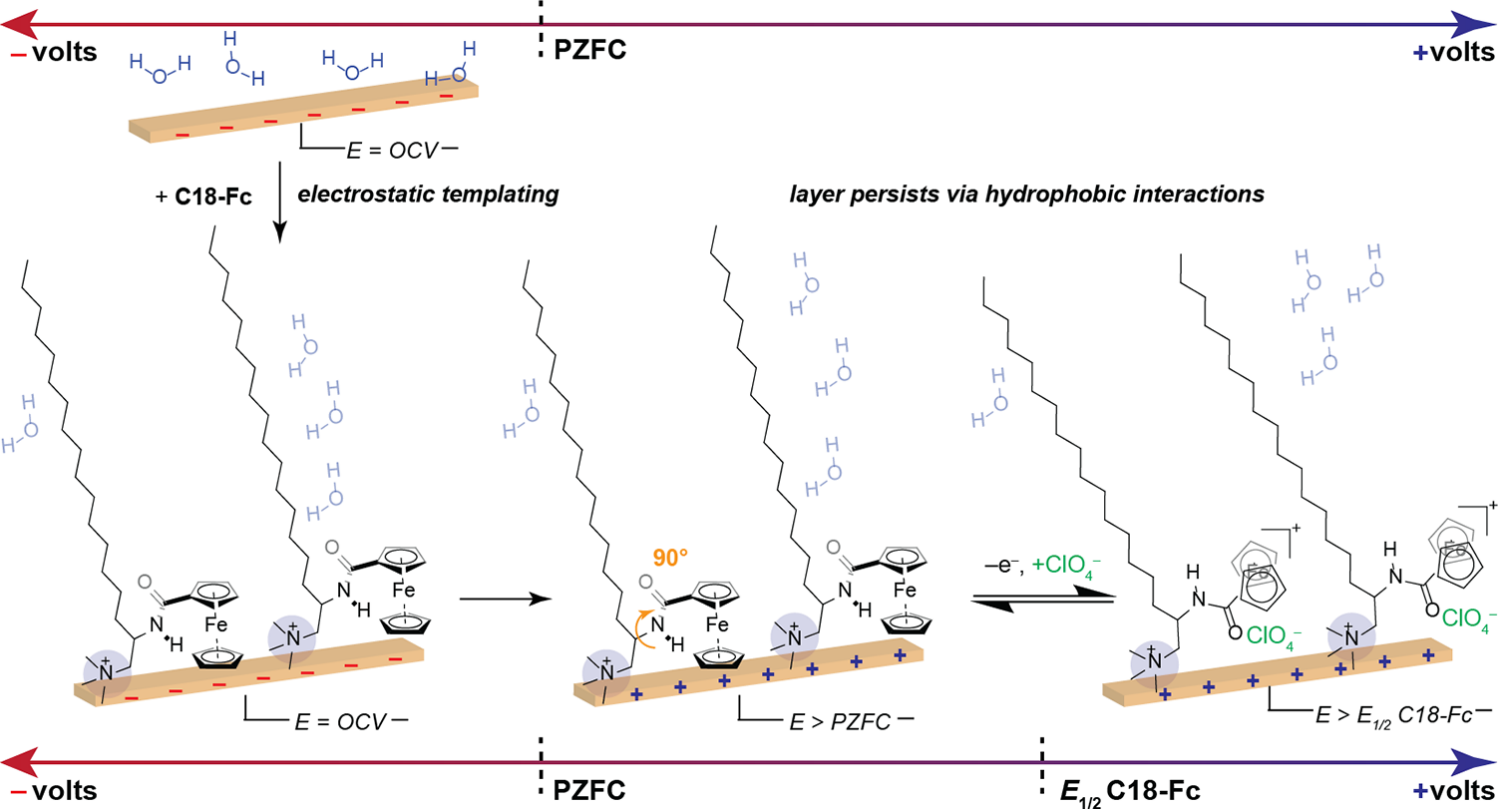
Proposed Asembly and Structure of Self-Assembled Layer Driven by Non-Covalent Interactions At the open circuit voltage (OCV), which is negative of the potential of zero free charge (PZFC) of polycrystalline Au, the addition of **C18-Fc** results in the formation of a layer due to electrostatic attraction between the cationic group and the negatively polarized electrode. Upon the application of oxidative potential (*E* > Fc/Fc^+^), the oxidized **C18-Fc** ion pairs with the perchlorate anion, resulting in a rotation around the C(sp^3^)–N(sp^3^) bond and a tilting of the aliphatic unit toward the interface. After the initial application of potential, the layer persists for multiple CV cycles at oxidizing potentials due to favorable hydrophobic interactions between the alkyl chains.

Figure 2a depicts the SEIRA spectra collected during the CV scan in Figure 2c. The background spectrum was collected at −0.10 V (the open circuit voltage, OCV) in the absence of **C18-Fc**. Upon the addition of **C18-Fc**, significant spectroscopic changes are observed (Figure 2a, black, −0.10 V). We observe a bleach at 3308 and 1668 cm^–1^, attributed to the ν(OH) stretching and δ(HOH) bending modes of interfacial water.^62,74^ These results demonstrate that the addition of **C18-Fc** to the electrolyte expels interfacial water. We observe a rise at 2963, 2925, and 2853 cm^–1^, attributed to the ν_sym_ (N-CH_3_) of the ammonium group, ν_as_ (CH_2_) and ν_sym_ (CH_2_) of the aliphatic tail in an all-*trans* configuration, respectively.^75−79^ These ν_as_ (CH_2_) and ν_sym_ (CH_2_) values are in line with reported values for covalently bound alkanethiol monolayers on Au in the absence of electrolyte^76,78^ as well as those reported *in situ* for Fc-terminated alkane thiol monolayers.^51,80^ We observe a rise at 1625 cm^–1^, which does not shift in D_2_O, Figure S16. The peak position is in line with the carbonyl stretching frequency for amides,^81,82^ and thus, we assign the band to the ν(CO) of the amide linkage. A rise at 1535 cm^–1^ is observed, which significantly shifts in D_2_O (1535 to 1422 cm^–1^, Figure S16). Together with the computational estimation for the δ(NH) in **C18-Fc** (Figure S17, 1547 cm^–1^), we assign this feature to the δ(NH) of the amide. We observe a rise at 1479 cm^–1^, which is present in D_2_O (Figure S16). This value is in line with the literature-reported values for both δ(CH_2_) of the aliphatic tail and the ν(Fe–C) stretching mode of the Fc.^62,77,80,83^ We observe a rise at 1110 cm^–1^. This peak is assigned to the perchlorate anion.^62^ Taken together, our absolute spectroscopic observations show that **C18-Fc** forms a molecular layer, repelling interfacial water with a significant population of the ammonium head group pointing towards the surface and the Fc oriented with the Fe–C axis primarily perpendicular to the surface, Scheme 3 (left, bottom). We note, however, that our data do not rule out structural disorder in the aliphatic tail region, *e.g.*, the tails can be tilted in various directions to produce domains of dense and sparse aliphatic grouping. As the OCV of the system in the absence of **C18-Fc** is lower than the potential of zero free charge of Au (−0.011 to 0.336 V,^84−86^ range quoted for predominant low index facets of Au present in SEIRAS-active films^73^), we rationalize that the non-covalent electrostatic interactions between the negatively charged Au electrode and the positively charged ammonium cations electrostatically template the layer assembly on the timescale of the CV experiment. Similarly, the presence of a perchlorate signal upon **C18-Fc** addition at the OCV suggests that the counterion is not fully replaced by the negatively charged interface, further supporting structural disorder in the layer.

The potential-dependent changes to the **C18-Fc** spectroscopic features demonstrate that the layer remains intact during Fc redox, mimicking features reported for the analogous covalently bound ferrocene. To accentuate the differences in the spectroscopic changes as a function of the applied potential, the relative spectra are reported in Figure 2b. We observe that as Fc is oxidized, Figure 2c, the feature assigned to ClO_4_^–^ increases, Figure 2d, blue. This observation suggests that ClO_4_^–^ is sequestered from the bulk solution to ion pair with the oxidized **C18-Fc**, similar to previous observations for Fc covalently bound to an Au surface.^62,65,87^ The peak assigned to the ν(CO) decreases in favor of a new feature centered at 1665 cm^–1^ (Figures 2b,e, blue). We hypothesize that the blueshift is due to the oxidation of the Fc, as we would expect the conjugated amide CO bond to contract,^51^ and identical features are observed in D_2_O (Figures S16, S18, and S19). We observe a new feature at 3100 cm^–1^ (Figures 2b,f), coincident with Fc oxidation, Figure 2c. This feature is also observed in D_2_O (Figures S16, S18, and S19) and is in line with the wavenumber reported for ν(CH) of the Fc cyclopentadienyl ring.^51,62,80,83,88,89^ These results demonstrate that Fc oxidation induces a structural change that favors the cyclopentadienyl rings to orient perpendicular to the interface, Scheme 3, right. Interestingly, this structural change has also been invoked for ferrocene alkyl thiols *covalently* bound to Au upon oxidation.^62,77,80,83,87^ We observe a rise in the peak assigned to the δ(NH), Figure 2b,g, blue. As Fc is oxidized, this peak exchanges in favor of a peak centered at 1549 cm^–1^. We hypothesize that the observed blueshift from 1535 to 1549 cm^–1^ arises due to the shortening of the bonds conjugated to the cyclopentadienyl rings. We observe a rise in the features corresponding to the aliphatic tail and ammonium head group, Figure 2b,h. This combined signal intensity increases as Fc is oxidized but persists as the applied potential switches to the negative direction (Figure 2h, red). This observation suggests that as the Fc is oxidized, the alkyl tails tilt away from the surface normal, Scheme 3, right. We hypothesize that since the Fc is embedded inside the alkyl chain, the sequestration of ClO_4_^–^ as an ion-pairing partner from the bulk solution results in a global tilt of the alkyl chain as well as an orientation change of the Fc^+^ unit relative to the electrode surface normal to accommodate the anion. Together, these results demonstrate that upon Fc oxidation, the C–N of the amide twists to favor a high surface population of Fc^+^ parallel to the surface, accompanied by ion-pairing with ClO_4_^–^ and a global tilt of the **C18-Fc** moiety relative to the surface normal.

Comparison of the first scan for the self-assembled **C18-Fc** layer with the second subsequent scan shows the convergence of the self-assembled interfacial structure (see Figures S20 and S21). Importantly, SEIRAS experiments conducted in the presence of **C2-Fc** do not reveal any significant spectroscopic changes (Figure S22), showing that layer formation is not observed for the freely diffusing **C2-Fc** control. This result is consistent with a structural model in which the secondary hydrophobic interaction between neighboring **C18-Fc** serves to establish a layer of **C18-Fc** at the Au interface. Without these non-covalent interactions, co-localization does not occur. We note that our conclusions lie in contrast to those in the literature that suggest that the ammonium head group is electrostatically repelled from the electrochemical interface at positive potentials.^43,44,90^ Thus, we reason, after the initial templating via electrostatic interactions between the ammonium head group and the Au surface, the secondary hydrophobic interactions between **C18-Fc** monomers enable the layer to persist at potential values far more positive than the PZFC, Scheme 3, right, on the timescale of the CV experiment.

### Non-Covalent Design Enables Molecular Layer to Self-Assemble at a Variety of Materials, i.e., via an Electrode-Orthogonal Process

**Figure 3:**
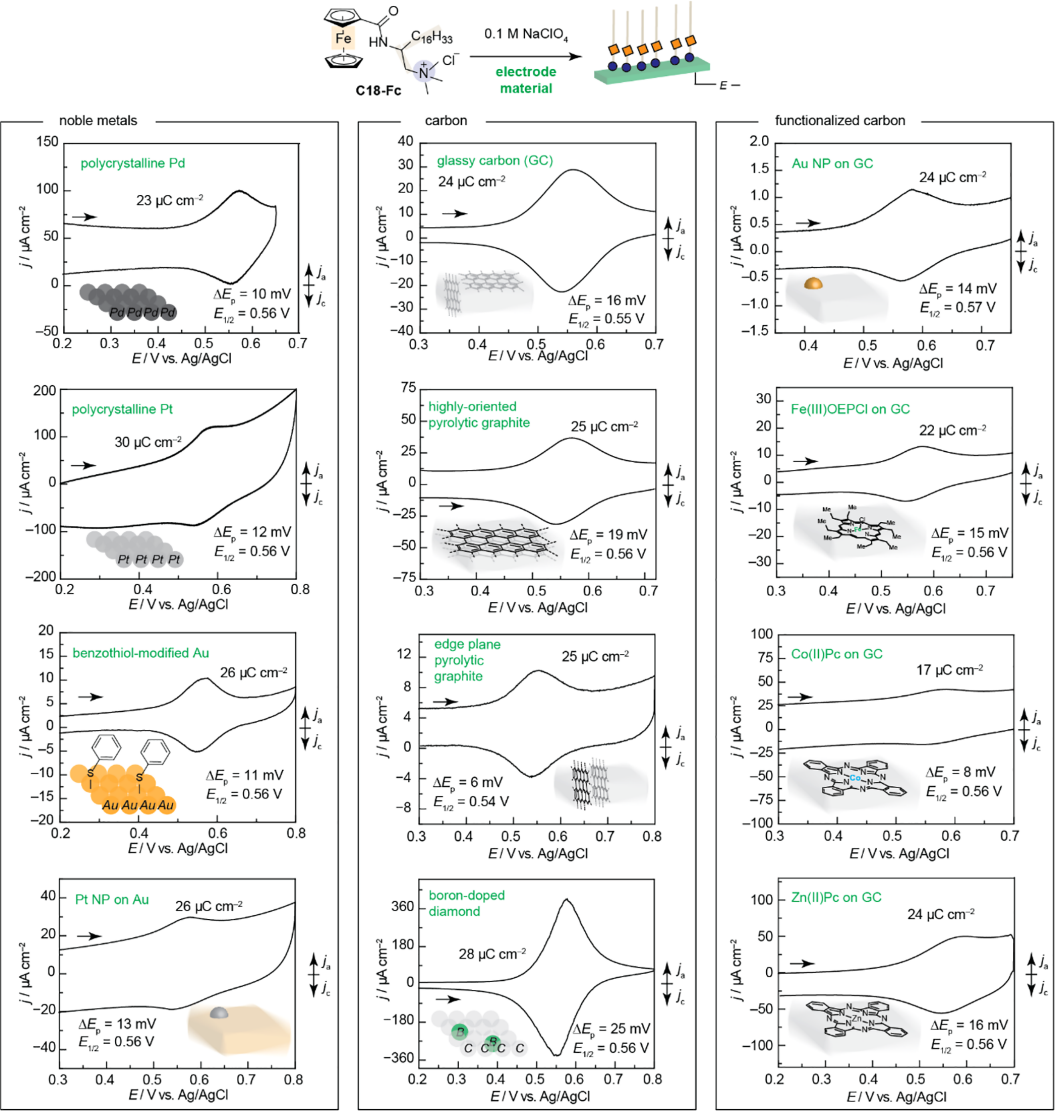
Electrode materials scope, i.e., electrode-orthogonality, of the self-assembled non-covalent layer. (Noble metals—top to bottom) CV of Pd disk at 100 mV s^–1^ in 0.1 M NaClO_4_ containing 200 μM **C18-Fc**. CV of Pt disk at 100 mV s^–1^ in 0.1 M NaClO_4_ containing 67 μM HClO_4_ and 200 μM **C18-Fc**, pH 3.6. CV of benzenethiol-modified Au at ∼0.4 surface coverage at 20 mV s^–1^ in 0.1 M NaClO_4_ containing 200 μM **C18-Fc**. CV of Au containing Pt nanoparticles at 1.7 nmol cm^–2^ surface coverage at 20 mV s^–1^ in 0.1 M NaClO_4_ containing 200 μM **C18-Fc**. (Carbon—top to bottom) CV of glassy carbon foil at 100 mV s^–1^ in 0.1 M NaClO_4_ containing 135 μM **C18-Fc**. HOPG at 20 mV s^–1^ in 0.1 M NaClO_4_ containing 200 μM **C18-Fc**. CV of EPG disk at 20 mV s^–1^ in 0.1 M NaClO_4_ containing 200 μM **C18-Fc**. CV of BDD disk at 1 V s^–1^ in 0.1 M NaClO_4_ containing 150 μM **C18-Fc**. (Functionalized carbon—top to bottom). CV of AuNP-modified glassy carbon disk at ∼0.3 surface coverage at 2 mV s^–1^ in 0.1 M NaClO_4_ containing 135 μM **C18-Fc**. CV of Fe(III)OEPCl (8 nmol cm^–2^ surface coverage) adsorbed on GC at 20 mV s^–1^ in 0.1 M NaClO_4_ containing 200 μM **C18-Fc**. CV of Co(II)Pc (3 nmol cm^–2^ surface coverage) adsorbed on GC at 100 mV s^–1^ in 0.1 M NaClO_4_ containing 200 μM **C18-Fc**. CV of Zn(II)Pc (0.5 nmol cm^–2^ surface coverage) adsorbed on GC at 100 mV s^–1^ in 0.1 M NaClO_4_ containing 200 μM **C18-Fc**. All experiments conducted with a positive direction of scan under N_2_.

As our mechanism for **C18-Fc** layer formation at the Au electrode interface involves the combined non-covalent interactions of electrostatics and hydrophobics, we envisioned that the layers would form over a wide array of different electrode materials. Figure 3 demonstrates this electrode-orthogonality. For all electrode materials, we observe 17–30 μC cm^–2^ of charge passed for the **C18-Fc** oxidation, consistent with monolayer formation (see above), albeit at slightly different concentrations of bulk **C18-Fc**. In addition, for all electrode materials, the anodic and cathodic **C18-Fc** redox features are less than 25 mV, inconsistent with solution-dissolved one-electron redox and consistent with an immobilized Fc redox species. Furthermore, for all materials, the *E*_1/2_ of the layers (*E*_1/2_ 0.54 to 0.57 V) are nearly identical to what we observe on Au, see above, demonstrating that the ∼120 mV positive shift relative to **C2-Fc** is retained on all materials examined. The observation that the electrochemical features diagnostic of a self-assembled layer persists across a wide range of materials suggests that the PZFC values of all electrodes examined are negative of the **C18-Fc***E*_1/2_ value. Indeed, for well-characterized Au, Pt, and Pd materials, the observed *E*_1/2_ of **C18-Fc** is more positive than the reported PZFC ranges at −0.011 to 0.336 V,^84,85^ 0.031 to 0.101 V,^91−95^ and −0.075 to 0.101 V,^96−98^ respectively. Together, these results suggest that the **C18-Fc** forms nearly identical structures to what we characterized on Au.

The self-assembled layer forms on modified polycrystalline transition-metal materials, Figure 3, left column. The layer forms on Pd and Pt electrodes. We note that, for Pt, surface oxides form at potential values competitive with the **C18-Fc** redox feature under the neutral pH conditions examined. We found that the **C18-Fc** redox feature can be revealed by acidifying the electrolyte solution as the Fc redox process is pH-independent, whereas the oxide feature is sensitive to the pH. As we use the Fc redox wave to probe the success of self-assembled layer formation, we are unable to determine if electrode materials that competitively corrode or form surface oxides are compatible. The layer forms on modified Au materials containing ∼45% surface coverage of organic modifiers^99^ (Figure S23). The layer also forms on composite electrodes, such as Pt nanoparticles on Au (1.7 nmol cm^–2^ surface coverage, Figure S24). Together, we show that the non-covalent self-assembled layers form on common transition-metal electrodes that do not form competitive oxide layers or corrode, despite the variation in surface morphologies and functionalization.

The self-assembled layer forms on functionalized and unfunctionalized carbon materials, Figure 3, middle and right columns. The layer is insensitive to the predominant surface-termination of carbon. The layer forms on glassy carbon, highly oriented pyrolytic graphite (HOPG), edge-plate pyrolytic graphite (EPG), and boron-doped diamond electrodes (BDD). We note, for the BDD, charge integration reflective of layer formation was only possible at scan rates >1 V/s, which could suggest multilayer formation on this electrode. The layer forms on carbon materials modified with Au nanoparticles (30% surface coverage, Figure S25). Finally, composite glassy carbon electrodes modified with well-utilized heterogeneous catalysts,^100−102^ such as iron (III) octaethylporphyrin chloride (FeOEPCl, surface coverage, 8 nmol cm^–2^, Figure S26 and Table S7), cobalt phthalocyanine (CoPc, surface coverage 3 nmol cm^–2^, Figure S27 and Table S7), and the control compound, zinc phthalocyanine (ZnPc, surface coverage 0.5 nmol cm^–2^, Figure S28 and Table S7), also exhibit redox features of **C18-Fc** consistent with self-assembled layer formation. Together, these results show the broad scope and electrode-orthogonality of our newly discovered **C18-Fc** self-assembled layers on electrode materials of contemporary interest.

**Figure 4:**
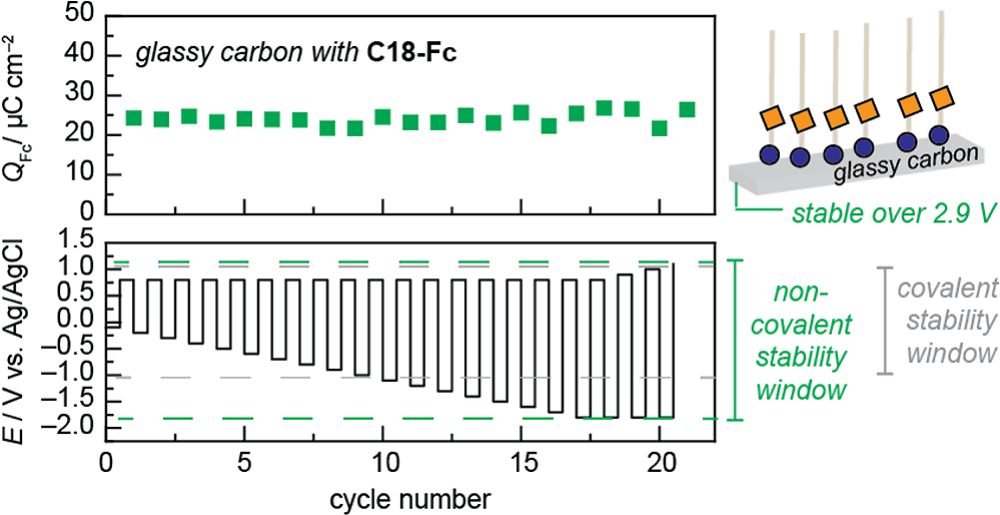
Stability of **C18-Fc** layer on a glassy carbon electrode as a function of the applied potential. (top) Charge integration of **C18-Fc** oxidative wave as a function of (bottom) cyclic voltammogram cycle number with gradually expanding potential window range. The experiment was conducted in 0.1 M NaClO_4_ containing 135 μM **C18-Fc**. The stability range observed is indicated with green dashed lines. Gray dashed lines summarize literature-reported potential-dependent stability ranges for covalent modifications described in the main text.

The wide scope of electrode materials enables the investigation of the stability limits of the **C18-Fc** self-assembled layer using electrodes with wide potential windows. Among the materials investigated, glassy carbon has the widest potential window. Figures 4 and S29 depict that the layer formed on glassy carbon remains intact with a nearly constant integrated charge over a 2.9 V window. We note that SEIRA data taken over this identical expanded potential range on Au surfaces exhibit minimal changes to the observed spectroscopic features (see Figure S30 and Table S8), consistent with the potential-dependent stability observed for the proposed **C18-Fc** self-assembled layer on glassy carbon surfaces in Figure 4. This window is larger than the documented values for thiol (−1.1 to 0.95 V),^18,21^*N*-heterocyclic carbene (−0.4 to 0.6 V),^21^ and isocyanide (−1.15 to 0.95 V)^19,22^ modifications, as well as non-covalent immobilization strategies such as pyrene π–π stacking on carbon (−2.1 to 0.5 V).^25,33^ We note that the potential stability window of the **C18-Fc** self-assembled layer is probed in the presence of **C18-Fc** in bulk solution. Thus, while the requirement to have **C18-Fc** present in the bulk solution limits the layer’s *ex situ* applications, under electrochemical conditions, provided that the synthesized amphiphile is soluble in the electrolyte of interest and does not degrade in the bulk solution, we show that the self-assembled layer is maintained in diverse electrochemical environments (*e.g.*, applied potential, varying electrode materials).

## Conclusions

In this work, we synthesize a series of ammonium-based amphiphilic molecules with appended ferrocene redox probes and investigate their voltammetric response and *in situ* infrared spectra at Au surfaces. Our data mimic those reported for analogous covalently bound ferrocene to Au and are consistent with the formation of an *in situ* self-assembling molecular layer of amphiphiles at the electrified surface that is driven by combined non-covalent electrostatic and van der Waals interactions. Critically, the latter non-covalent interaction enables the layer to remain intact even at applied potential values where electrostatic repulsion should repel the layer. In contrast to covalent systems, the self-assembled non-covalent layer is reversible, i.e., it is easily rinsed off with water, and is independent of the surface chemistry of the electrode material. This non-fouling property of the layer enables the electrode-orthogonality of self-assembly; micromolar concentrations of the amphiphile in bulk solution allow for modification of a variety of electrode materials, enabling its application to electrochemical systems without competitive potential-dependent linkage degradation. Thus, our mechanistic finding enables us to produce self-assembled electrode-orthogonal layers at electrode interfaces, mimicking covalent molecular tuning over a wide range of electrode materials and an expanded potential range.

In all, the non-covalent strategy hijacks the atomistic precision inherent to *ex situ* organic synthesis of amphiphiles with *in situ* non-covalent self-assembly. We anticipate that this generalizable strategy will impact the design of new hybrid architectures, including the localization of a diverse array of redox-active moieties beyond Fc, to fine-tune charge transfer at emerging electrode materials for applications in energy storage and conversion, molecular sensing, and electro-organic synthesis.

## Supporting Information Available

Experimental and computational details and methods; synthetic procedures; electrochemical data; *in situ* spectroscopic data; dynamic light scattering data; quantum chemical calculations; NMR data; HRMS data (PDF)
