# Supplementary material for: Non-Covalent Interactions Mimic the Covalent: An Electrode-Orthogonal Self-Assembled Layer
Source: J Am Chem Soc. 2023 Aug 7;145(32):17734–45. doi: 10.1021/jacs.3c04387 (PMC10436282; doi:10.1021/jacs.3c04387)
Supplement: Supplementary file 1 — ja3c04387_si_001.pdf [file ja3c04387_si_001.pdf]

Supporting Information

Non-Covalent Interactions Mimic the Covalent: An Electrode-Orthogonal Self-Assembled Layer

Deepak Badgurjar,[1,†] Madison Huynh,[1,†] Benjamin Masters,[1] Anna Wuttig[1,*]

[1]Department of Chemistry, University of Chicago, Chicago, IL, 60637, United States
[†]equal contribution

Corresponding author: awuttig@uchicago.edu

Index	***Page***
**1.** General Methods	**S6**
**2.** Electrochemical Methods	**S6**
**3.** Electrode Materials and Preparations	**S6–8**
**4.** Electrolyte and Stock Solution Preparation	**S8**
**5.** Surface-Enhanced Infrared Absorption Spectroscopy (SEIRAS)	**S8**
**6.** Preparation of Au Films for Surface-Enhanced Infrared Absorption Spectroscopy.	**S9**
**7.** Bulk Solid-Phase IR Measurements	**S9**
**8.** Cu Underpotential Deposition	**S9**
**9.** Numerical Frequency Calculations	**S9**
**10.** Determination of Diffusion Coefficients for C18-Fc, C18(C12)-Fc, and C2-Fc	**S9**
**11.** Estimation of Critical Micelle Concentration (CMC) and Surface Tension at the Air-Liquid Interface	**S10**
**12.** Estimation of the Charge Integration for a Monolayer Coverage of C18-Fc and C18(C12)-Fc.	**S10**
**13.** Rinse Test	**S10–11**
**14.** Synthetic Scheme	**S12**
**15.** Synthetic Procedures	**S13–18**
**Table S1:** Critical micelle concentrations (CMC) of compounds in ultrapure water determined by dynamic light scattering	**S19**
**Table S2:** Densities and surface tensions of C18-Fc in ultrapure water and 0.1 M $NaClO_4$	**S19**
**Table S3:** Redox potentials for C2-Fc, C18-Fc, and C18(C12)-Fc in 0.1 M $NaClO_4$ in H_2O	**S19**
**Table S4:** Peak currents, redox potentials, and peak potential separation for C2-Fc, C18-Fc, and C18(C12)-Fc in 0.1 M $TBAClO_4$ in acetonitrile	**S20**
**Table S5:** Peak currents, redox potentials, and peak potential separation for C2-Fc, C18-Fc, and C18(C12)-Fc in 0.1 M $NaClO_4$ in H_2O	**S20**

Table S6: Coordinates for optimized structure of C18-Fc **S21–24**

Table S7: Material loading on glassy carbon electrodes **S24**

Table S8: Comparison of the IR spectroscopic features in the presence of C18-Fc on Au film at 1.1 V and −1.8 V vs Ag/AgCl in 0.1 M $NaClO_4$ **S25**

Figure S1: Plot of the intensity of scattered light obtained with various concentrations of C18-Fc used to measure critical micelle concentration **S26**

Figure S2: Plot of the intensity of scattered light obtained with various concentrations of C18(C12)-Fc used to measure critical micelle concentration **S26**

Figure S3: Peak current of cyclic voltammograms obtained at varying scan rates for C18-Fc, C2-Fc, and C18(C12)-Fc in 0.1 M $TBAClO_4$ in acetonitrile used to estimate the diffusion coefficients **S27**

Figure S4: Cyclic voltammogram of 75 µM C18-Fc on an annealed Au disk electrode in 0.1 M $NaClO_4$ solution without and with FFT smoothing **S27–28**

Figure S5: Cyclic voltammograms of an annealed Au disk electrode in aqueous 0.1 M $NaClO_4$ electrolyte solution before adding C18-Fc, C2-Fc, and C18(C12)-Fc **S28**

Figure S6: Differences in the peak potential of C2-Fc as a function of the scan rate at 75 µM, 135 µM, and 200 µM of C2-Fc **S29**

Figure S7: Cyclic voltammograms of an annealed Au disk electrode in aqueous 0.1 M $NaClO_4$ electrolyte with and without 75 µM C18(C12)-Fc **S29**

Figure S8: Cyclic voltammograms of 75 µM C2-Fc, C18(C12)-Fc, or C18-Fc in 0.1 M $NaClO_4$ in H_2O collected at 100 mV s^{-1} on a Au working disk electrode **S30**

Figure S9: Peak current vs. scan rate and square root of the scan rate for C18-Fc and C18(C12)-Fc **S31**

Figure S10: Cyclic voltammograms of an annealed Au disk electrode in aqueous 0.1 M $NaClO_4$ electrolyte with and without 75 µM C18-Fc **S32**

Figure S11: Cyclic voltammograms of 75 µM C18-Fc in aqueous 0.1 M $NaClO_4$ electrolyte solution recorded at varying scan rates on an annealed Au electrode **S32**

Figure S12: Geometry-optimized structure of C18-Fc **S33**

Figure S13: Cyclic voltammograms of 1st and 140th scans of 75 µM C18-Fc in aqueous 0.1 M $NaClO_4$ electrolyte solution on an annealed Au disk electrode **S33**

Figure S14: Cyclic voltammograms of a rinsed Au disk electrode in aqueous 0.1 M $NaClO_4$ electrolyte with and without 200 µM C18-Fc on annealed Au disk electrode **S34**

Figure S15: Cyclic voltammograms of a freshly prepared SEIRAS-active Au film and Au disk electrode in 0.5 M H_2SO_4 containing 7.5 mM $CuSO_4$ **S34–35**

Figure S16: Cyclic voltammetry and simultaneously collected SEIRA spectra on a Au film in the presence of 0.1 M $NaClO_4$ in D_2O and 200 μM C18-Fc **S35**

Figure S17: Computed IR spectrum of C18-Fc **S35**

Figure S18: Comparison of simultaneously collected SEIRA spectra on a Au film in the presence of 0.1 M $NaClO_4$ in D_2O and 200 μM C18-Fc versus 0.1 M $NaClO_4$ in H_2O and 200 μM C18-Fc and C2-Fc bulk IR spectrum **S36**

Figure S19: Integrated band intensities of selected peaks with changing applied potential from SEIRA spectra on a Au film in 0.1 M $NaClO_4$ in D_2O with 200 μM C18-Fc **S37**

Figure S20: Cyclic voltammetry and simultaneously collected absolute and differential SEIRA spectra in 0.1 M $NaClO_4$ in H_2O with 200 μM C18-Fc, second consecutive scan **S38**

Figure S21: Integrated band intensities of selected peaks with changing applied potential from SEIRA spectra on a Au film in 0.1 M $NaClO_4$ in H_2O with 200 μM C18-Fc, second consecutive scan **S39**

Figure S22: Cyclic voltammetry and simultaneously collected SEIRA spectra on a Au film in the presence of 0.1 M $NaClO_4$ in H_2O and 200 μM C2-Fc **S40**

Figure S23: Cyclic voltammogram of Au disk electrode modified with benzenethiol **S40**

Figure S24: Cyclic voltammograms of electrodeposited Pt nanoparticles on Au electrode in aqueous 0.5 M H_2SO_4 electrolyte solution **S41**

Figure S25: Cyclic voltammograms of electrodeposited Au nanoparticles on glassy carbon electrode in aqueous 0.1 M HCl electrolyte solution **S41**

Figure S26: Cyclic voltammogram of glassy carbon disk electrode modified with FeOEPCl recorded in electrolyte solution of 0.1 M TBAP in acetonitrile **S42**

Figure S27: Cyclic voltammogram of glassy carbon disk electrode modified with cobalt phthalocyanine (CoPc) recorded in electrolyte solution of 0.1 M TBAP in acetonitrile **S42**

Figure S28: Cyclic voltammogram of glassy carbon disk electrode modified with zinc phthalocyanine (ZnPc) recorded in electrolyte solution of 0.1 M TBAP in acetonitrile **S43**

Figure S29: Cyclic voltammogram of glassy carbon foil electrode across increasing potential ranges in the presence of 135 μM C18-Fc in 0.1 M $NaClO_4$ **S43**

Figure S30: Cyclic voltammetry and simultaneously collected SEIRA spectra in 0.1 M $NaClO_4$ in H_2O taken over a wide potential window on Au film in the presence of C18-Fc **S44**

Structural Characterization of Synthesized Compounds **S45–S78**

References **S79–S82**

Experimental Procedures

1. General Methods. Reagents were purchased from commercial sources and used without further purification unless otherwise noted. Standard Schlenk line techniques were utilized for synthetic manipulations unless otherwise noted. Dimethylformamide (DMF), dichloromethane (CH_2Cl_2), acetonitrile (MeCN), and tetrahydrofuran (THF) were dried and purified using a solvent purification system (PureSolv MD 5, INERT Corporation) under N_2. Thin-layer chromatography (TLC) analysis of reaction mixtures were performed using Merck silica gel 60 F254 TLC plates and visualized using iodine, ninhydrin or *p*-anisaldehyde stain, or UV light. Column chromatography was carried out using Macherey-Nagel Silica Gel 60 Å, 0.04–0.063 mm. ^{1}H and ^{13}C experiments were conducted using Bruker Avance III HD nanobay 400 MHz and Bruker Avance III HD 500 MHz instruments. Peaks were referenced using residual solvent peaks e.g., for ^{1}H-NMR $CDCl_3$ (δ = 7.26 ppm) or DMSO-d_6 (δ = 2.50 ppm) and ^{13}C NMR in $CDCl_3$ (δ = 77.16 ppm) or DMSO-d_6 (δ = 39.52 ppm) solvents as an internal reference. High resolution mass spectrometry (ESI/APCI ion source) was conducted using an Agilent 6224 TOF-MS spectrometer instrument and chloroform/MeCN as the solvents. Dynamic light scattering data were collected using a Wyatt Möbiuz dynamic light scattering instrument.

2. Electrochemical Methods. Electrochemical experiments were conducted using a Gamry Reference 620 Potentiostat, a Ag/AgCl reference electrode (eDAQ, leakless), a high surface area Pt-mesh counter electrode (Alfa Aesar, 99.997 %), and various working electrodes (see Electrode Materials). The Ag/AgCl electrode was stored in Millipore Type 1, 18.2 MΩ, water between measurements and was periodically checked relative to a pristine Ag/AgCl to ensure against potential drift. All experiments were performed at 24±1℃. Electrode potentials reported are referenced against the SHE standard using the conversion, $E_{\mathrm{V\ vs\ Ag/AgCl}} + 0.205\ \mathrm{V} = E_{\mathrm{V\ vs\ SHE}}$ and the Fc/Fc^{+} standard the conversion, $\mathrm{E_{V\ vs\ Ag/AgCl}} - 0.345\ \mathrm{V} = \mathrm{E_{V\ vs\ Fc/Fc+}}$ (measured in acetonitrile). For each experiment utilizing the Gamry Potentiostat, an initial starting point for the uncompensated Ohmic loss was measured using the Gamry Instruments Framework software version 7.8.4 (~200-300 Ω). A percentage of the measured value (85%) was inputted manually into the iR compensation PF option in the Gamry Instruments Framework software workstation. Current density values are reported relative to the geometric surface area of the working electrode. An electrochemical cell was utilized and held 10 mL of electrolyte (James Glass Inc.). Cyclic voltammograms data were processed using Origin software. To reduce the noise that PF correction introduced, FFT smoothing (point of window = 15) was used to improve the readability of the CV figures but leave electrochemical features qualitatively unchanged (**Figure S4**).

3. Electrode Materials and Preparations.

Polycrystalline Au working electrodes were purchased from CH Instruments (2 mm diameter, 0.0314 cm^2 area). Au electrodes were polished with alumina slurry (1.0 µm in ultrapure water, Millipore Type 1, 18.2 MΩ) and sonicated in ultrapure water. Au disk electrodes were annealed for approximately 5 seconds using a flame from a butane torch. To normalize for a varying electrochemical double layer after each annealing, the cyclic voltammograms of Au electrodes in aqueous 0.1 M $NaClO_4$ were divided by a scaling factor ("n") calculated for each background CV and reported in **Figure 1b** (**Fig. S5a**).

Polycrystalline Pd working electrodes were purchased from BASi (3 mm diameter, 0.0707 cm^2 area). Pd electrodes were polished with alumina slurry and sonicated in ultrapure water.

Polycrystalline Pt working electrodes were purchased from CH instruments (2 mm diameter, 0.0314 cm^2 area). Pt electrodes were polished with alumina slurry and sonicated in ultrapure water.

A monolayer of benzenethiol was prepared on a polycrystalline Au disk electrode according to a previously reported procedure.[1] Commercially available benzenethiol (Sigma-Aldrich, 99%) was used without further purification. The Au electrode was immersed for 1–2 minutes in a freshly prepared 1 mM solution of benzenethiol in ultrapure water, stirring with a using magnetic bead for ~30 seconds. The Au electrode was then removed and placed in a glass vial containing 15 mL ethanol (200 proof) and stirred at 300–400 rpm for 2–3 min to remove excess unbound benzenethiol. The prepared electrode was placed in an electrochemical cell with 0.5 M KOH, and CVs were recorded at a scan rate of 10 mV s^{-1} with a Hg/HgO reference electrode and a Pt mesh counter electrode (**Fig. S23**). Surface coverage was calculated to be 22.8 μC cm^{-2} using charge integration, approximately ~45% of reported values.[1]

Pt nanoparticles were electrodeposited on a polycrystalline Au rotating disk electrode (Pine Research, 5 mm diameter, 0.196 cm^2 geometric surface area, no rotation) according to a previously reported procedure.[2] A gold electrode was polished for 3 minutes in alumina slurry and sonicated. To electrodeposit Pt nanoparticles, chronoamperometry was performed at 0.5 V vs Ag/AgCl for 60 seconds and 0.1 V vs Ag/AgCl for 60 seconds in the presence of 0.25 mM H_2PtCl_6 with N_2-saturated 0.05 M H_2SO_4 as an electrolyte. The prepared electrode was rinsed with ultrapure water. The surface concentration of the Pt nanoparticles was calculated to be 1.16×10^{-9} mol cm^{-2} based on integration of the two Pt–H features (from –0.033 V vs Ag/AgCl to –0.236 V vs Ag/AgCl) in CVs taken in N_2-saturated 0.5 M H_2SO_4 (**Fig. S24**).

Glassy carbon foil working electrodes were purchased from Goodfellow (0.3 cm^2 geometric surface area, 0.5 mm thickness). Glassy carbon foil electrodes were rinsed with ultrapure water and connected to the working electrode lead using stainless steel alligator clips.

Highly oriented pyrolytic graphite (HOPG) was purchased from SPI (brand grade SPI-2, 10 mm × 10 mm × 1 mm). HOPG working electrodes were sonicated for 1 min in CH_2Cl_2 and dried. Both sides of the HOPG surface were peeled off using Scotch tape as previously reported.[3,4] Excess visible graphite flasks on top layer were removed using Kimwipes wet with ultrapure water. HOPG working electrodes (1×1 cm^2 geometric surface area) were connected to the working electrode lead using stainless steel alligator clips, and 0.5 cm^2 of the electrode was submerged in the electrolyte. Ten CV cycles were recorded for each experiment at scan rate of 20 mV s^{-1} and 100 mV s^{-1} to obtain reproducible data.

Edge-plane pyrolytic graphite (EPG) electrodes were purchased from BASi (3 mm diameter, 0.0707 cm^2 area). EPG electrodes were polished with alumina slurry and sonicated in ultrapure water.

Boron-doped diamond (BDD) working electrodes were purchased from BioLogic (doping level 500–1000 ppm, 3 mm diameter, 0.0707 cm^2 area). BDD electrodes were polished with diamond slurry (Buehler MetaDi polycrystalline diamond suspension, 0.05 μm) and sonicated in ultrapure water.

Au nanoparticles were electrodeposited on a glassy carbon rotating disk electrode (Pine Research, 5 mm diameter, 0.196 cm^2 geometric surface area, no rotation) according to a previously reported procedure.[5] A glassy carbon electrode was polished for 3 minutes with alumina slurry and 3 minutes with diamond slurry and sonicated in ultrapure water. To electrodeposit Au nanoparticles,

chronoamperometry was performed at –0.5 V vs Ag/AgCl for 60 seconds in 0.1 mM $NaAuCl_4$ with N_2-saturated 0.1 M H_2SO_4 as an electrolyte. The prepared electrode was rinsed with ultrapure water. The surface coverage of the Au nanoparticles was calculated to be 32.5% based on integration of the oxidative stripping wave in N_2-saturated 0.1 M HCl (**Fig. S25**).

Glassy carbon rotating disk electrodes (Pine Research, 5 mm diameter, 0.196 cm^2 geometric surface area, no rotation) were modified with cobalt(II) phthalocyanine (Sigma-Aldrich, β-form, dye content 97 %, CoPc), Zinc phthalocyanine (Sigma-Aldrich, dye content 97 %, ZnPc) and 2,3,7,8,12,13,17,18-Octaethyl-21*H*,23*H*-porphine iron(III) chloride (Sigma-Aldrich, FeOEPCl) without any further purification. The glassy carbon electrodes were modified following a previously reported procedure.[6] A dye ink was prepared using CoPc (8.3 mg), ZnPc (7.9 mg), and FeOEPCl (1.3 mg) dissolved separately in 0.8 mL CH_2Cl_2, followed by an additional 0.15 mL ethanol (200 proof) and 0.05 mL Nafion perfluorinated resin (5% wt. in aliphatic alcohol and water 45%, Sigma-Aldrich). These solutions were sonicated for 5 min and 5 µL of the ink was drop cast on glassy carbon. We optimized the above material conditions after multiple trials to avoid high double-layer capacitance in CV measurements that convolute observable redox features. See **Fig. S26–S28** for redox features of ZnPc (II/I), Co (II/I) and FeOEPCl (III/II) and **Table S7** for calculated electroactive catalyst loading.

4. Electrolyte and Stock Solution Preparation. High purity $NaClO_4$ hydrate (99.99% trace metals basis, Sigma Aldrich) and Millipore Type 1, 18.2 MΩ, water were used for all aqueous electrolyte preparation. The same stock solution of 0.1 M $NaClO_4$ was used for all comparison studies, e.g., scan rate dependence, concentration dependence. To prepare the **C18-Fc** stock solution, approximately 2 to 5 mg of **C18-Fc** or **C18(C12)-Fc** was added to 1 mL of ultra-pure water. The stock solution was manually shaken at room temperature to dissolve, avoiding any sonication or heating. The stock solution was stored in refrigerator and used for electrochemical measurements within 12 hours to avoid decomposition. We note that 75 µM of each monomer was examined because the limited solubility of the species in aqueous media precluded investigation at the identical concentration values utilized for MeCN in **Figure 1**.

For the H/D exchange SEIRAS experiment, 4 mg of **C18-Fc** was dissolved in 1 mL of D_2O and stirred for 4 days at room temperature, resulting in a stock solution with a concentration of 6.96×10^{-3} M. Separately, 0.61 g $NaClO_4$ (anhydrous) was dissolved in 50 mL of D_2O, resulting in electrolyte with a concentration of 0.1 M. In the spectroelectrochemical cell, 12 mL of 0.1 M $NaClO_4$ in D_2O was purged with N_2 for 15 min prior to measurement. 60 to 125 µL of **C18-Fc** stock solution was added into the cell to obtain concentrations of 35 µM to 200 µM.

5. Surface-Enhanced Infrared Absorption Spectroscopy (SEIRAS). SEIRA spectra were recorded in an attenuated total reflection (ATR) configuration using a Nicolet iS50 FTIR spectrometer equipped with a HgCdTe (MCT) detector (Ref/01 Gain Setting) and a PIKE VeeMax III accessory (incident angle of 60˚). The Nicolet spectrometer was operated in series mode with optical velocity of 1.8988 cm s^{-1}. Spectra were sequentially acquired with a spectral resolution of 4 cm^{-1} at every 19.2(8) s interval. A single beam spectrum (32 scans) collected at the starting potential in the absence of any substrates (i.e., in the presence of 0.1 M $NaClO_4$ in water) was used as the reference spectrum. All ATR-SEIRA spectra are reported in absorbance units defined as $A = -\log (I/I_0)$, where I and I_0 stand for the sample and reference single-beam spectra, respectively. Data analysis was conducted using the OMNIC version 9.12.928 software. The PIKE Jackfish J2 spectroelectrochemical cells were used.

6. Preparation of Au Films for Surface-Enhanced Infrared Absorption Spectroscopy. Au films were prepared on undoped Si prisms (PIKE) using the "double deposition method" as previously detailed.[7,8] The geometric surface area of the SEIRAS film exposed to the electrolyte was 0.71 cm^2. Following the deposition, the Au-coated Si prism was assembled into the PIKE Jackfish J2 spectroelectrochemical cell and cleaned using an electrochemical procedure. Prior to use, the Au film was cycled in 0.1 M H_2SO_4 from –0.10 V to 1.65 V vs Ag/AgCl at 100 mV s^{-1} for 5 continuous cycles and –0.10 V to –1.20 V vs Ag/AgCl at 100 mV s^{-1} for 5 continuous cycles. Following, the Au film was washed 3 to 5 times with ultrapure water. The electrolyte in the cell was replaced with 0.1 M $NaClO_4$ and purged with N_2 for at least 15 minutes. Prior to data collection, the Au film was cycled in 0.1 M $NaClO_4$ from –0.10 V to 1.65 V vs Ag/AgCl at 100 mV s^{-1} for 5 continuous cycles and –0.10 V to –1.20 V vs Ag/AgCl at 100 mV s^{-1} for 5 continuous cycles. The electrolyte was replaced with fresh 0.1 M $NaClO_4$ and purged with N_2 for at least 15 minutes. Around 3 to 5 cycles of cyclic voltammetry from –0.10 V to 0.80 V vs Ag/AgCl at 2 mV s^{-1} were performed to obtain a stabilized signal from the Au film in 0.1 M $NaClO_4$. Before recording the IR spectrum, the Au film was held for at least 200 sec at –0.10 V vs Ag/AgCl. All experiments were conducted under N_2. All background spectra were recorded at –0.10 V vs Ag/AgCl over various time intervals.

7. Solid-Phase IR Measurements. Spectra were recorded using a Nicolet iS50 FTIR spectrometer equipped with a HgCdTe (MCT) detector (Ref/01 Gain Setting). The Nicolet spectrometer was operated in single scan mode with optical velocity of 0.9494 cm s^{-1}. Spectra were acquired with a spectral resolution of 4 cm^{-1} with a single beam spectrum (32 scans). KBr (60-100 mg, FT-IR grade, ≥99% trace metals basis) pellets were prepared with a custom-built wrench-operated pellet press. Background spectra were recorded with visually transparent and stable pellets.

8. Cu Underpotential Deposition. Cu underpotential deposition on Au disk electrodes and Au SEIRAS-active films used in this study were characterized by a previously reported procedure.[9]

9. Numerical Frequency Calculations. Density functional theory calculations to compute numerical frequencies were performed using ORCA 5.0.1. The B3PW91 functional and its associated extended basis set were used.[10] The initial structure of **C18-Fc** was built in Avogadro 1.2.0. The geometries of these structures were optimized, and numerical frequencies were calculated in ORCA (**Figure S12** and **Table S6**). Normal modes were visualized from the output Hessian using Avogadro.

10. Determination of Diffusion Coefficients for C18-Fc, C18(C12)-Fc, and C2-Fc. A 15 mL solution of 0.1 M $TBAClO_4$ in acetonitrile was prepared and sparged with N_2 in an electrochemical cell for 15 minutes. 1 mM of the analyte was added, and the electrolyte solution was stirred and sparged with N_2 until the solution reached a final volume of 10 mL. Cyclic voltammetry measurements were taken at scan rates of 0.05, 0.1, 0.2, 0.5, 1, 2, and 5 V s^{-1}. The electrolyte solution was stirred for 2 minutes and allowed to settle for 3 minutes between each scan. Using the Randles–Ševčík equation,[11,12] the diffusion coefficient was calculated to be 1.87 × 10^{-5} cm^2 s^{-1} for **C2-Fc**, 0.625 × 10^{-5} cm^2 s^{-1} for **C18-Fc**, and 1.01 × 10^{-5} cm^2 s^{-1} for **C18(C12)-Fc** by plotting peak current i_p (A) versus the square root of scan rate $\nu^{1/2}$ ($V^{½}$ $s^{-½}$) and measuring the slope,[13]

$$i_p = \left| 0.4453nFAC^0 \left(\frac{nFD}{RT}\right)^{\frac{1}{2}} \right| v^{\frac{1}{2}}$$

where n = 1 electron, F = Faraday constant (96485 C mol^{-1}), A = 0.031415 cm^2, $C^0 = 1 \times 10^{-6}$ mol cm^{-3}, R = gas constant (8.314 J mol^{-1} K^{-1}), and T = 298 K. Data for slopes are reported in **Fig. S3**. We note that 1 mM of each monomer was examined to obtain sufficient voltametric signal above the background capacitive feature, including those reported in **Figure 1a**.

11. Estimation of Critical Micelle Concentration (CMC) and Surface Tension at the Air-Liquid Interface. CMC calculated using dynamic light scattering (DLS). **C18-Fc** 4.8 mg and **C18(C12)-Fc** 4.3 mg were dissolved each in 1 mL of ultrapure water (stock concentration of 8.35 × 10^{-3} and 7.48 × 10^{-3} M, respectively). Two sets of eight to ten samples were prepared, concentrations ranging from 2.5 × 10^{-5} to 2.19 × 10^{-4} M and 3.72 × 10^{-5} to 5.54 × 10^{-4} M for **C18-Fc** and **C18(C12)-Fc**, respectively. The intensity of scattered light (in kilo counts per sec, kCnt/s) was recorded and plotted as a function of the monomer concentration. CMC was calculated at the intersection of curves, as showed in **Fig. S1** and **S2.** An Anton Paar, DMA 4500M density meter was used to measure the density of ultrapure water and 0.1 M $NaClO_4$. The obtained values, 0.99709 and 1.00477 g/cm^3 at 25°C for ultrapure water and 0.1 $NaClO_4$, respectively, were consistent with previous reports.[14,15] **C18-Fc** 5.1 mg was dissolved in 1 mL of ultrapure water (stock solution of 8.87 × 10^{-3} M) to prepare 200 μM in ultrapure water and 0.1 M $NaClO_4$. The densities of these samples are tabulated in **Table S2**. These values were used to calculate the surface tension of **C18-Fc** and measured using a DSA 100 Drop Shape Analyzer KRÜSS GmbH system, with a pendant drop method. **C18-Fc** 3.9 mg was dissolved in 1 mL ultrapure water (stock concentration of 6.78 × 10^{-3} M) to prepare 200 μM in ultrapure water and 0.1 M $NaClO_4$. Measured surface tensions at 27.3°C are tabulated in **Table S2**.

12. Estimation of the Charge Integration for a Monolayer Coverage of C18-Fc and C18(C12)-Fc. Cyclic voltammograms of **C18-Fc** and **C18(C12)-Fc** on a gold disk electrode at concentrations of 35, 75, 135, and 200 μM were taken at 2 mV s^{-1}. The experimental values for total charge transferred were calculated by integrating the oxidative peak with respect to potential scanned over that region, which was normalized for the scan rate within the Gamry Echem Analyst software workstation. The total charge transfer was normalized for electrochemically active surface area by dividing by the area of the gold disk electrode (0.0314 cm^2).

Following the literature approach of estimating surface coverage via measuring the cross-sectional area of the surface-bound species[16], the geometry optimized structure of **C18-Fc** (**Fig. S12**) was used to model the cross-sectional area as a rectangle with calculated length from ferrocene to the ammonium group, 10.33 × 10^{-8} cm, and width as the diameter of ferrocene, 6.6 × 10^{-8} cm. Thus, each molecule occupies 6.82 × 10^{-15} cm^2, which corresponds to a surface coverage of 2.44 × 10^{-10} mol cm^{-2}. By multiplying by Faraday's constant, the estimated charge transferred for a monolayer of **C18-Fc** is 23.5 μC cm^{-2}.

13. Rinse Test. Au disk electrode (CH Instruments, 2 mm diameter, 0.0314 cm^2 area) electrode was polished with alumina slurry in ultrapure water and annealed as mentioned in Section 3. A stock solution of **C18-Fc** was prepared according to Section 4. 2.7 mg of **C18-Fc** was dissolved in 1 mL ultrapure water to make a stock solution of 4.7 × 10^{-3} M. Two separate electrochemical cells were set up each containing 10 mL of N_2-saturated 0.1 M $NaClO_4$ electrolyte. In the first cell, CVs of 0.1 M $NaClO_4$ were recorded, followed by the addition of an aliquot 0.44 mL of stock solution to obtain 200 μM **C18-Fc**. After recording the CV, the Au disk electrode was carefully taken out of the cell and suspended in a glass vial containing 15 mL ultrapure water without touching the bottom of the vial or the stir bar. The water was stirred for 10 min at approximately

450 rpm. This electrode was then placed in the second cell with 0.1 M $NaClO_4$. CVs were recorded at a scan rate of 2 mV s^{-1} with Ag/AgCl as the reference and Pt mesh as the counterM electrode. This electrode was then placed back in the first cell that contained 200 μM **C18-Fc** and CVs were recorded in the same conditions. CVs are shown in **Figures 1d** and **S14**.

14. Synthetic Scheme for C18-Fc, C18(C12)-Fc, and C2-Fc Monomer Synthesis.

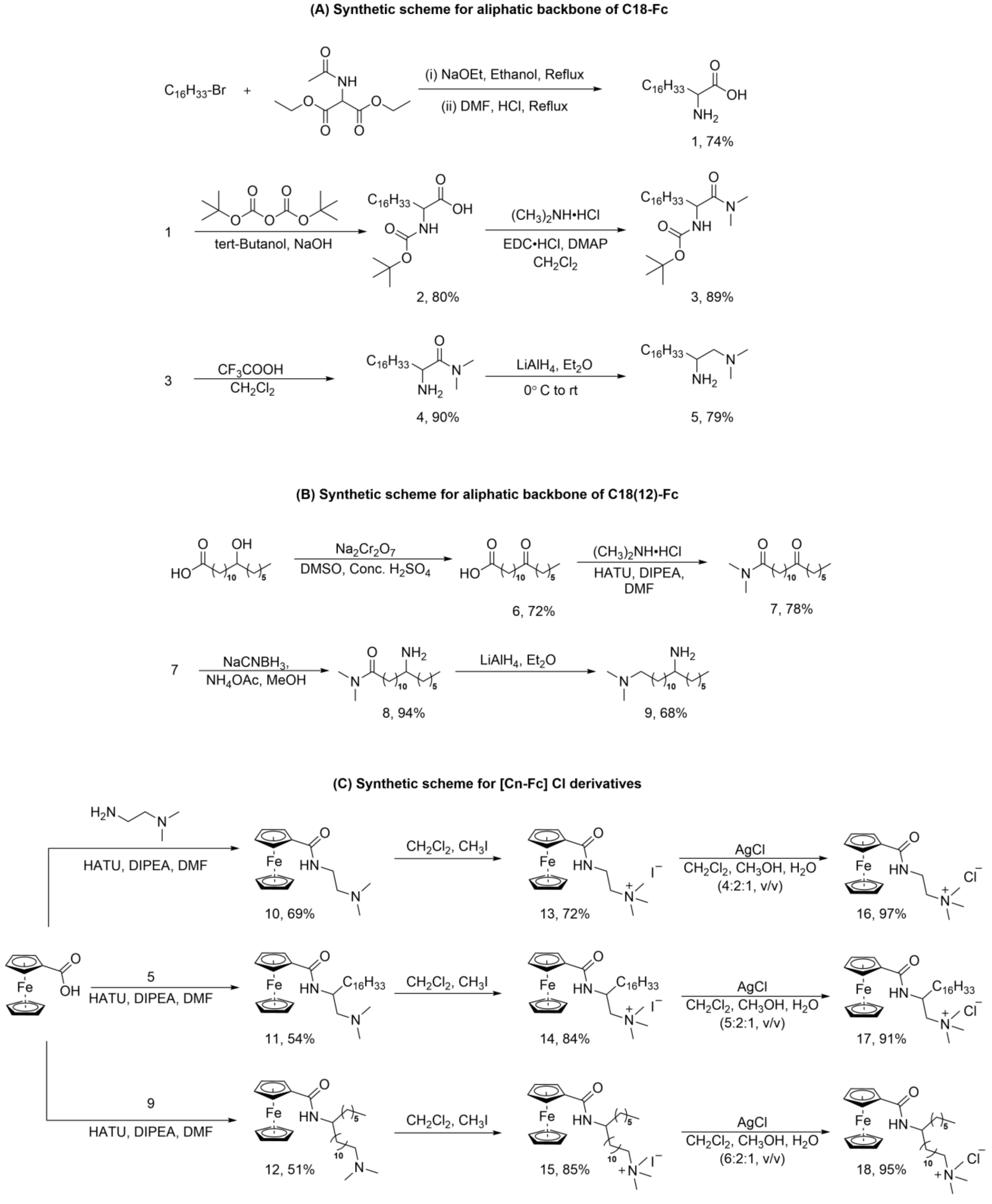

Scheme S1. Synthetic scheme for compounds **1** to **18**. Detailed synthesis procedures are described in Section 15. All reported yields are isolated yields.

15. Synthetic Procedures

Preparation of 2-aminooctadecanoic acid (1). Diethyl 2-acetamidomalonate (21 g, 97 mmol) was added into 150 mL ethanol and was stirred for 30 minutes. 1-bromohexadecane (35 g, 116 mmol) was added and the reaction mixture was refluxed for 24 h. After cooling, the reaction mixture was poured into ice-cold water and the precipitate was filtered out and washed with cold water. This solid was dissolved in 15 mL DMF and 100 mL HCl (36%) and stirred for 36 h. The reaction mixture was cooled to room temperature and poured into ethanol/water (2/1) and neutralized with ammonium hydroxide to obtain a solid compound. For better yield, this crude compound was crushed, dried, and washed with hexanes and used for the next reaction without further purifications. Crude yield: 21 g, 74% Data consistent with literature-reported precedent.[17] ESI-MS Calcd. for $C_{18}H_{37}NO_2$ $[M]^+$, 299.2824 found 299.2808 m/z.

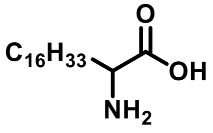

Preparation of 2-((*tert*-butoxycarbonyl)amino)octadecanoic acid (2): Compound **1** (15 g, 50 mmol) was dissolved in 120 mL *tert*-butanol in a 1 L round bottom flask. Around 10 g of NaOH was dissolved in 180 mL of water and added into the reaction mixture. Di-*tert*-butyl dicarbonate (Boc_2O, 17.2 mL, 75 mmol) was added dropwise to the reaction mixture at 0-4 °C, maintaining pH ~13 for 2 hours and stirring for 12 h. After completion of the reaction, citric acid was added portion-wise, and the mixture was maintained at pH ~3 and stirred for 30 minutes. The reaction mixture was extracted with ethyl acetate/water (500 mL × 3), and the ethyl acetate layer was dried over Na_2SO_4 and then concentrated *in vacuo*. 20 mL of MeCN was added to the crude product and was kept at room temperature to obtain a light-yellow crystalline product. Yield: 16 g, 80%. ^{1}H NMR (400 MHz, DMSO-d_6) δ 12.37 (s, 1H), 7.00 (d, J = 8.1 Hz, 1H), 3.84 (td, J = 8.8, 4.8 Hz, 1H), 1.37 (s, 9H), 1.23 (s, 30H), 0.89 – 0.81 (m, 3H). ^{13}C NMR (101 MHz, DMSO-d_6) δ 174.27, 155.56, 77.85, 53.32, 31.29, 30.76, 29.03, 29.00, 28.91, 28.85, 28.70, 28.49, 28.19, 25.48, 22.09, 13.93. ESI-MS Calcd. for $C_{23}H_{45}NO_4$ $[M]^+$, 399.3349 found 399.3354 m/z.

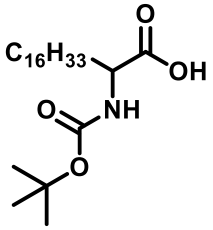

Preparation of *tert*-butyl (1-(dimethylamino)-1-oxooctadecan-2-yl)carbamate (3): Compound **2** (10 g, 25 mmol) was dissolved in 300 mL anhydrous dichloromethane, followed by 4-dimethylaminopyridine (DMAP, 15.3 g, 125 mmol), 1-(3-dimethylaminopropyl)-3-ethylcarbodiimide hydrochloride (EDC•HCl, 24 g, 125 mmol), and dimethylamine hydrochloride (2.2 g, 27.5 mmol) addition at ~0 °C. The reaction mixture was stirred at room temperature for 10 h under nitrogen and was monitored using thin layer chromatography (TLC). The resulting reaction mixture was extracted with dichloromethane and water (500 mL × 3) and dried over Na_2SO_4 and then concentrated *in vacuo.* The crude product was purified by silica gel chromatography using hexanes:ethyl acetate (10:1). Yield: 9.5 g, 89%. ^{1}H NMR (400 MHz, $CDCl_3$) δ 5.35 (d, J = 8.8 Hz, 1H), 4.60 (td, J = 8.4, 4.8 Hz, 1H), 3.08 (s, 3H), 2.96 (s, 3H), 1.44 (s, 9H), 1.39 – 1.23 (m, 30H), 0.88 (t, J = 6.8 Hz, 3H). ^{13}C NMR (101 MHz, $CDCl_3$) δ 173.01,

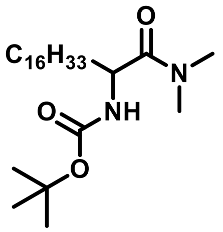

156.06, 79.88, 50.60, 37.56, 36.16, 33.91, 32.41, 30.18, 30.17, 30.14, 30.12, 30.03, 29.96, 29.90, 29.85, 28.86, 25.72, 23.18, 14.60. ESI-MS Calcd. for $C_{25}H_{50}N_2O_3$ $[M]^+$, 426.3821 found 426.3824 m/z.

Preparation of 2-amino-*N,N*-dimethyloctadecanamide (4): Compound **3** (9 g, 21.1 mmol) was

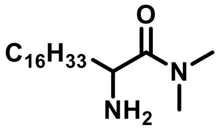

dissolved in 120 mL of anhydrous dichloromethane and 30 mL trifluoroacetic acid was added dropwise at ~0 °C under nitrogen. The resulting mixture was stirred at room temperature for 6 h and monitored using TLC. After completion, the reaction mixture was diluted with 150 mL dichloromethane and quenched with saturated $NaHCO_3$ in water at 0 °C (*caution*: gas evolution!) and extracted with dichloromethane (500 mL × 3) and water. The combined organic layer was dried over Na_2SO_4 and evaporated *in vacuo* to obtain a white solid compound. This solid compound was used for the next reaction without further purification. Yield: 6.2 g, 90%. ^1H NMR (400 MHz, $CDCl_3$) δ 3.69 (m, 1H), 3.03 (s, 3H), 2.98 (s, 3H), 1.79 (s, 2H), 1.64 – 1.19 (m, 30H), 0.88 (t, J = 6.7 Hz, 3H). ^{13}C NMR (101 MHz, $CDCl_3$) δ 175.78, 51.29, 36.94, 35.95, 35.59, 32.07, 29.84, 29.80, 29.74, 29.71, 29.68, 29.50, 25.99, 22.83, 14.26. ESI-MS Calcd. for $C_{20}H_{42}N_2O$ $[M]^+$, 326.3297 found 326.3303 m/z.

Preparation of *N,N*-dimethyloctadecane-1,2-diamine (5): Compound **4** (10 g, 30.6 mmol) was

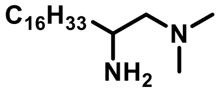

mixed with anhydrous diethyl ether (150 mL). Lithium aluminum hydride ($LiAlH_4$, 3.5 g, 91.8 mmol) was added portion-wise to the reaction mixture at ~0 °C. The reaction was stirred for 8 h and was monitored using TLC. After completion, the reaction mixture was diluted with diethyl ether (100 mL) and quenched with cold water and then by 15% NaOH solution (*Caution:* exothermic and gas evolution!). The obtained emulsion was extracted with diethyl ether (500 mL × 3) and water. The combined organic layer was dried over $MgSO_4$ and concentrated *in vacuo* to obtain a viscous light yellow oily crude product at room temperature which solidified upon refrigeration. This product is used in the next reaction without further purification. Yield: 7.6 g, 79%. ESI-MS Calcd. for $C_{20}H_{44}N_2$ $[M]^+$, 312.3504 found 312.3502 m/z.

Preparation of 12-Oxooctadecanoic acid (6): 12-Hydroxystearic acid (6 g, 20 mmol) was mixed

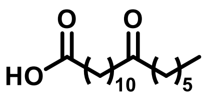

with 10 mL dimethyl sulfoxide and $Na_2Cr_2O_7$ (3.6 g, 14 mmol). In the reaction mixture, conc. H_2SO_4 (2.67 mL, 50 mmol) was added dropwise (*Caution:* exothermic reaction) and stirred at 75 °C for 2 hours and an additional 12 h at room temperature. The reaction mixture was poured into ice-cold water and stirred for 30 minutes. The reaction mixture was extracted with CH_2Cl_2 and water (5 × 500 mL), and the organic layer was dried over Na_2SO_4 and concentrated *in vacuo* to obtain a green-colored compound. The crude product was purified by silica gel chromatography using CH_2Cl_2:hexanes (1:5). Yield: 4.3 g, 72%. ^1H NMR (400 MHz, $CDCl_3$) δ 2.41 – 2.33 (m, J = 14.9, 7.5 Hz, 6H), 1.67 – 1.53 (m, 6H), 1.38 – 1.21 (m, 18H), 0.93 – 0.82 (m, 3H). ^{13}C NMR (101 MHz, $CDCl_3$) δ 212.02, 179.57, 42.98, 42.95, 34.08, 31.76, 29.50, 29.47, 29.38, 29.32, 29.16, 29.09, 24.81, 24.01, 22.64, 14.18. ESI-MS Calcd. for $C_{18}H_{34}O_3$ $[M]^+$, 298.2508 found 298.2487 m/z. Spectra consistent with literature reports.[18]

Preparation of *N,N*-Dimethyl-12-oxooctadecanamide (7): 6 (2 g, 6.7 mmol), dimethylamine hydrochloride (0.6 g, 7.4 mmol), hexafluorophosphate azabenzotriazole tetramethyl uronium (HATU, 2.8 g, 7.4 mmol) and diisopropylethylamine (DIPEA, 5.8 mL, 33.5 mmol) were dissolved in anhydrous DMF (20 mL) and stirred for 12 h. The reaction mixture was extracted with ethyl acetate and water (250 mL × 4), dried over Na_2SO_4, and concentrated *in vacuo.* The crude product was purified by silica gel chromatography using hexanes:ethyl acetate (2:1). Yield: 1.7 g, 78%. ^{1}H NMR (400 MHz, $CDCl_3$) δ 3.00 (s, 3H), 2.94 (s, 3H), 2.38 (td, *J* = 7.5, 1.8 Hz, 4H), 2.32 – 2.27 (m, 2H), 1.66 – 1.49 (m, 6H), 1.33 – 1.24 (m, 18H), 0.90 – 0.85 (m, 3H). ^{13}C NMR (101 MHz, CDCl3) δ 211.88, 173.39, 42.95, 42.94, 37.43, 35.47, 33.54, 31.74, 29.62, 29.56, 29.54, 29.51, 29.37, 29.07, 25.31, 24.00, 23.98, 22.62, 14.16. ESI-MS Calcd. for $C_{20}H_{39}NO_2$ $[M]^+$, 325.2981 found 325.2992 m/z.

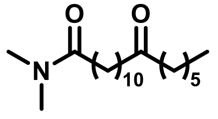

Preparation of 12-Amino-*N,N*-dimethyloctadecanamide (8): 7 (1.7 g, 5.2 mmol) and ammonium acetate (2.4 g, 31.1 mmol) were dissolved in dry methanol (10 mL). Sodium cyanoborohydride ($NaCNBH_3$, 867 mg, 13.8 mmol) was added into reaction mixture and stirred for 36 hours. The reaction was monitored using TLC, and after completion, the product was concentrated *in vacuo*. The residue was dissolved in CH_2Cl_2 and extracted with water (250 mL × 2). The crude product was purified by silica gel chromatography using CH_2Cl_2:CH_3OH (20:1). Yield: 1.6 g, 94%. Data are consistent with literature-reported precedent.[19] ^{1}H NMR (400 MHz, $CDCl_3$) δ 8.36 – 8.22 (m, 2H), 3.13 (m, 1H), 2.99 (s, 3H), 2.92 (s, 3H), 2.32 – 2.24 (m, 2H), 1.75 – 1.54 (m, 6H), 1.50 – 1.22 (m, 22H), 0.90 – 0.81 (m, 3H). ^{13}C NMR (101 MHz, $CDCl_3$) δ 173.36, 52.70, 37.41, 35.46, 33.53, 32.76, 32.72, 31.64, 29.67, 29.63, 29.59, 29.57, 29.48, 29.43, 29.09, 25.46, 25.36, 25.31, 22.68, 14.15. ESI-MS Calcd. for $C_{20}H_{42}N_2O$ $[M]^+$, 326.3297 found 326.3317 m/z.

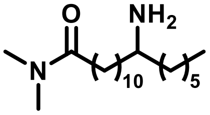

Preparation of *N,N*-Dimethyloctadecane-1,12-diamine (9): 8 (1.5 g, 4.6 mmol) was dissolved in anhydrous diethyl ether (40 mL). Lithium aluminum hydride ($LiAlH_4$, 0.52 g, 13.8 mmol) was added portion-wise to the reaction mixture at ~0 °C. The reaction mixture was stirred for 5 h and was monitored using TLC. After completion, the reaction mixture was diluted with diethyl ether (100 mL) and quenched with cold water and then by 15% NaOH solution (*Caution:* exothermic and gas evolution!). The obtained emulsion was extracted with diethyl ether (500 mL × 2) and water. The combined organic layer was dried over $MgSO_4$ and concentrated *in vacuo.* The compound was purified by silica gel chromatography using CH_2Cl_2:CH_3OH (20:1) to obtain a viscous light yellow oily product at room temperature. Yield: 0.97 g, 68%. ^{1}H NMR (400 MHz, $CDCl_3$) δ 2.77 – 2.53 (m, 1H), 2.20 (s, 8H), 1.46 – 1.17 (m, 30H), 0.87 (t, *J* = 6.6 Hz, 3H). ^{13}C NMR (101 MHz, $CDCl_3$) δ 60.10, 51.33, 45.64, 38.31, 32.00, 29.94, 29.77, 29.74, 29.72, 29.61, 27.91, 27.63, 26.30, 26.27, 22.75, 14.20. ESI-MS Calcd. for $C_{20}H_{44}N_2$ $[M]^+$, 312.3504 found 312.3509 m/z.

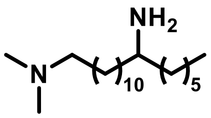

Preparation of *N*-(2-(dimethylamino)ethyl)ferrocenamide (10): Ferrocenecarboxylic acid (300 mg, 1.3 mmol), 2-(dimethylamino)ethylamine (172 mg, 0.2 mL, 1.9 mmol), HATU (544 mg, 1.43 mmol) and diisopropylethylamine (DIPEA, 1.1 mL, 6.5 mmol) were dissolved in anhydrous DMF (10 mL) and stirred for 12 h. The reaction mixture was extracted with ethyl acetate and water (150 mL × 2), dried over Na_2SO_4, and concentrated *in vacuo.* The crude product was purified by silica gel chromatography using $CHCl_3$:CH_3OH (10:1). Yield: 270 mg, 69%. ^1H NMR (400 MHz, DMSO-d_6) δ 7.65 (t, J = 5.9 Hz, 1H), 4.75 (s, 2H), 4.31 (s, 2H), 4.15 (s, 5H), 3.25 (q, J = 6.5 Hz, 2H), 2.36 (t, J = 6.8 Hz, 2H), 2.18 (s, 6H). ^{13}C NMR (101 MHz, DMSO-d_6) δ 168.54, 76.50, 69.58, 69.13, 67.86, 58.27, 45.01, 36.56. ESI-MS Calcd. for $C_{15}H_{20}FeN_2O$ $[M + H]^+$, 301.1003 found 301.0995 m/z.

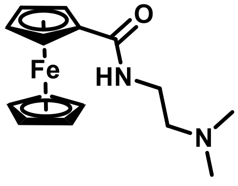

Preparation of *N*-(1-(dimethylamino)octadecan-2-yl)ferrocenamide (11): Compound **5** (1 g, 3.2 mmol), ferrocenecarboxylic acid (613 mg, 2.66 mmol), HATU (1.1 g, 2.9 mmol) and diisopropylethylamine (DIPEA, 2.3 mL, 13.3 mmol) were dissolved in anhydrous DMF (15 mL) and stirred for 6 h. The reaction was monitored by TLC, carefully to avoid overrunning which may cause decomposition of the compound. The reaction mixture was extracted with ethyl acetate and water (150 mL × 2), dried over Na_2SO_4, and concentrated *in vacuo.* This crude product was purified by silica gel chromatography using $CHCl_3$/CH_3OH (20:1). Yield: 760 mg, 54%. ^1H NMR (400 MHz, DMSO-d_6) δ 7.57 (s, 1H), 4.82 (d, J = 37.9 Hz, 2H), 4.39 (s, 2H), 4.17 (s, 6H), 2.76 (d, J = 100.9 Hz, 8H), 1.58 – 1.44 (m, 2H), 1.36 – 1.14 (m, 28H), 0.85 (t, J = 6.6 Hz, 3H). ^{13}C NMR (101 MHz, DMSO-d_6) δ 169.44, 76.55, 70.00, 69.88, 69.48, 69.26, 68.99, 67.69, 44.80, 32.25, 31.28, 29.02, 28.98, 28.92, 28.84, 28.68, 25.33, 22.08, 13.95. ESI-MS Calcd. for $C_{31}H_{52}FeN_2O$ $[M + H]^+$, 525.3507 found 525.3507 m/z.

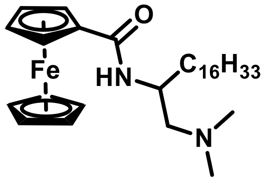

Preparation of *N*-(1-(dimethylamino)octadecan-12-yl)ferrocenamide (12): Ferrocenecarboxylic acid (535 mg, 2.3 mmol), **9** (800 mg, 2.5 mmol), HATU (973 mg, 2.5 mmol) and diisopropylethylamine (DIPEA, 2 mL, 11.6 mmol) were dissolved in anhydrous DMF (25 mL). The reaction was stirred for 6 h and was monitored by TLC. The reaction mixture was extracted with ethyl acetate and water (250 mL × 4), dried over Na_2SO_4, and concentrated *in vacuo.* This crude product was purified by silica gel chromatography using $CHCl_3$:CH_3OH (20:1). Yield: 684 mg, 51%. ^1H NMR (400 MHz, $CDCl_3$) δ 5.44 (d, J = 9.2 Hz, 1H), 4.67 (d, J = 2.0 Hz, 2H), 4.32 (t, J = 1.9 Hz, 2H), 4.20 (s, 5H), 4.10 – 4.00 (m, 1H), 2.24 (s, 8H), 1.50 – 1.17 (m, 30H), 0.92 – 0.82 (m, 3H). ^{13}C NMR (101 MHz, $CDCl_3$) δ 169.54, 70.27, 69.74, 68.09, 59.93, 49.23, 45.44, 35.60, 31.90, 29.75, 29.69, 29.67, 29.64, 29.43, 27.64, 27.52, 26.14, 26.10, 22.71, 14.17. ESI-MS Calcd. for $C_{31}H_{52}FeN_2O$ $[M + H]^+$, 525.3507 found 525.3505 m/z.

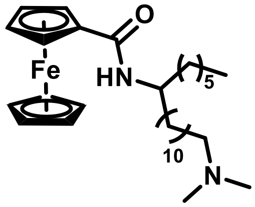

Preparation of *N*-(2-(trimethylaminium iodide)ethyl)ferrocenamide (13): Compound **10** (350 mg, 1.16 mmol) was dissolved in 9 mL anhydrous CH_2Cl_2. 1.5 mL CH_3I was added under N_2, and an orange suspension formed in 30 minutes. The reaction mixture was stirred for an additional 24 h. Excess CH_3I was evaporated *in vacuo.* The crude compound was purified by silica gel chromatography using $CHCl_3$:CH_3OH (20:1), resulting in a hygroscopic brown compound. Yield: 370 mg, 72%. ^{1}H NMR (400 MHz, DMSO-d_6) δ 8.08 (t, J = 5.8 Hz, 1H), 4.77 (d, J = 1.9 Hz, 2H), 4.39 (t, J = 1.9 Hz, 2H), 4.19 (s, 5H), 3.59 (q, J = 6.5 Hz, 2H), 3.46 (t, J = 6.7 Hz, 2H), 3.16 (s, 9H). ^{13}C NMR (101 MHz, DMSO-d_6) δ 169.63, 75.67, 70.15, 69.31, 68.08, 63.67, 52.59, 52.55, 52.51, 33.24. ESI-MS Calcd. for $C_{16}H_{23}FeN_2O^+$ $[M]^+$, 315.1159 found 315.1159 m/z.

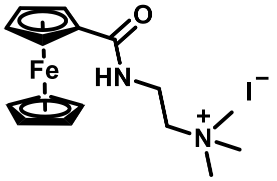

Preparation of *N*-(1-(trimethylaminium iodide)octadecan-2-yl)ferrocenamide (14): Compound **11** (600 mg, 1.14 mmol) was dissolved in 15 mL anhydrous CH_2Cl_2. 3 mL CH_3I was added under N_2, and the reaction mixture was stirred for 24 h. Excess CH_3I evaporated was *in vacuo*. The crude compound was purified by silica gel chromatography using $CHCl_3$:CH_3OH (20:1), resulting in a hygroscopic brown compound. Yield: 640 mg, 84%. ^{1}H NMR (400 MHz, $CDCl_3$) δ 7.30 (br, 1H), 5.10 (q, J = 1.9 Hz, 1H), 4.93 (q, J = 1.8 Hz, 1H), 4.68 (m, 2H), 4.38 (t, J = 2.0 Hz, 2H), 4.23 (s, 5H), 3.47 (m, 1H), 3.32 (s, 9H), 1.74 (m, 2H), 1.40 – 1.10 (m, 28H), 0.88 (t, J = 6.8 Hz, 3H). ^{13}C NMR (101 MHz, $CDCl_3$) δ 171.76, 75.09, 71.16, 71.06, 69.82, 69.27, 69.23, 68.89, 54.65, 45.27, 34.34, 32.05, 29.83, 29.81, 29.79, 29.76, 29.70, 29.49, 29.47, 26.00, 22.82, 14.25. ESI-MS Calcd. for $C_{32}H_{55}FeN_2O^+$ $[M]^+$, 539.3659 found 539.3659 m/z.

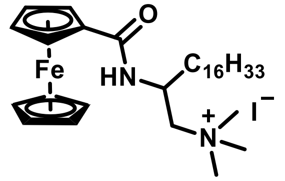

Preparation of *N*-(1-(trimethylaminium iodide)octadecan-12-yl)ferrocenamide (15): Compound **12** (600 mg, 1.14 mmol) was dissolved in 10 mL anhydrous CH_2Cl_2. 3 mL CH_3I was added under N_2, and the reaction mixture was stirred for 36 h. Excess CH_3I evaporated was *in vacuo.* The crude compound was purified by silica gel chromatography using $CHCl_3$:CH_3OH (10:1), resulting in a brown hygroscopic brown compound. Yield: 650 mg, 85%. ^{1}H NMR (400 MHz, $CDCl_3$) δ 5.42 (d, J = 9.4 Hz, 1H), 4.67 (t, J = 1.9 Hz, 2H), 4.34 (t, J = 1.9 Hz, 2H), 4.20 (s, 5H), 4.09 – 3.99 (m, 1H), 3.62 – 3.54 (m, 2H), 3.44 (s, 9H), 1.74 (t, J = 8.2 Hz, 2H), 1.50 – 1.07 (m, 28H), 0.91 – 0.83 (m, 3H). ^{13}C NMR (101 MHz, CDCl3) δ 169.65, 70.39, 69.81, 68.16, 67.42, 53.89, 49.30, 35.70, 35.66, 31.95, 29.67, 29.60, 29.47, 29.37, 29.30, 29.17, 26.17, 26.10, 23.27, 22.76, 14.22. ESI-MS Calcd. for $C_{32}H_{55}FeN_2O^+$ $[M]^+$, 539.3659 found 539.3635 m/z.

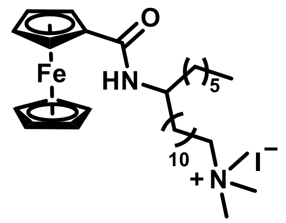

Preparation of *N*-(2-(trimethylaminium chloride)ethyl)ferrocenamide (16): Compound **13** (200 mg, 0.45 mmol) was taken in a mixture of $CH_2Cl_2/CH_3OH/H_2O$ (4/2/1 mL) and AgCl (322 mg, 2.25 mmol) was added. The reaction was stirred for 24 h and monitored using TLC. After completion, the mixture was diluted with CH_2Cl_2 (100 mL), filtered through celite and Na_2SO_4, and concentrated *in vacuo* to obtain a solid brown compound. Yield: 155 mg, 97%. ^{1}H NMR (400 MHz, DMSO-d_6) δ 8.37 (br, 1H), 4.82 (dd, J = 4.6, 2.0 Hz, 2H), 4.38 (t, J = 2.0 Hz, 2H), 4.19 (s, 5H), 3.60 (q, J = 6.3 Hz, 2H), 3.49 (dd, J = 6.7, 3.5 Hz, 2H), 3.16 (s, 9H). ^{13}C NMR (101 MHz, DMSO-d_6) δ 169.67, 75.87, 75.84, 70.16, 69.35, 68.24, 68.21, 63.80, 52.64, 52.60, 52.57, 33.39. ESI-MS Calcd. for $C_{16}H_{23}FeN_2O^+$ $[M]^+$, 315.1154 found 315.1154 m/z.

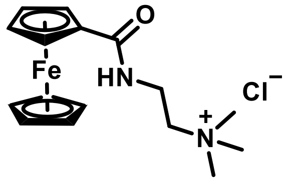

Preparation of *N*-(1-(trimethylaminium chloride)octadecan-2-yl)ferrocenamide (17): Compound **14** (500 mg, 0.75 mmol) was taken in a mixture of $CH_2Cl_2/CH_3OH/H_2O$ (10/4/2 mL) and AgCl (537 mg, 3.75 mmol) was added. The reaction was stirred for 24 h and monitored using TLC. After completion, the mixture diluted with CH_2Cl_2 (150 mL), filtered through celite and Na_2SO_4, and concentrated *in vacuo* to obtain a solid brown compound obtained. Yield: 392 mg, 91%. ^{1}H NMR (500 MHz, DMSO-d_6) δ 8.05 (s, 1H), 4.98 – 4.74 (m, 2H), 4.57 – 3.98 (m, 7H), 3.62 – 3.44 (m, 2H), 3.11 (s, 9H), 1.69 – 1.04 (m, 30H), 0.85 (t, 3H). ^{13}C NMR (126 MHz, DMSO-d_6) δ 169.22, 76.27, 70.16, 70.11, 69.17, 68.58, 68.51, 68.15, 52.99, 44.27, 33.48, 31.27, 29.02, 28.99, 28.92, 28.71, 28.69, 25.01, 22.08, 13.95. ESI-MS Calcd. for $C_{32}H_{55}FeN_2O^+$ $[M]^+$, 539.3658 found 539.3653 m/z.

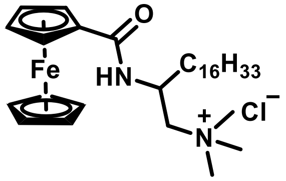

Preparation of *N*-(1-(trimethylaminium chloride)octadecan-12-yl)ferrocenamide (18): Compound **15** (600 mg, 0.9 mmol) was taken in a mixture of $CH_2Cl_2/CH_3OH/H_2O$ (12/4/2 mL) and AgCl (650 mg, 4.53 mmol) was added. The reaction was stirred for 36 h and monitored using TLC. After completion, the mixture was diluted with CH_2Cl_2 (150 mL), filtered through celite and Na_2SO_4, and concentrated *in vacuo* to obtain a solid brown compound. Yield: 490 mg, 95%. ^{1}H NMR (400 MHz, DMSO-d_6) δ 7.32 (d, J = 8.9 Hz, 1H), 4.83 (t, J = 1.9 Hz, 2H), 4.32 (t, J = 1.9 Hz, 2H), 4.14 (s, 5H), 3.86 (q, J = 7.3 Hz, 1H), 3.28 – 3.20 (m, 2H), 3.02 (s, 9H), 1.63 (t, J = 8.2 Hz, 2H), 1.46 – 1.40 (m, 3H), 1.24 (s, 25H), 0.87 – 0.79 (m, 3H). ^{13}C NMR (101 MHz, DMSO-d_6) δ 168.24, 77.24, 69.68, 69.15, 68.17, 65.23, 52.12, 52.08, 52.05, 47.98, 34.70, 31.31, 29.07, 28.99, 28.93, 28.89, 28.75, 28.69, 28.45, 25.76, 25.74, 22.06, 21.99, 13.94. ESI-MS Calcd. for $C_{32}H_{55}FeN_2O^+$ $[M]^+$, 539.3658 found 539.3661 m/z.

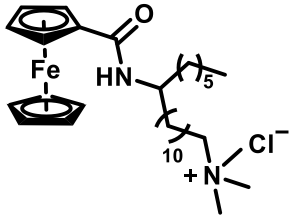

Supplemental Tables

Compounds	CMC
	in water (μM)
**C18-Fc**	135±06
**C18(C12)-Fc**	220±32
CTAB	1069±44

Table S1. Critical micelle concentrations (CMCs) of compounds in ultrapure water determined by dynamic light scattering.

**C18-Fc** Concentration (μM)	Density (g/cm^3), Surface Tension (mN/m)	
	Ultrapure water	0.1 M $NaClO_4$
200	0.99709, 42.47±0.51	1.00477, 35.04±5.13

Table S2. Density and surface tension of **C18-Fc** in ultrapure water and 0.1 M $NaClO_4$.

Probe	E_{pa} in MeCN	E_{pc} in MeCN	$E_{1/2}$ in MeCN	ΔE_p in MeCN
**C2-Fc**	0.234 V	0.175 V	0.205 V	59 mV
**C18-Fc**	0.245 V	0.185 V	0.215 V	60 mV
**C18(C12)-Fc**	0.232 V	0.170 V	0.205 V	62 mV

Table S3. Peak potentials (anodic and cathodic), redox potentials, and peak potential separation for **C2-Fc**, **C18-Fc**, and **C18(C12)-Fc** in 0.1 M $TBAClO_4$ in acetonitrile. All potentials quoted vs Fc/Fc^+. Data are taken from **Figure 1a.**

Probe	$E_{1/2}$ in H_2O (**Figure 1b**)	$E_{1/2}$ in H_2O (**Figure S8**)
**C2-Fc**	0.445 V	0.445 V
**C18-Fc**	0.563 V	0.563 V
**C18(C12)-Fc**	0.512 V	0.506 V

Table S4. Redox potentials ($E_{1/2}$) for **C2-Fc**, **C18-Fc**, and **C18(C12)-Fc** in 0.1 M $NaClO_4$ in H_2O. All potentials quoted vs Ag/AgCl. Data shown for two experiments repeated on independent preparations of the electrochemical cell to demonstrate reproducibility in the peak shifts observed.

Probe	E_{pa} in H_2O	E_{pc} in H_2O	$E_{1/2}$ in H_2O	ΔE_p in H_2O
**C2-Fc**	0.472 V	0.418 V	0.445 V	54 mV
**C18-Fc**	0.566 V	0.559 V	0.563 V	7 mV
**C18(C12)-Fc**	0.499 V	0.525 V	0.512 V	26 mV

Table S5. Peak currents (anodic and cathodic), redox potentials, and peak potential separation for **C2-Fc**, **C18-Fc**, and **C18(C12)-Fc** in 0.1 M $NaClO_4$ in H_2O. All potentials quoted vs Ag/AgCl. Data are taken from **Figure 1b.**

Atom	x	y	Z
Fe	-0.2430757	0.60700938	-0.4551726
C	-2.0267574	1.43590505	-1.0344565
C	-1.053901	2.45758404	-0.817461
C	-1.5307329	0.55977651	-2.0435773
H	-2.9697844	1.32647233	-0.5003324
C	0.04341097	2.21553563	-1.6963266
H	-1.1271635	3.2684974	-0.0936754
C	-0.2517116	1.04347309	-2.4552818
H	-2.0177093	-0.3478168	-2.3982913
H	0.95333951	2.81089611	-1.7628079
H	0.39424485	0.58819785	-3.2051527
C	1.13055784	0.61170491	1.07494043
C	-0.1627597	0.26559579	1.57033944
C	1.49139441	-0.3362546	0.07437525
H	1.72845774	1.46707006	1.3873149
C	-0.6133006	-0.8901572	0.87409454
H	-0.7211194	0.81038929	2.33052307
C	0.41194882	-1.2777022	-0.0506418
H	2.40935624	-0.3141322	-0.5131265
H	-1.5628935	-1.407393	0.99781388
C	0.2810956	-2.4479494	-0.9575488
O	-0.803355	-2.909908	-1.284989
N	1.46738517	-2.9860299	-1.3811364
C	1.56821118	-4.21912	-2.1594134
H	2.31415226	-2.6355969	-0.9533705
C	0.92296892	-3.9244483	-3.5288597
N	1.1275048	-4.8858561	-4.6702312
H	1.34091279	-2.9669023	-3.8697137

H	-0.1596429	-3.7917112	-3.4005725
C	0.81135377	-4.1582333	-5.9494913
H	1.50632452	-3.3147312	-6.0512692
H	-0.2216213	-3.7912893	-5.8932737
H	0.95089222	-4.8985837	-6.7710878
C	2.53717603	-5.3814674	-4.7729903
H	2.77137414	-5.996839	-3.896078
H	3.21215946	-4.5166196	-4.8266548
H	2.578771	-5.9954674	-5.7042089
C	0.20586752	-6.0714559	-4.6170164
H	-0.8268947	-5.7040262	-4.565738
H	0.43340153	-6.6731167	-3.7325066
H	0.39700558	-6.6464075	-5.5513418
Cl	1.57485718	-6.9421306	-7.4816627
C	1.02820923	-5.4373446	-1.3941613
H	2.64937465	-4.3653567	-2.3159595
C	1.65200883	-5.6257199	-0.0129818
H	1.22524642	-6.3427368	-1.9891217
H	-0.0652003	-5.3432014	-1.3038558
C	1.10348619	-6.8401133	0.73366642
H	1.47672843	-4.7237166	0.59739377
H	2.75024959	-5.7188671	-0.1096762
C	1.69513123	-7.0190718	2.1299334
H	1.28269552	-7.7541972	0.13852944
H	0.00520972	-6.7463521	0.81245033
C	1.09686658	-8.1869954	2.91066695
H	1.5506983	-6.0867529	2.70646068
H	2.78981087	-7.1525659	2.0502741
C	1.68391282	-8.3575633	4.3100296

H	1.23532453	-9.1223158	2.33792266
H	0.00307134	-8.0468258	2.98952586
C	1.0483194	-9.487062	5.11749437
H	1.57496401	-7.4086374	4.86706613
H	2.77301745	-8.5310823	4.23132069
C	1.62979205	-9.6443749	6.52070286
H	1.15714506	-10.439322	4.56653301
H	-0.0406951	-9.3112281	5.19243846
C	0.97069097	-10.745567	7.34814356
H	1.54103591	-8.6831418	7.05996793
H	2.71504895	-9.8424215	6.44659604
C	1.54943701	-10.890863	8.7537712
H	1.06011905	-11.709386	6.81392545
H	-0.1145688	-10.546549	7.41984686
C	0.87802211	-11.973896	9.59477777
H	1.47189468	-9.921688	9.28040458
H	2.6323296	-11.102545	8.68269677
C	1.45469309	-12.108512	11.0024717
H	0.9577799	-12.945175	9.07258622
H	-0.2051846	-11.76283	9.66310821
C	0.77826768	-13.182169	11.8517697
H	1.38077384	-11.1344	11.5204414
H	2.53675594	-12.325357	10.9342131
C	1.35351777	-13.310382	13.2602756
H	0.85424776	-14.157461	11.3364186
H	-0.3041298	-12.966214	11.91808
C	0.67692996	-14.381427	14.1134438
H	1.27955012	-12.334337	13.7750079
H	2.43588217	-13.527638	13.1945348

C	1.25608307	-14.501149	15.51823
H	0.75183007	-15.356415	13.5988117
H	-0.4045797	-14.163864	14.1774817
H	2.32746551	-14.759589	15.4907024
H	0.74296412	-15.279686	16.1041776
H	1.16274569	-13.553207	16.0732796

Table S6. Coordinates for the geometry-optimized structure of **C18-Fc** obtained using DFT calculations employing a B3PW91 functional with an extended basis set.

Materials	Total Amount[a] (mg/mL)	Moles of Material on Electrode ($\times10^{-4}$, mole/cm^2)[b]	Electroactive material on Electrode ($\times10^{-9}$, mol/cm^2)[c]
**ZnPc**	7.9	1.93	0.45
**CoPc**	8.3	2.05	2.99
**FeOEPCl**	1.3	2.95	7.94

[a]stock solution concentrations in CH_2Cl_2 (0.8 mL), ethanol (0.15 mL) and Nafion perfluorinated resin, 5 wt %, (0.05 mL). [b]moles on electrode calculated from aliquot used for Zn(II)Pc (5 µL), Co(II)Pc (10 µL), and Fe(III)OEPCl (5 µL) from stock concentration of ink, which was dropcast onto glassy carbon electrode. [c]electroactive material calculated by integration of the redox feature associated with ZnPc (II/I), CoPc (II/I) and FeOEPCl (III/II) (**Fig. S26–S28**).

Table S7. Summary of ZnPc, CoPc, and FeOEPCl loading on glassy carbon electrodes.

Peak Assignment	Peak found / cm^{-1}	Peak found / cm^{-1} Potential at 1.1 V *vs* Ag/AgCl	Peak found / cm^{-1} Potential at −1.8 V *vs* Ag/AgCl
$\nu_{sym}(N-CH_3)$[20–24]	2963	2963	2963
ν_{asym} CH_2[21,22]	2925	2925	2925
ν_{sym} CH_2[21,22]	2853	2853	2853
δ(NH) amide	1535	1535	1535
$\delta(CH_2)$ aliphatic tail[23,25–27]	1479	1479	1479

Table S8: Comparison of the IR spectroscopic features for *in-situ* SEIRAS data taken in the presence of **C18-Fc** (200 μM) on Au film at 1.1 V and −1.8 V *vs* Ag/AgCl in 0.1 M $NaClO_4$. Data are reported in **Figure S30**. Peak assignments made based on literature precedent as referenced and described in the main text.

Supplemental Figures

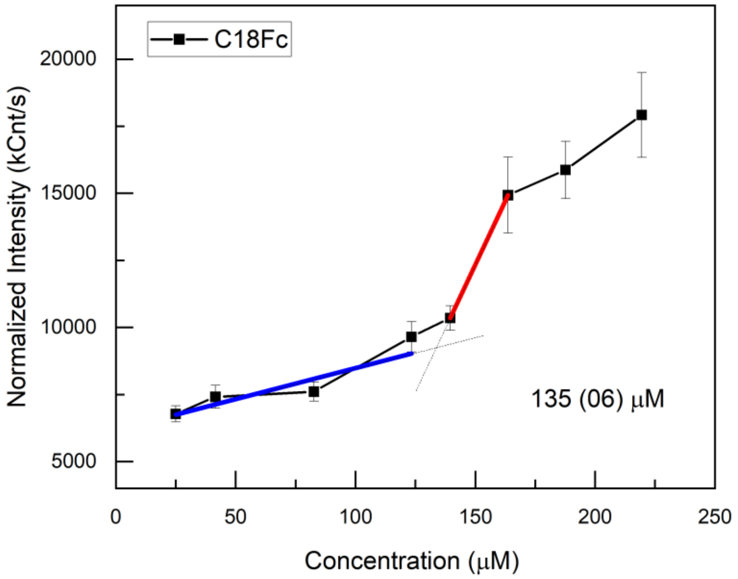

Fig. S1. Plot of the intensity of scattered light (kilo counts per sec, kCnt s^{-1}) obtained with various concentrations of **C18-Fc** in water. Intersection of two lines corresponds to critical micelle concentration.

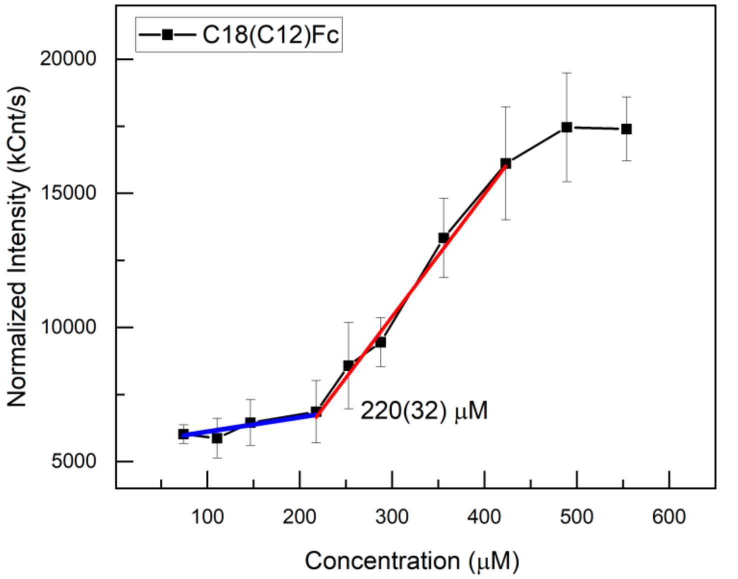

Fig. S2. Plot of the intensity of scattered light (kilo counts per sec, kCnt s^{-1}) obtained with various concentrations of **C18(C12)-Fc** in water. Intersection of two lines corresponds to critical micelle concentration.

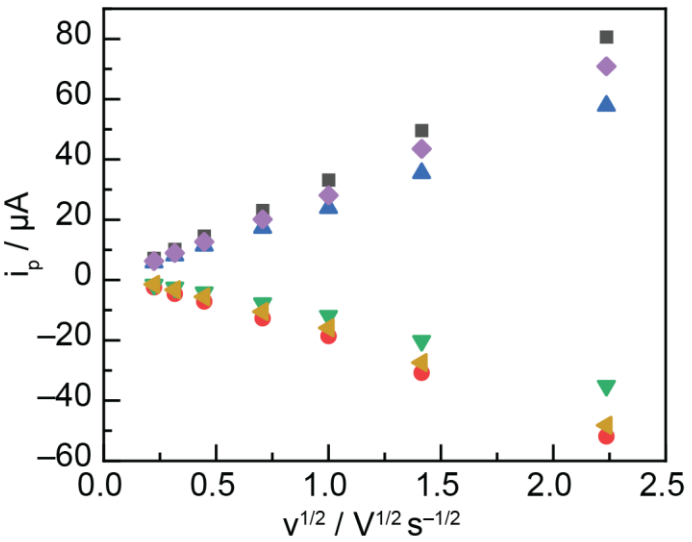

Fig. S3. Peak current of cyclic voltammograms obtained at varying scan rates for 1 mM **C18-Fc** (blue triangles, anodic peak current; green triangles, cathodic peak current), 1 mM **C2-Fc** (black squares, anodic peak current; red circles, cathodic peak current), and 1 mM **C18(C12)-Fc** (purple diamonds, anodic peak current; yellow triangles, cathodic peak current) in 0.1 M $TBAClO_4$ in MeCN, working electrode as Au disk electrode and Ag/AgCl reference electrode. Data used to estimate the diffusion coefficients for the three molecules.

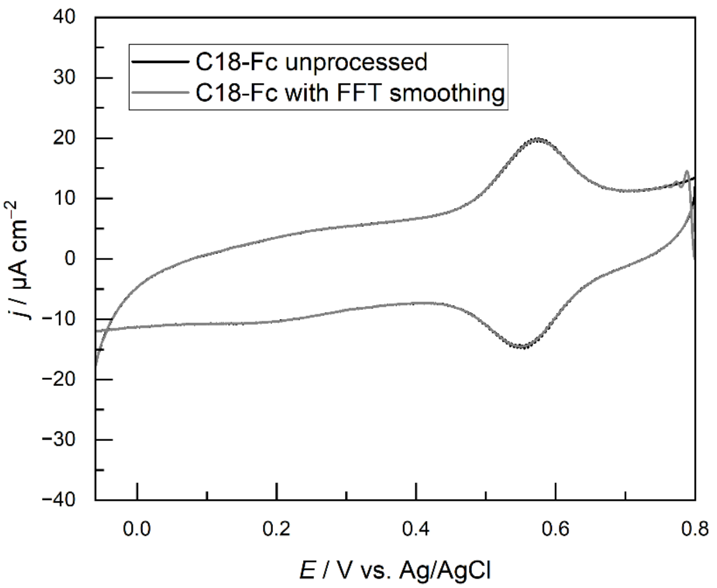

Fig. S4. Cyclic voltammogram of 75 µM C18-Fc in aqueous 0.1 M $NaClO_4$ electrolyte solution without (black) and with (gray) FFT smoothing (point of window = 15). CV was recorded with a working Au disk electrode, Ag/AgCl reference electrode, and at scan rate of 100 mV s^{-1} with a

positive direction of scan. FFT smoothing utilized to improve the readability of CVs utilizing resistance correction (see experimental description in the SI).

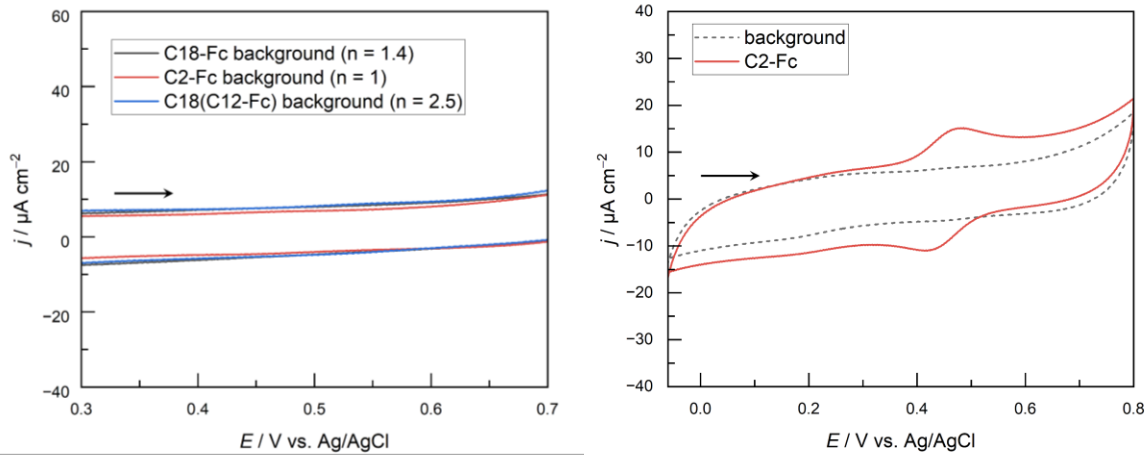

Fig. S5. Cyclic voltammograms of an annealed Au disk electrode in aqueous 0.1 M $NaClO_4$ electrolyte solution before adding **C18-Fc** (black), **C2-Fc** (red), and **C18(C12)-Fc** (blue). Background CVs were scaled to a uniform double-layer capacitance by dividing by normalizing factor (n = 1.4 for **C18-Fc**, 1 for **C2-Fc**, and 2.5 for **C18(C12)-Fc**). (**b**) Cyclic voltammograms of annealed Au disk electrode in aqueous 0.1 M $NaClO_4$ electrolyte solution with only electrolyte (grey dashed) and with 75 µM **C18(C12)-Fc** (red), recorded at 50 mV s^{-1}. Data normalized for the varying background capacitive value reported in **Figure 1b** and as discussed in **Figure S5** (left). Experiments are conducted with a positive direction of scan. We observed that different annealed Au disk electrodes exhibited a variation in the double-layer capacitance for experiments conducted under identical conditions. To account for this variability and to reveal the differences in the relative double layer capacitance for data collected in the presence of **C18-Fc**, **C18(C12)-Fc**, and **C2-Fc**, we normalized the varying background observed for each flame-annealed Au disk electrode utilized (i.e., CVs of the background collected at the same scan rate on the same electrode in the same electrolyte).

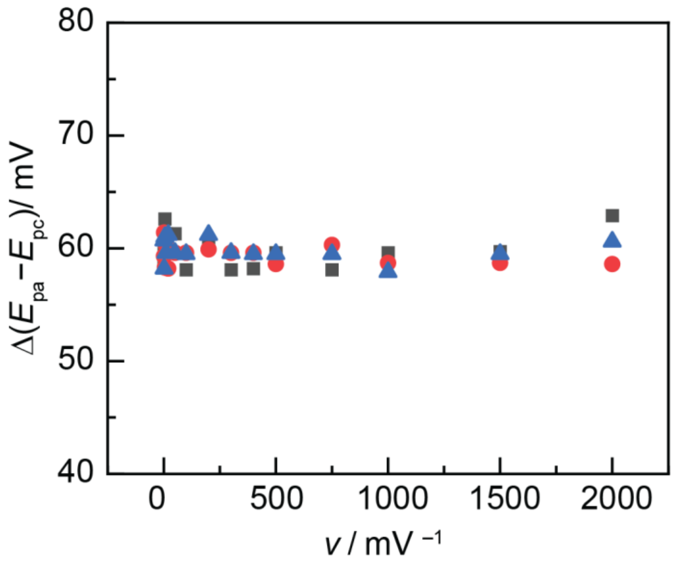

Fig. S6. Differences in the peak potential of **C2-Fc** as a function of the scan rate. Data collected in 0.1 M $NaClO_4$ on a stationary Au disk electrode containing 75 µM (black squares), 135 µM (red circles), or 200 µM (blue triangles) of **C2-Fc**.

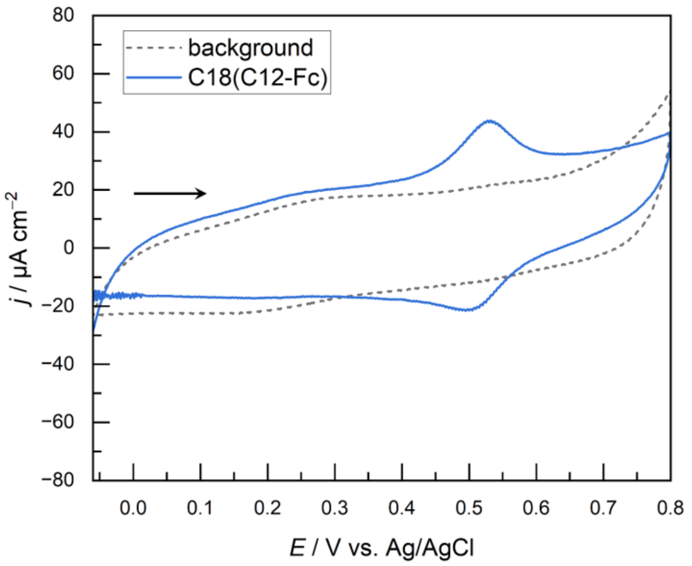

Fig. S7. Cyclic voltammograms of annealed Au disk electrode in aqueous 0.1 M $NaClO_4$ electrolyte solution with only electrolyte (grey dashed) and with 75 µM **C18(C12)-Fc** (blue), recorded at 50 mV s^{-1}. Data normalized for the varying background capacitive value reported in **Figure 1b** and as discussed in **Figure S5** (left). Experiments are conducted with a positive direction of scan.

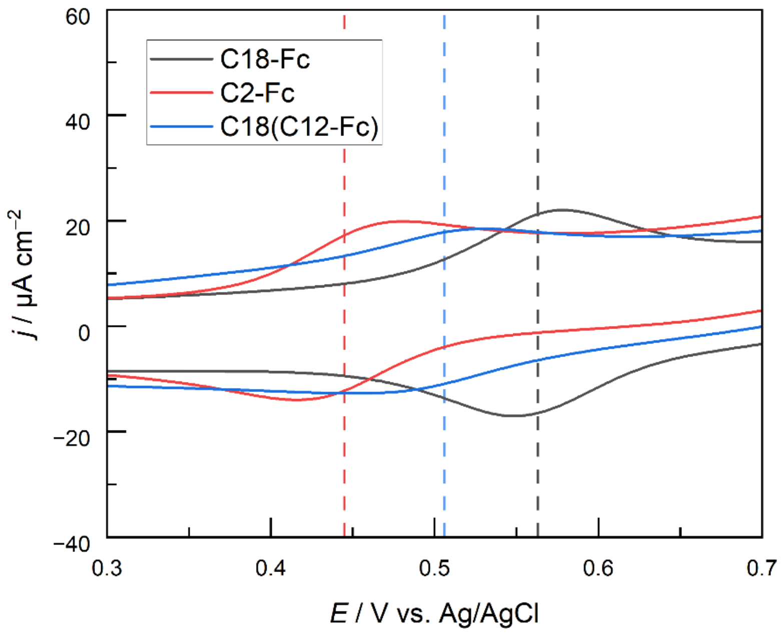

Fig. S8. CV of 75 μM **C2-Fc** (red), 75 μM **C18(C12)-Fc** (blue), or 75 μM **C18-Fc** (black) in 0.1 M $NaClO_4$ in H_2O collected at 100 mV s^{-1} on a Au working disk electrode. Dashed lines estimate the $E_{1/2}$ of the reversible CV waves observed. Data conducted on an independent preparation of the electrochemical cell as that shown in **Figure 1b**.

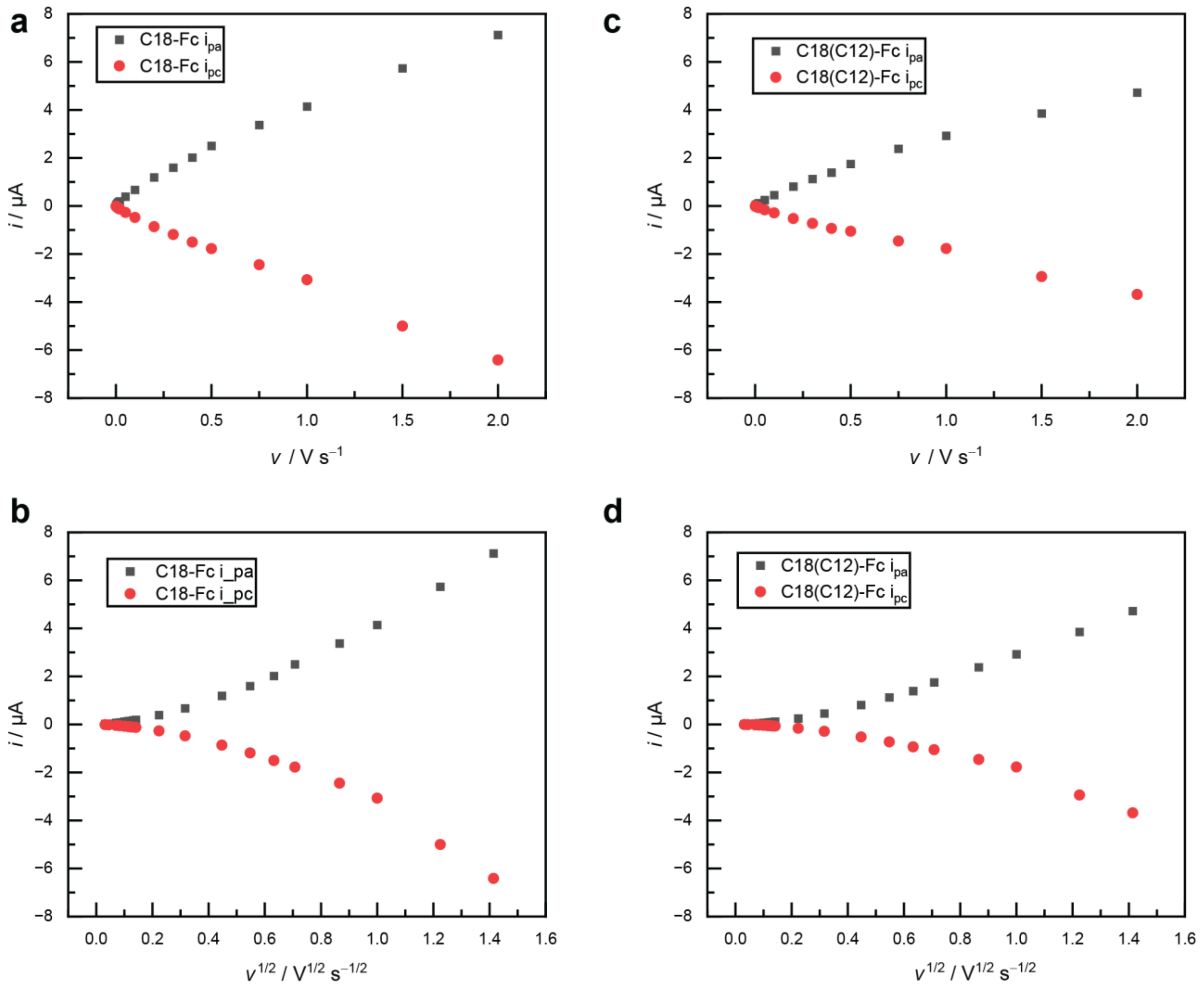

Fig. S9. Peak current for anodic (black) and cathodic (red) sweeps for **(a) C18-Fc** vs. scan rate **(b) C18-Fc** vs. square root of scan rate **(c) C18(C12)-Fc** vs. scan rate and **(d) C18(C12)-Fc** vs. square root of scan rate.

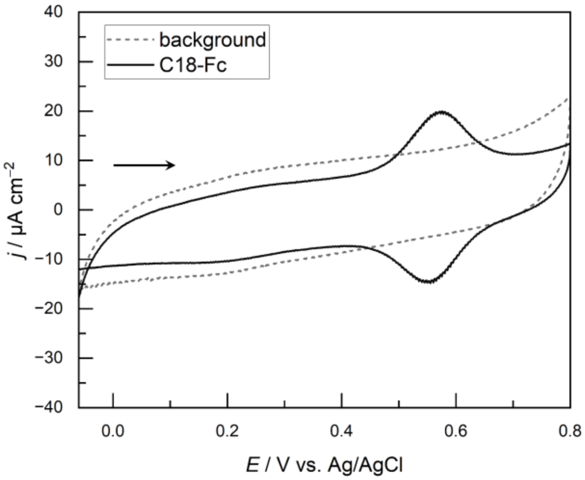

Fig. S10. Cyclic voltammograms of annealed Au disk electrode in aqueous 0.1 M $NaClO_4$ electrolyte solution with only electrolyte (grey dashed) and with 75 µM **C18-Fc** (black), recorded at 50 mV s^{-1}. Experiments are conducted with a positive direction of scan.

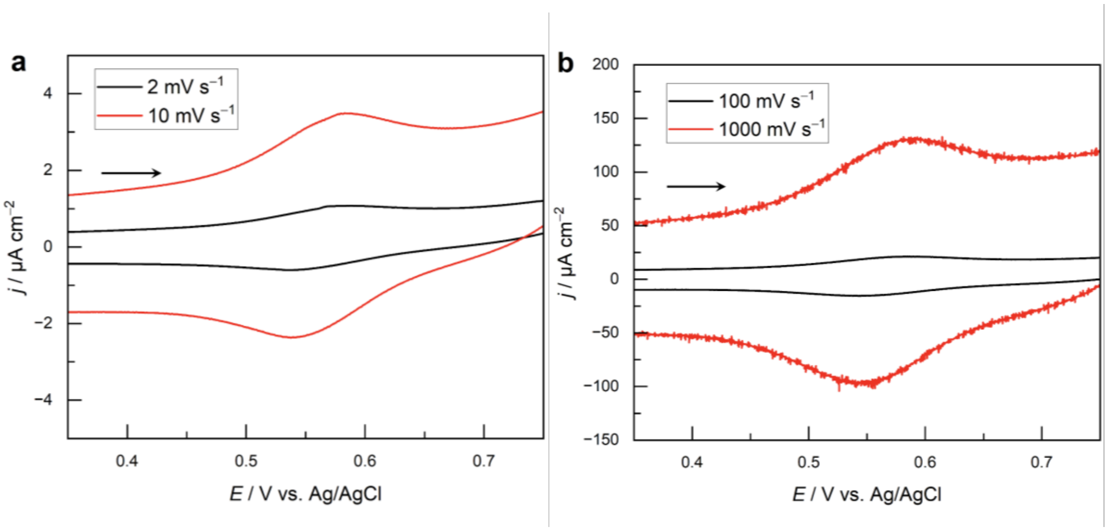

Fig. S11. Cyclic voltammograms of 75 µM **C18-Fc** in aqueous 0.1 M $NaClO_4$ electrolyte solution recorded at scan rates of (**a**) 2 and 10 mV s^{-1} and (**b**) 100 and 1000 mV s^{-1} on an annealed Au electrode. While, in principle, a ΔE_p of zero is expected for a non-interacting, surface-bound redox-active moiety exhibiting fast electron transfer kinetics at the scan rates examined,[28,29] literature reports suggest that the electronic interaction of Au with Fc positioned at short distances to the Au electrode (within one to two methylene units from the S–Au linkage) leads to observed CV peak distortions and separation.[30–40] While these effects convolute the elucidation of a well-defined electron transfer rate to **C18-Fc** from the Au electrode via the Laviron formalism[28,29,41–45] and Marcus Theory,[46] the similarity between the ΔE_p observed in this work and covalently-bound ferrocene to Au suggest that **C18-Fc** are similarly immobilized at the Au surface.

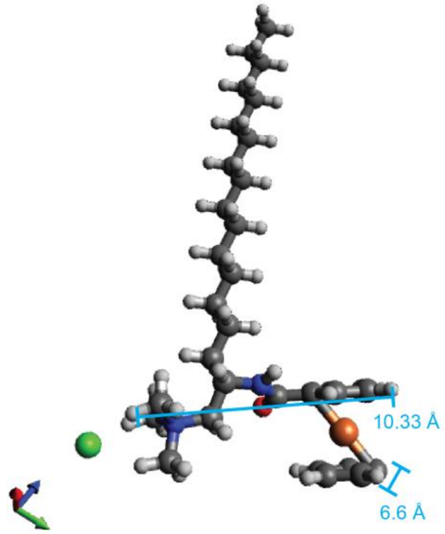

Fig. S12. Geometry-optimized structure of **C18-Fc** using ORCA 5.0.1 with B3PW91 functional with an extended basis set. Coordinates are designated as red = x, green = y, and blue = z. The distances utilized to calculate hypothetical monolayer formation (see above calculation) are also indicated.

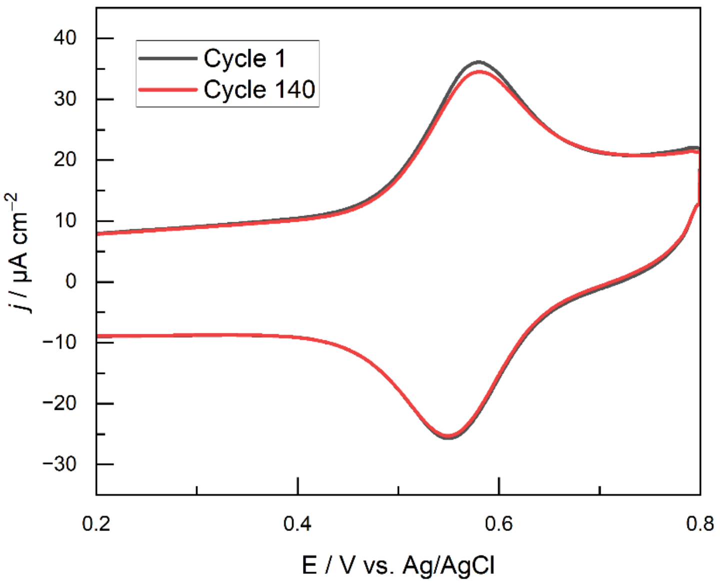

Fig. S13. Cyclic voltammograms of 100 μM C18-Fc in aqueous 0.1 M $NaClO_4$ electrolyte solution, working as Au disk electrode, Ag/AgCl reference electrode, recorded at scan rate of 100 mV s^{-1} with positive direction of scan. First and last of 140 consecutive CV scans are shown (first cycle in black, 140th cycle in red).

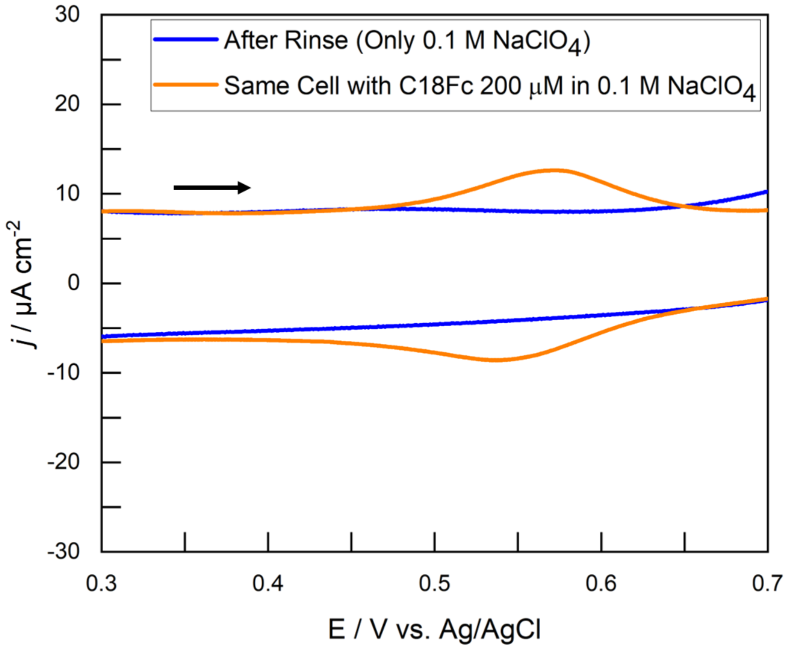

Fig. S14. Cyclic voltammograms of Au disk electrode in aqueous 0.1 M $NaClO_4$ electrolyte solution in blank electrolyte (blue) and after adding 200 μM **C18-Fc** (orange) on an annealed Au disk electrode. Experiments are conducted with a positive direction of scan.

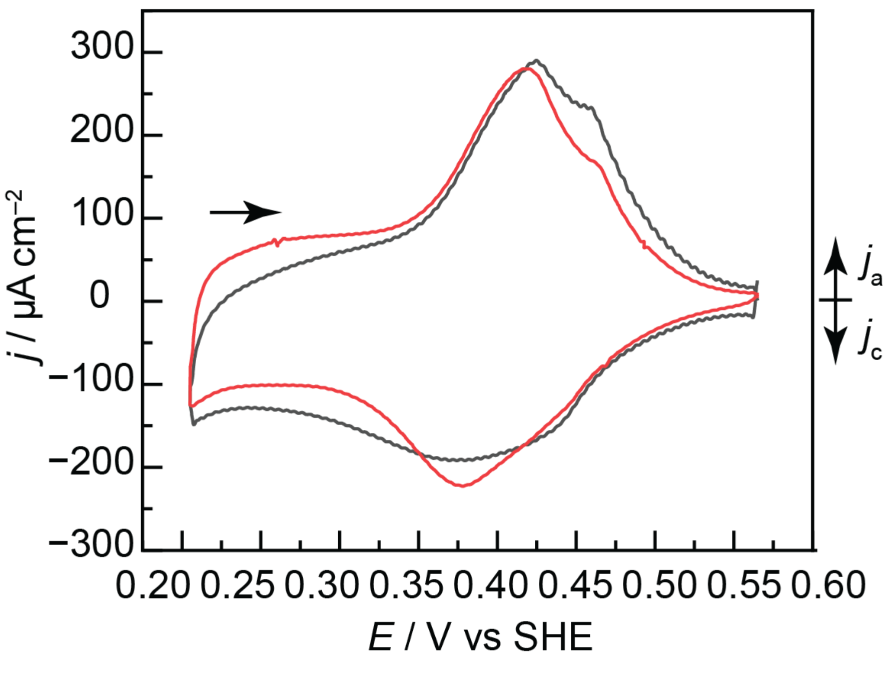

Fig. S15. Cyclic voltammograms of a freshly prepared SEIRAS-active Au film (red) or a freshly polished Au disk electrode (black) in 0.5 M H_2SO_4 containing 7.5 mM $CuSO_4$. Experiments were conducted at 50 mV s^{-1} with a positive direction of scan. The current density is reported relative

to the geometric surface area of each of the Au electrode materials examined. Cu underpotential deposition on Au estimates the electrochemically active surface area.[47,48] We observe that the integrated charges for the Cu UPD feature normalized for the geometric surface area of each Au material (SEIRAS-active Au film versus the Au disk electrode) over the range of 0.34 to 0.50 V vs SHE are roughly similar. These results demonstrate that the integrated charge of 24 µC cm^{-2} reported for the **C18-Fc** wave observed on the SEIRAS-active Au film in **Figure 2c** is representative of the surface area of the nanostructured Au film.

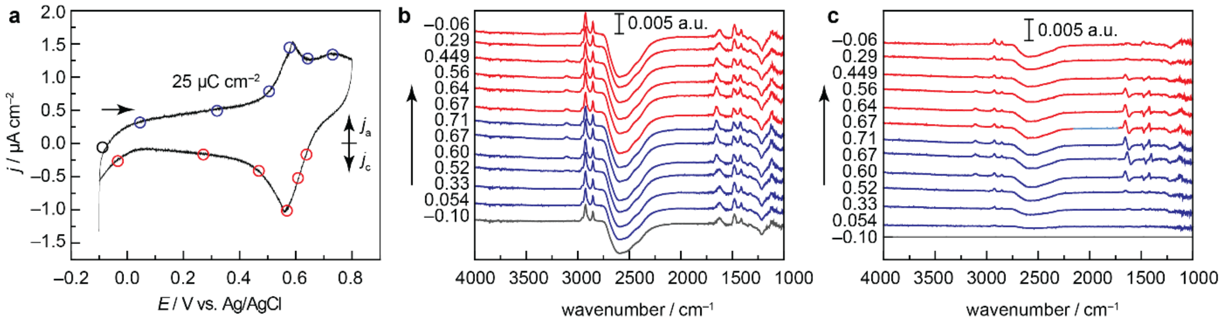

Fig. S16. Cyclic voltammetry (CV) and simultaneously collected SEIRA spectra on a Au film in the presence of 0.1 M $NaClO_4$ in D_2O and 200 µM **C18-Fc**. (**a**) The initial, first CV cycle collected at 2 mV s^{-1} from –0.1 V vs Ag/AgCl. The anodic integrated charge is shown. (**b**) Simultaneously collected SEIRA spectra on the first CV cycle at the potential values indicated, where the background spectrum was collected at –0.1 V vs Ag/AgCl in 0.1 M $NaClO_4$ in the absence of 200 µM **C18-Fc**. (**c**) Identical spectra as shown in b, however, the background spectrum was collected at –0.1 V vs Ag/AgCl in 0.1 M $NaClO_4$ in the presence of 200 µM **C18-Fc**.

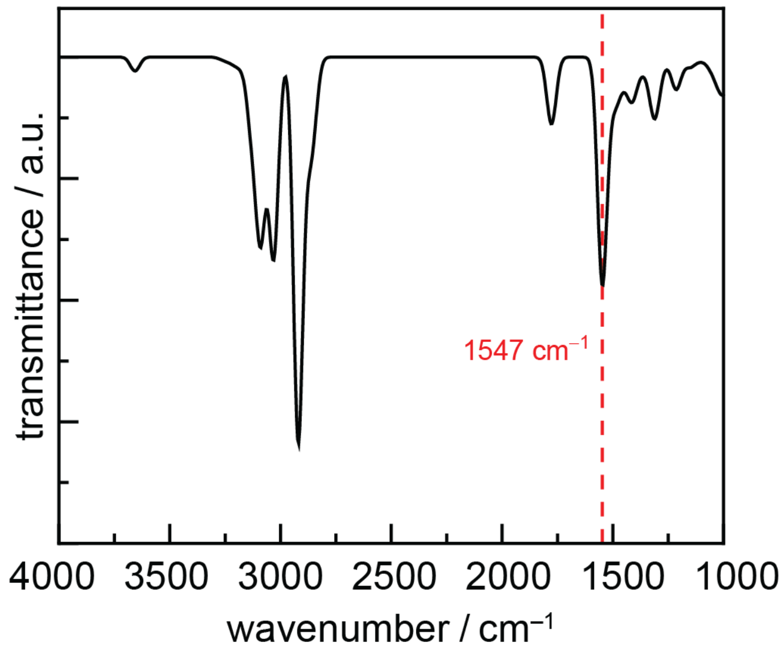

Fig. S17. Computed IR spectrum of **C18-Fc**. The computed normal mode for the δ(NH) of the amide linker is indicated in red.

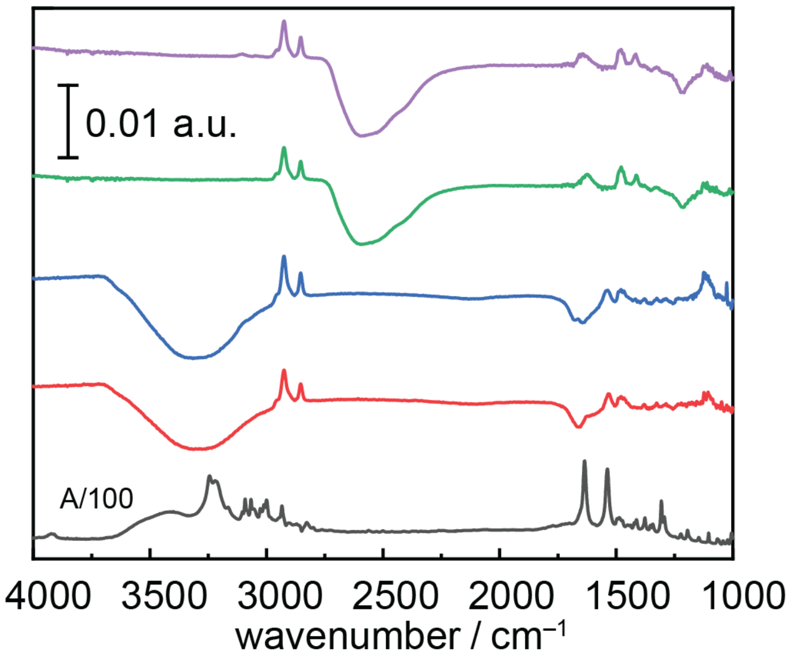

Fig. S18. Comparison of simultaneously collected SEIRA spectra on a Au film in the presence of 0.1 M $NaClO_4$ in D_2O and 200 µM **C18-Fc** versus 0.1 M $NaClO_4$ in H_2O and 200 µM **C18-Fc** and **C2-Fc** bulk IR spectrum. (purple) Identical spectra to **Figure S16b** at 0.60 V in D_2O. (green) Identical spectra to **Figure S16b** at 0.09 V in D_2O. (blue) Identical spectra to **Figure 2b** at 0.60 V in H_2O (first scan). (red) Identical spectra to **Figure 2b** at 0.21 V in H_2O (first scan). (black) Bulk IR spectrum of **C2-Fc** collected with KBr.

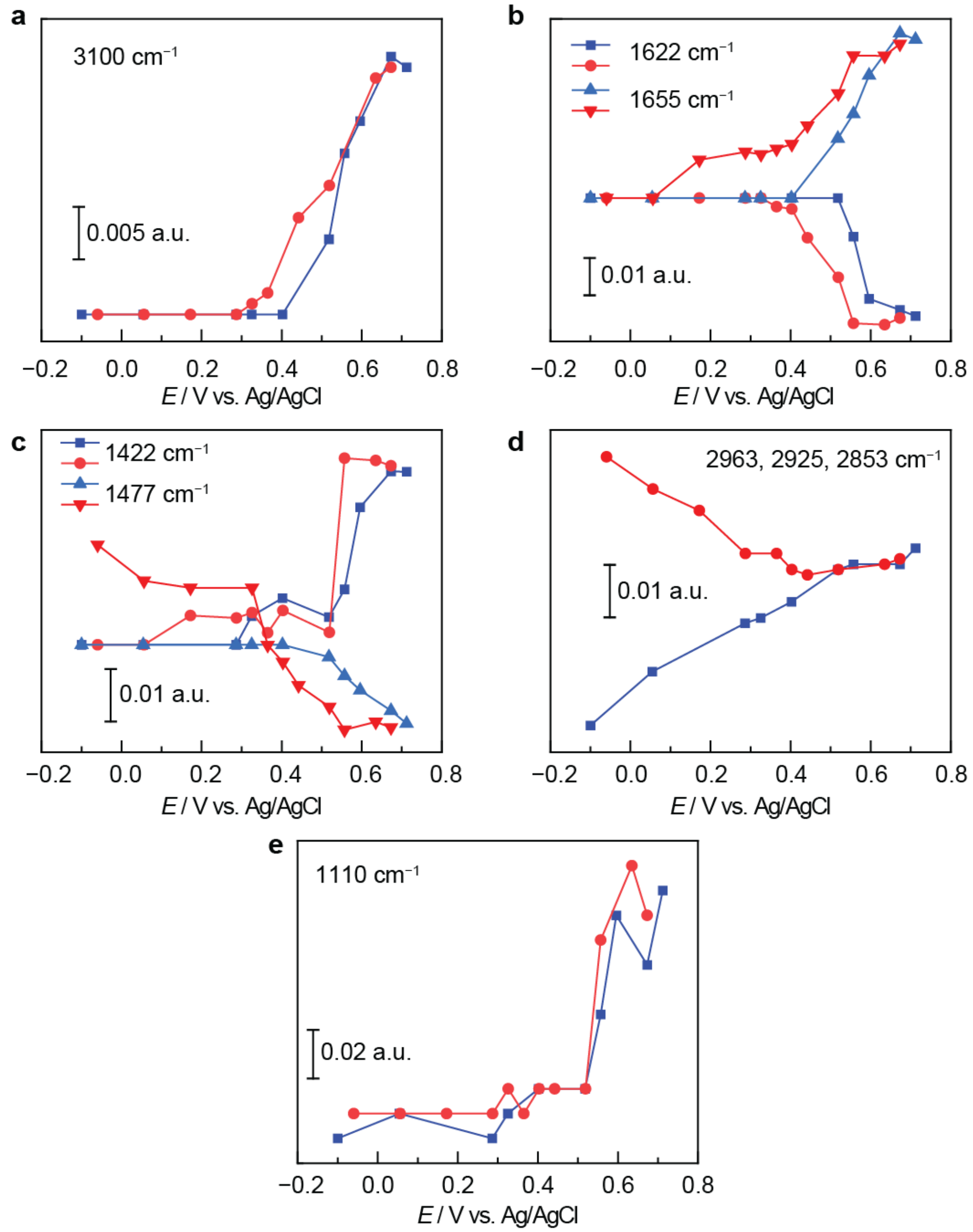

Fig. S19. Integrated band intensities of peak centered at (**a**) 3100; (**b**) 1622 and 1655; (**c**) 1422 and 1477; (**d**) 2963, 2925, 2853; and (**e**) 1110 cm^{-1} from simultaneously collected SEIRA spectra on a Au film in the presence of 0.1 M $NaClO_4$ in D_2O and 200 µM **C18-Fc** shown in **Figure S16**. Blue indicates the integrated band intensities of the forward-going oxidative sweep. Red denotes the integrated band intensities of the reverse-going reductive sweep.

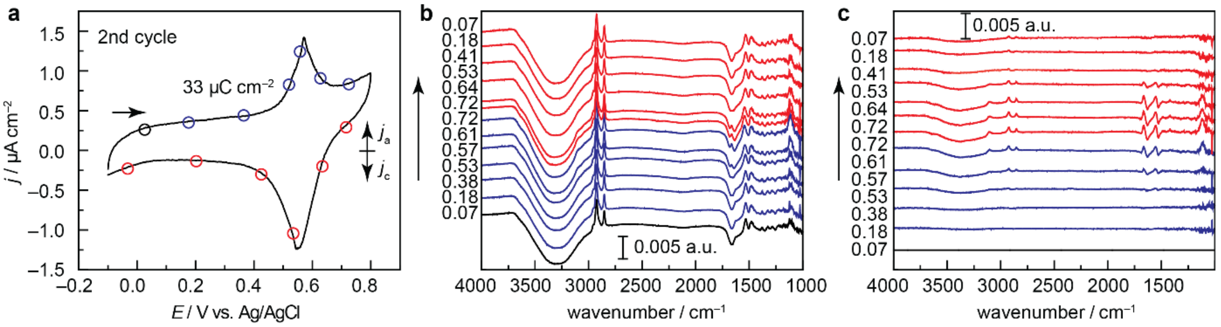

Fig. S20. (**a**) The second CV cycle collected at 2 mV s^{-1} from –0.1 V vs Ag/AgCl immediately following that shown in **Figure 2**. The anodic integrated charge is shown. (**b**) Simultaneously collected SEIRA spectra on the second CV cycle at the potential values indicated, where the background spectrum was collected at –0.1 V vs Ag/AgCl in 0.1 M $NaClO_4$ in the absence of 200 µM **C18-Fc**. (**c**) Identical spectra as shown in b, however, the background spectrum was collected at –0.1 V vs Ag/AgCl in 0.1 M $NaClO_4$ in the presence of 200 µM **C18-Fc**.

Fig. S20a shows the CV of the second cycle immediately following the first cycle shown in **Figure 2**. The charge integration, 33 µC cm^{-2}, of the Fc redox features increases slightly compared to the first scan, **Figure 2c**. This increase lies in contrast to the same charge integration over multiple cycles observed under identical conditions for **C18-Fc** on the Au disk (**Figure S13**), suggesting that the self-assembled layer does not grow upon the application of multiple potential cycles. Therefore, we attribute the slight increase in the charge not due to multilayer formation but rather due to structural changes of the SEIRAS film, leading to an overall roughening of the SEIRAS-active film surface. The simultaneously collected SEIRA spectra reveal near-identical spectroscopic features with the first scan on an absolute basis, **Figure S20b**. The differential spectra, **Figure S20c** (integrations shown in **Figure S21**) are nearly identical to that observed in the initial, first scan, **Figure 2b**. The sole difference is the integration of the ν(CH) of the aliphatic tail, **Figure S21c**; it is reversible with respect to application of the potential. These results suggest that, after the first scan, the interfacial structure of the self-assembled layer converges to a structure in which the aliphatic tail region only reorients in concert with Fc oxidation (**Scheme 3**). The spectroscopic data are consistent with electrochemical data; **C18-Fc** localizes at the Au interface upon addition to the bulk solution via electrostatic templating and persists upon the application of positive potential due to the secondary hydrophobic interaction between the aliphatic tails on the timescale of the slow CV scan.

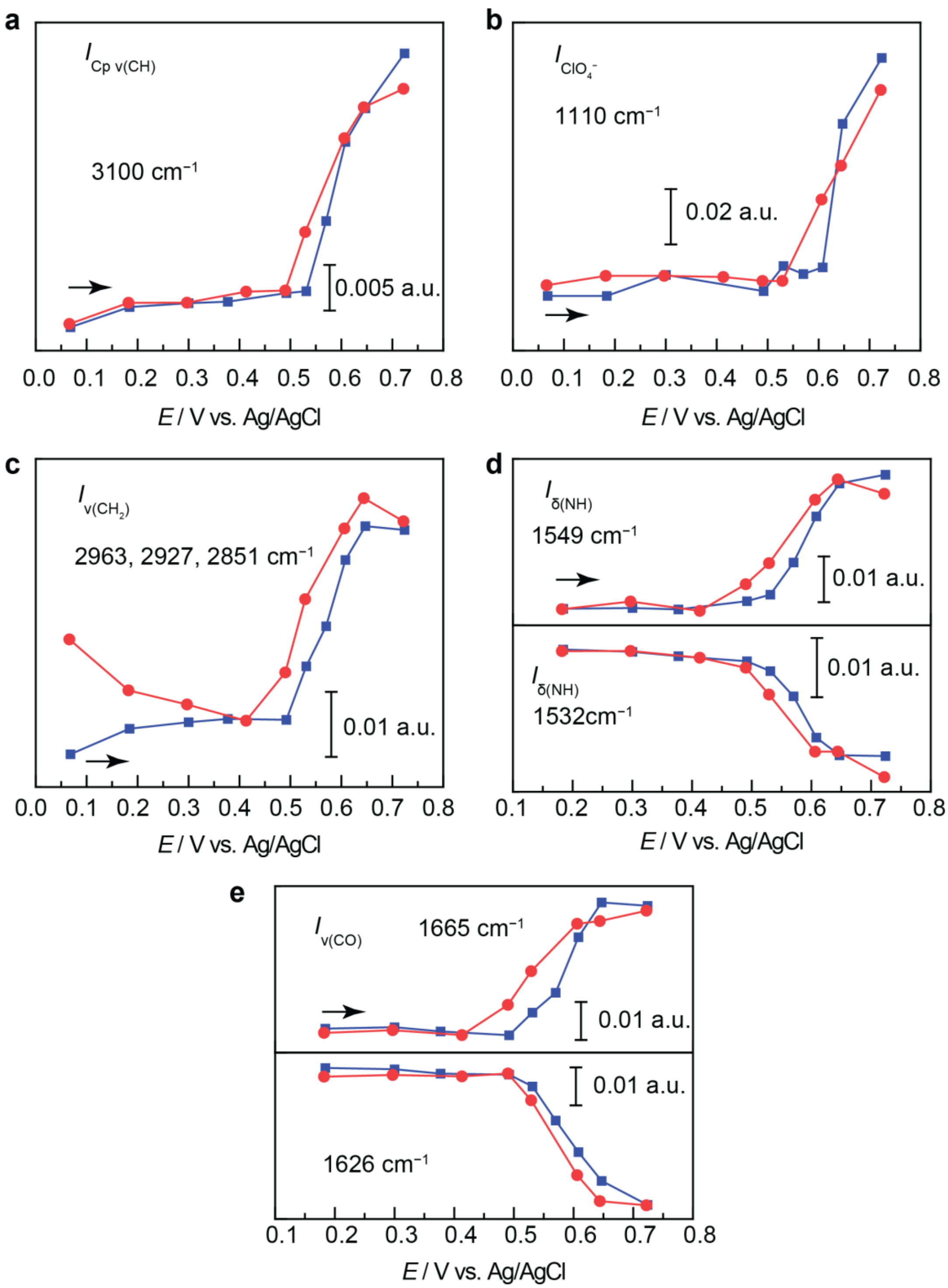

Fig. S21. Integrated band intensities of peak centered at (**a**) 3100; (**b**) 1110; (**c**) 2963, 2927, 2851; (**d**) 1549, 1532; and (**e**) 1665, 1626 cm^{-1} from simultaneously collected SEIRA spectra on a Au film in the presence of 0.1 M $NaClO_4$ in H_2O and 200 μM **C18-Fc** shown in **Figure S19**, second consecutive scan. Blue represents the oxidative trace; red represents the reverse, reductive trace.

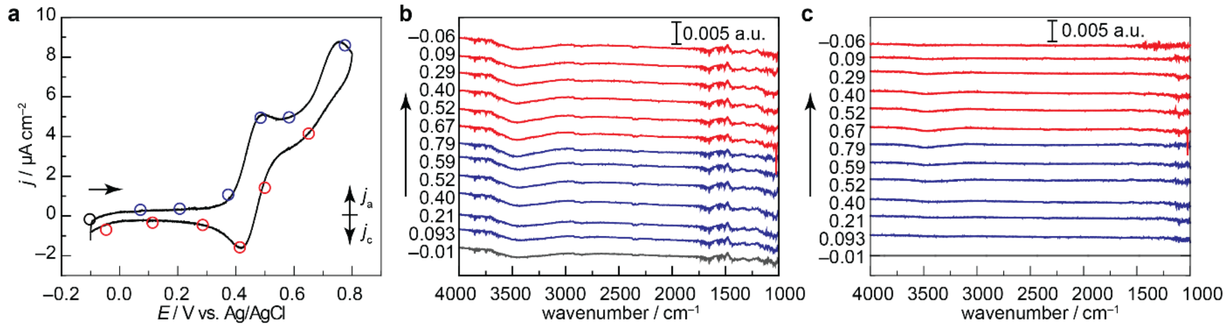

Fig. S22. Cyclic voltammetry (CV) and simultaneously collected SEIRA spectra on a Au film in the presence of 0.1 M $NaClO_4$ in H_2O and 200 μM **C2-Fc**. (a) The initial, first CV cycle collected at 2 mV s^{-1} from –0.1 V vs Ag/AgCl. (b) Simultaneously collected SEIRA spectra on the first CV cycle at the potential values indicated, where the background spectrum was collected at –0.1 V vs Ag/AgCl in 0.1 M $NaClO_4$ in the absence of **C2-Fc**. (c) Identical spectra as shown in b, however, the background spectrum was collected at –0.1 V vs Ag/AgCl in 0.1 M $NaClO_4$ in the presence of 200 μM **C2-Fc**.

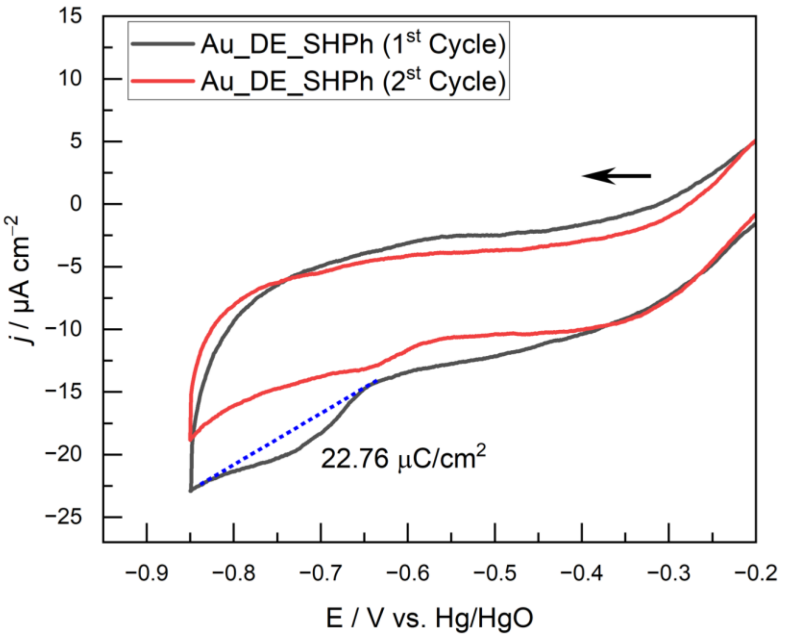

Fig. S23. Cyclic voltammogram of Au disk electrode modified with benzenethiol recorded in 0.5 M KOH at 10 mV s^{-1} with a negative direction of scan. Electrode immersed for 1 minute in 1 mM benzenethiol aqueous solution prior to use.

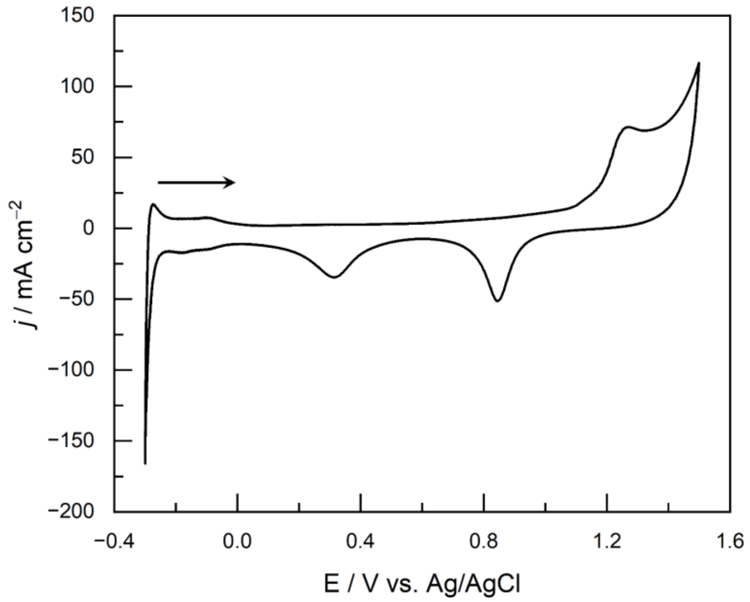

Fig. S24. Cyclic voltammogram of electrodeposited Pt nanoparticles on Au electrode in aqueous 0.5 M H_2SO_4 electrolyte solution at 100 mV s^{-1} with a positive direction of scan.

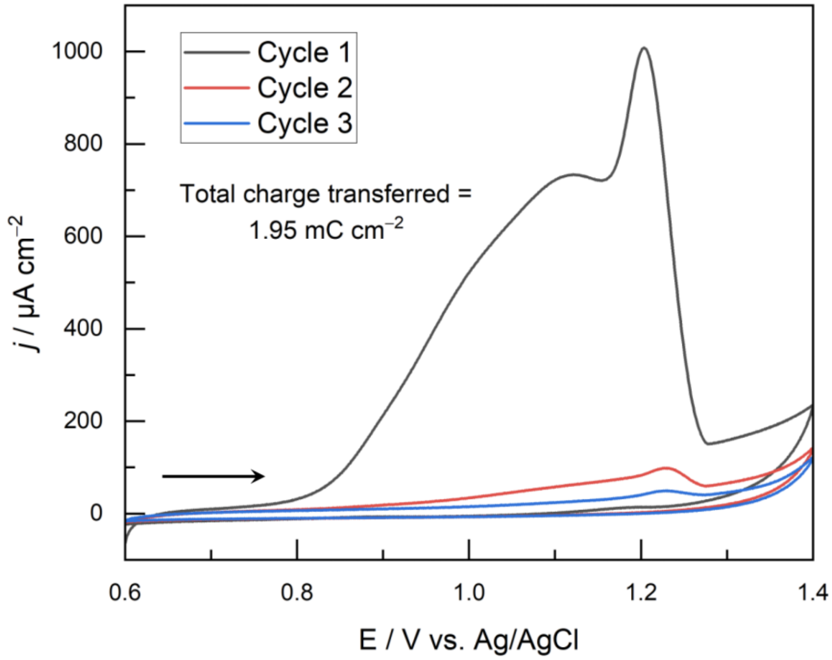

Fig. S25. Cyclic voltammograms of electrodeposited Au nanoparticles on glassy carbon electrode in aqueous 0.1 M HCl electrolyte solution at 100 mV s^{-1} with a positive direction of scan.

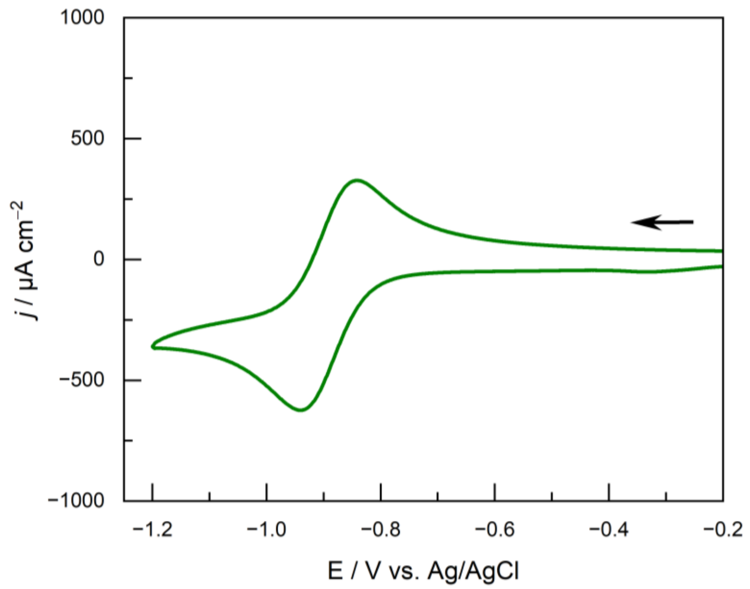

Fig. S26. Cyclic voltammogram of glassy carbon disk electrode modified with FeOEPCl recorded in electrolyte solution of 0.1 M TBAP in acetonitrile at 100 mV s^{-1} with negative direction of scan. Electrode modified using a dropcast method with 5 μL ink prepared from 1.3 mg FeOEPCl/mL stock solution (see **Table S7**).

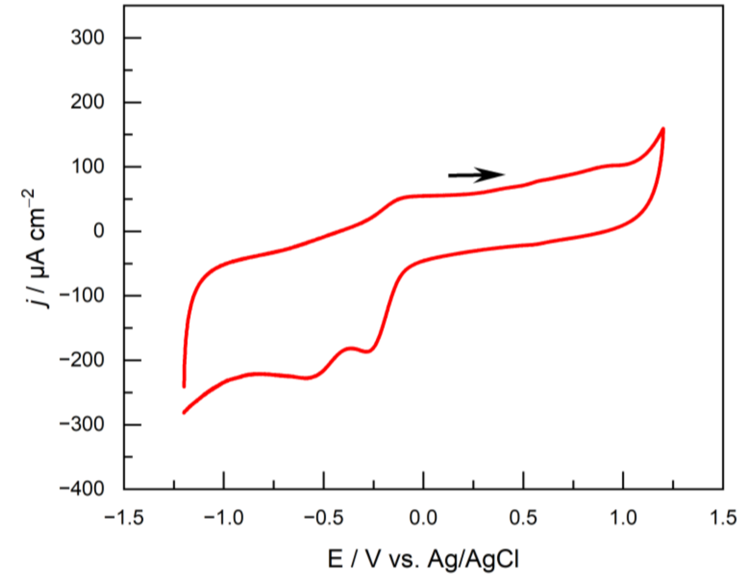

Fig. S27. Cyclic voltammogram of glassy carbon disk electrode modified with cobalt phthalocyanine (CoPc) recorded in electrolyte solution of 0.1 M TBAP in acetonitrile at 100 mV s^{-1} with positive direction of scan. Electrode modified using a dropcast method with 10 μL ink prepared from 8.3 mg CoPc/mL stock solution (see **Table S7**).

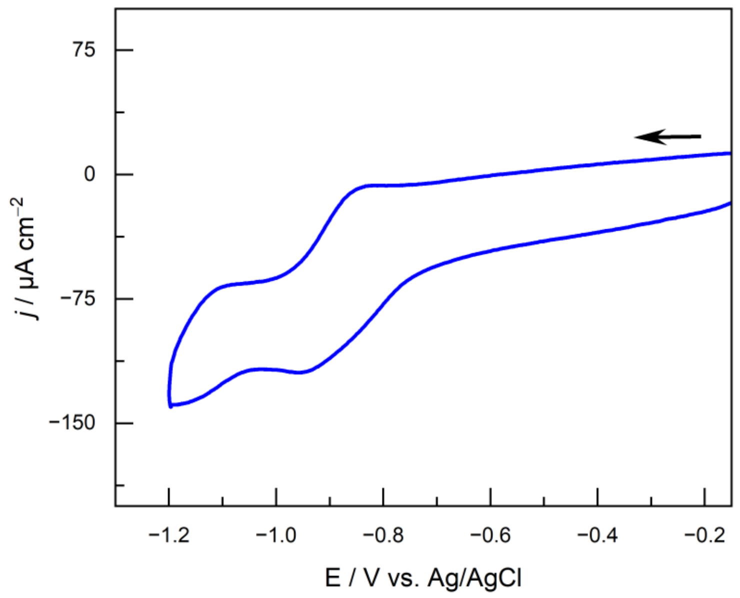

Fig. S28. Cyclic voltammogram of glassy carbon disk electrode modified with zinc phthalocyanine (ZnPc) recorded in electrolyte solution of 0.1 M TBAP in acetonitrile at 100 mV s^{-1} with negative direction of scan. Electrode modified using a dropcast method with 5 μL ink prepared from 7.9 mg ZnPc/mL stock solution (see **Table S7**).

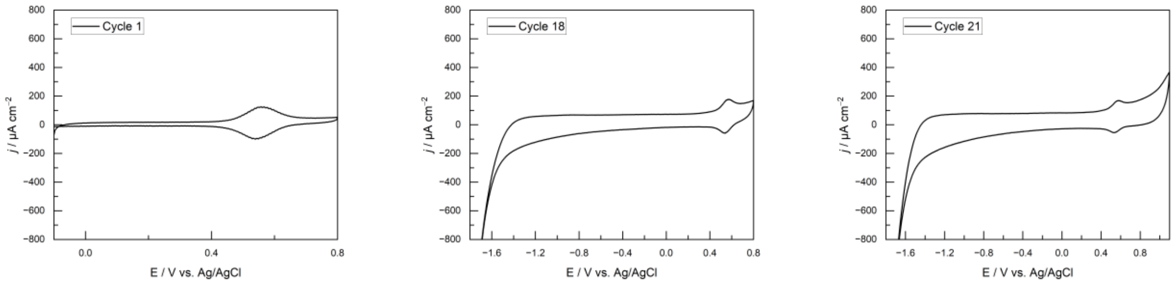

Fig. S29. Cyclic voltammograms of a glassy carbon foil electrode in the presence of 135 μM **C18-Fc** and 0.1 M $NaClO_4$ at 100 mV s^{-1}. Data taken from the plot shown in **Figure 4**. CVs conducted with a positive direction of scan.

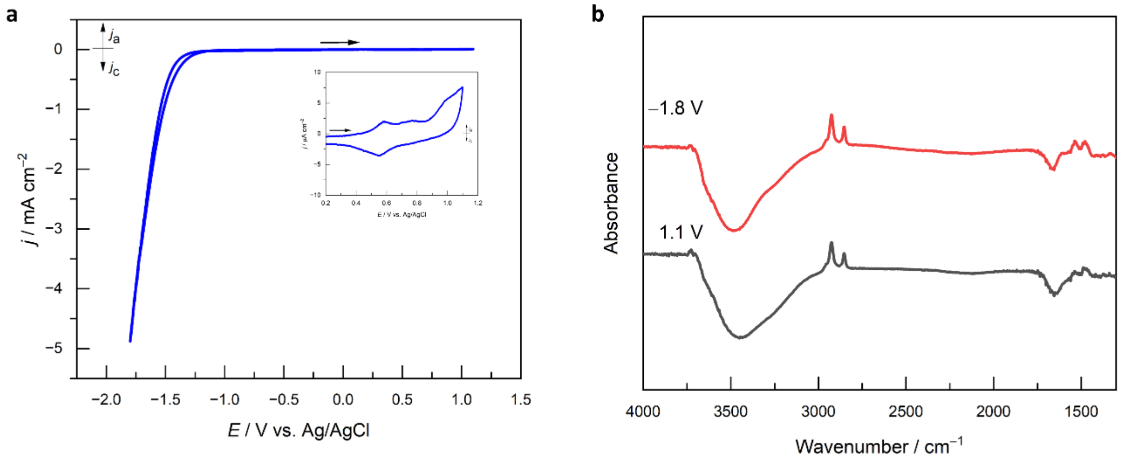

Fig. S30. SEIRA data taken over a wide potential window on Au in the presence of **C18-Fc**. (a) The first CV cycle collected in the presence of **C18-Fc** (200 μM) with 2 mV/sec scan rate in 0.1 M $NaClO_4$. The scan was initiated at −0.1 V with a positive direction of scan. (b) Simultaneously collected SEIRA spectra on the first scan of CV cycle at 1.1 V and −1.8 V *vs* Ag/AgCl. Background spectrum taken at − 0.1 V vs Ag/AgCl in 0.1 M $NaClO_4$ in the absence of **C18-Fc**.

Structural Characterization of Synthesized Compounds

Compound Table

Label	Tgt Score	Mass Error (ppm)	MFG Formula	Tgt Formula	Obs. RT	Ref. Mass	Obs. Mass
Cpd 1: C18 H37 N O2	87.29	-5.56	C18 H37 N O2	C18 H37 N O2	0.374	299.2824	299.2808

Obs. m/z	Obs. RT	Obs. Mass	Tgt Formula	Tgt Mass	Tgt Mass Error	Find Cpds Algorit
300.2879	0.374	299.2808	C18 H37 N O2	299.2824	-5.56	Find By Formula

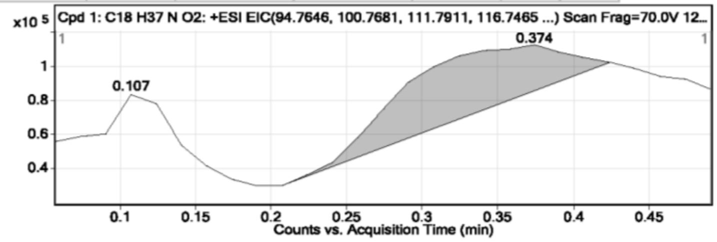

MS Zoomed Spectrum

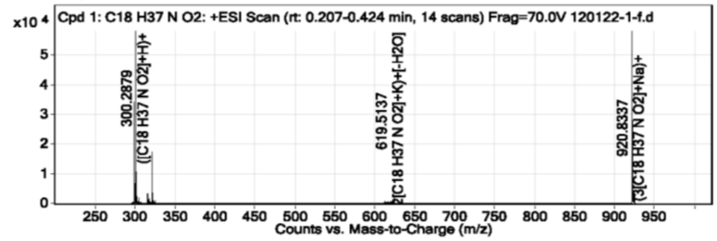

MS Spectrum Peak List

Obs. m/z	Charge	Abund	Formula	Ion/Isotope	Tgt Mass Error (ppm)
300.2879	1	58885.7	C18 H37 N O2	(M+H)+	6.03
301.2912	1	10861.97	C18 H37 N O2	(M+H)+	6.08
302.2986	1	2381.61	C18 H37 N O2	(M+H)+	-8.81
320.2305	1	317.07	C18 H37 N O2	(M+K)+[-H2O]	14.23
616.5968	1	290.43	C18 H37 N O2	(2M+NH4)+	3.08
617.5925	1	204.2	C18 H37 N O2	(2M+NH4)+	15.36
618.608	1	226.57	C18 H37 N O2	(2M+NH4)+	-4.9
619.5137	1	881.24	C18 H37 N O2	(2M+K)+[-H2O]	6.01
920.8337	1	60.75	C18 H37 N O2	(3M+Na)+	3.02

--- End Of Report ---

HRMS of 2-aminooctadecanoic acid (**1**).

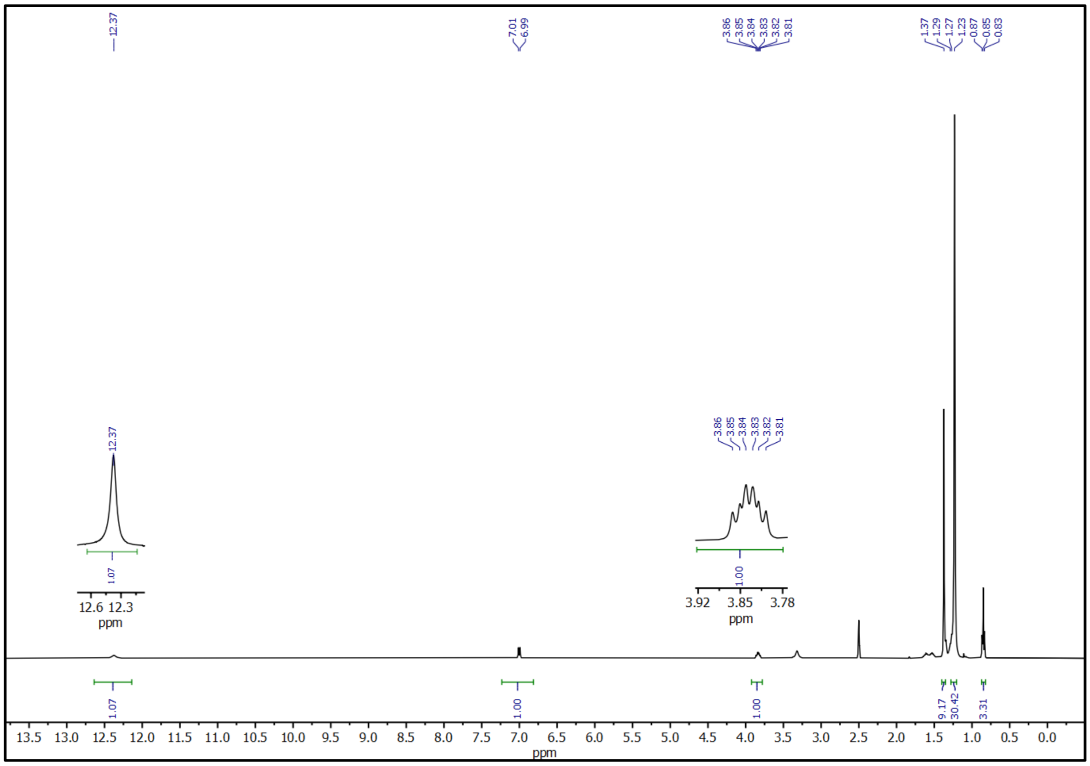

^{1}H NMR of 2-((tert-butoxycarbonyl)amino)octadecanoic acid **(2)** in DMSO-d_6.

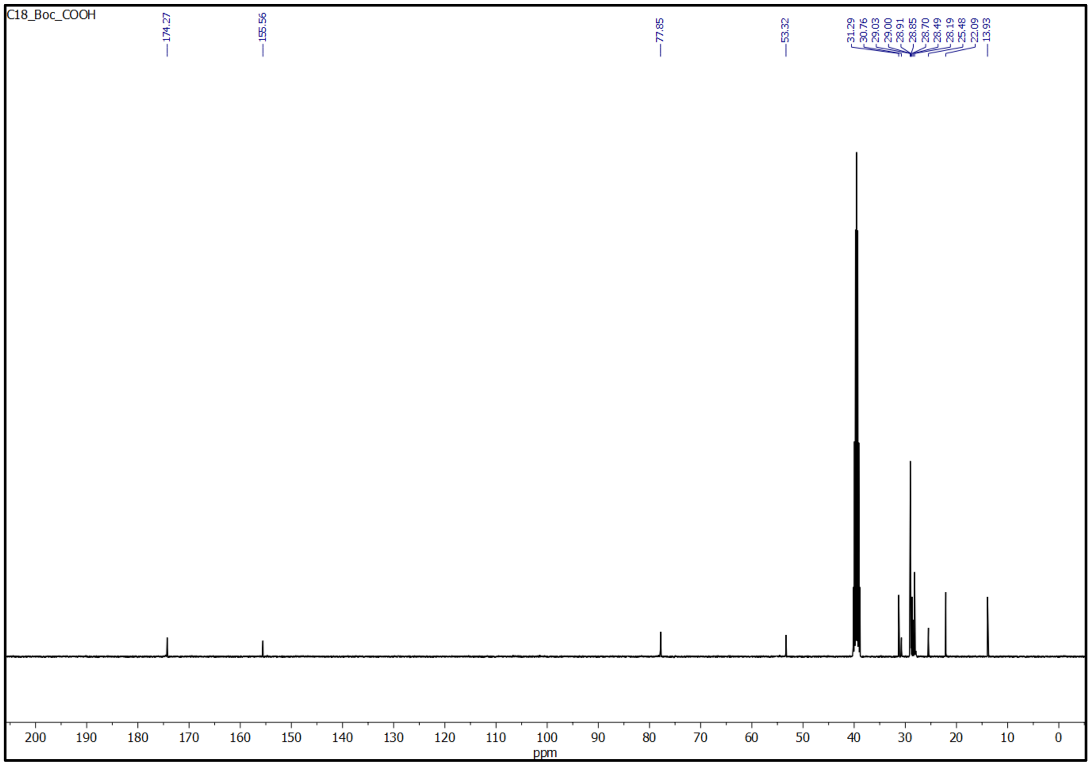

^{13}C NMR of 2-((tert-butoxycarbonyl)amino)octadecanoic acid **(2)** in DMSO-d_6.

Compound Table

Label	Tgt Score	Mass Error (ppm)	MFG Formula	Tgt Formula	Obs. RT	Ref. Mass	Obs. Mass
Cpd 1: C23 H45 N O4	98.23	1.39	C23 H45 N O4	C23 H45 N O4	0.122	399.3349	399.3354

Obs. m/z	Obs. RT	Obs. Mass	Tgt Formula	Tgt Mass	Tgt Mass Error	Find Cpds Algorit
422.3248	0.122	399.3354	C23 H45 N O4	399.3349	1.39	Find By Formula

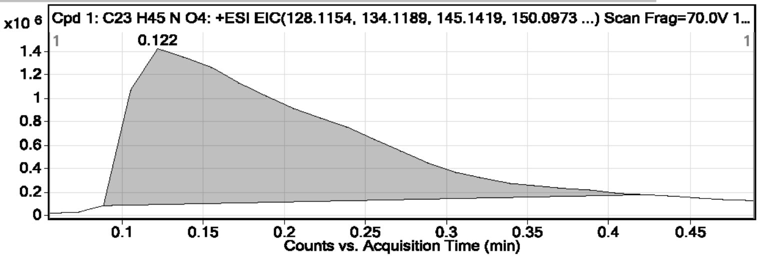

MS Zoomed Spectrum

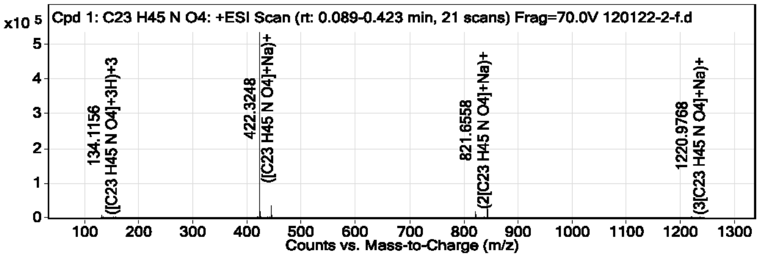

MS Spectrum Peak List

Obs. m/z	Charge	Abund	Formula	Ion/Isotope	Tgt Mass Error (ppm)
134.1156	3	2265.92	C23 H45 N O4	(M+3H)+3	24.55
156.1053	3	316.1	C23 H45 N O4	(M+3Na)+3	-28.42
400.3319	1	437.83	C23 H45 N O4	(M+H)+	25.46
422.3248	1	538935.98	C23 H45 N O4	(M+Na)+	-1.74
438.2993	1	3620.72	C23 H45 N O4	(M+K)+	-2.88
819.5982	1	170.81	C23 H45 N O4	(2M+K)+[-H2O]	29.4
821.6558	1	18696.11	C23 H45 N O4	(2M+Na)+	3.86
837.62	1	3793.91	C23 H45 N O4	(2M+K)+	15.38
1220.9768	1	2215.95	C23 H45 N O4	(3M+Na)+	13.9
1236.9541	1	2111.49	C23 H45 N O4	(3M+K)+	11.06

--- End Of Report ---

HRMS of 2-((tert-butoxycarbonyl)amino)octadecanoic acid **(2)**.

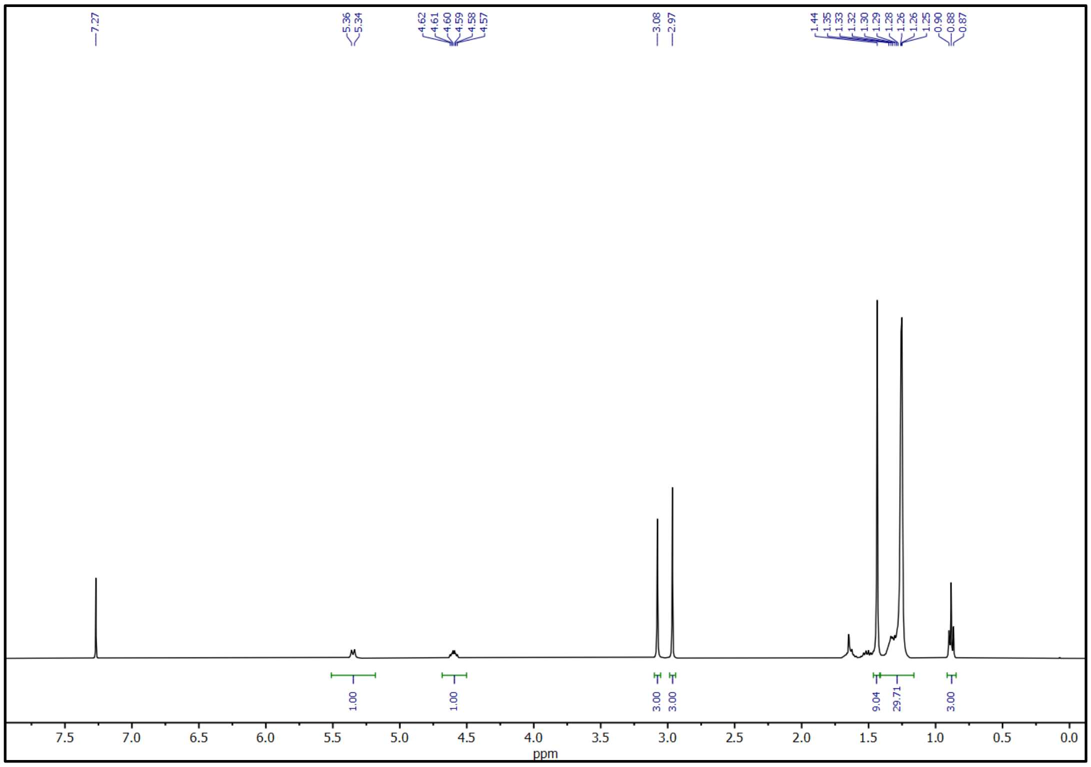

^{1}H NMR of *tert*-butyl (1-(dimethylamino)-1-oxooctadecan-2-yl)carbamate **(3)** in $CDCl_3$.

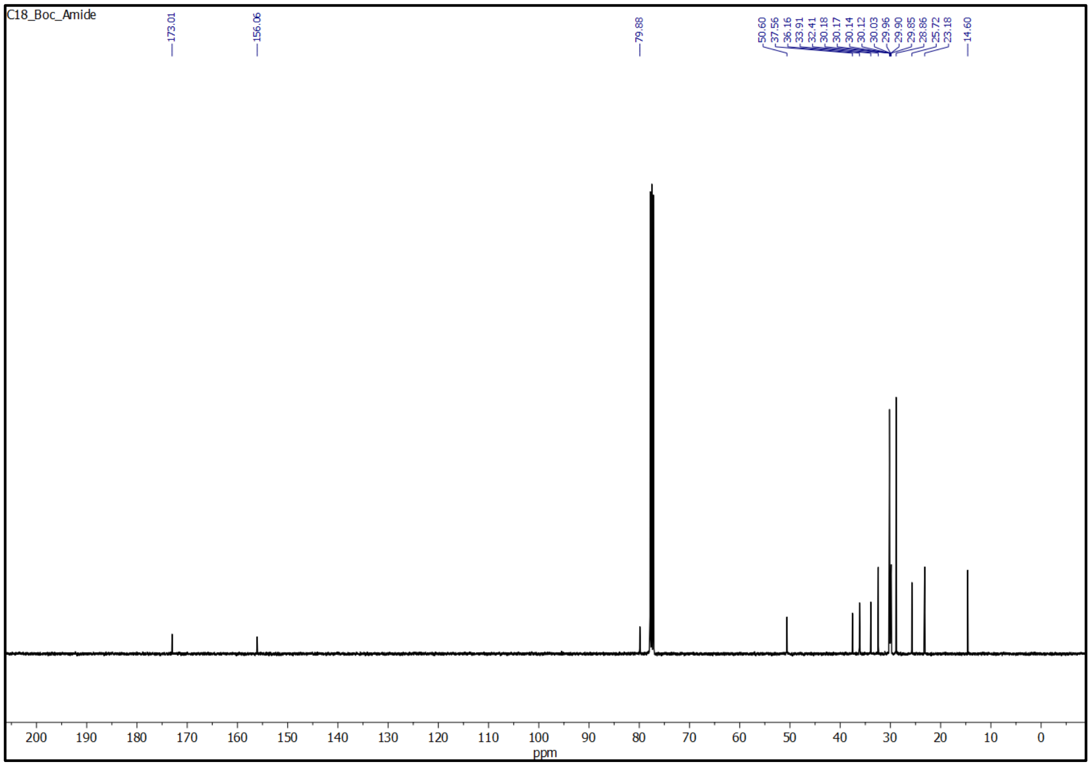

^{13}C NMR of *tert*-butyl (1-(dimethylamino)-1-oxooctadecan-2-yl)carbamate **(3)** in $CDCl_3$.

Compound Table

Label	Tgt Score	Mass Error (ppm)	MFG Formula	Tgt Formula	Obs. RT	Ref. Mass	Obs. Mass
Cpd 1: C25 H50 N2 O3	99.57	0.68	C25 H50 N2 O3	C25 H50 N2 O3	0.121	426.3821	426.3824

Obs. m/z	Obs. RT	Obs. Mass	Tgt Formula	Tgt Mass	Tgt Mass Error	Find Cpds Algorit
449.3717	0.121	426.3824	C25 H50 N2 O3	426.3821	0.68	Find By Formula

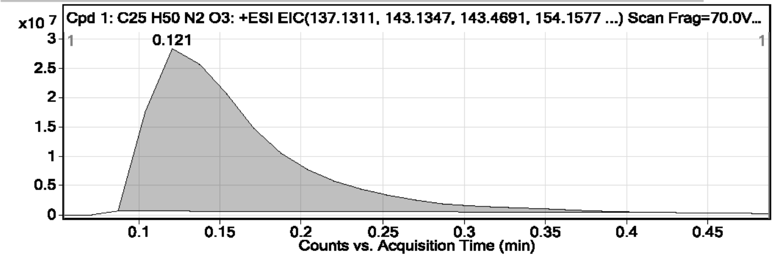

MS Zoomed Spectrum

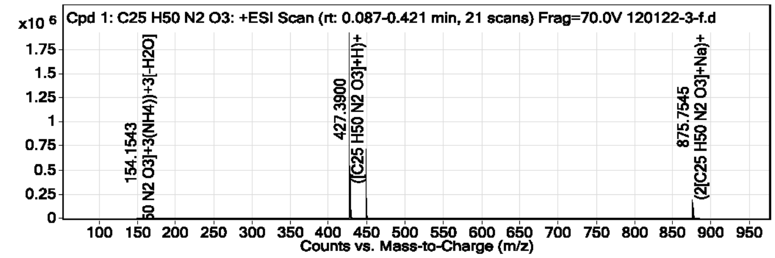

MS Spectrum Peak List

Obs. m/z	Charge	Abund	Formula	Ion/Isotope	Tgt Mass Error (ppm)
154.1543	3	118.14	C25 H50 N2 O3	(M+3(NH4))+3[-H2O]	21.8
427.39	1	1925691.69	C25 H50 N2 O3	(M+H)+	-1.26
428.3931	1	554680.07	C25 H50 N2 O3	(M+H)+	-0.94
447.3439	1	2625.15	C25 H50 N2 O3	(M+K)+[-H2O]	-20.38
449.3717	1	739629.85	C25 H50 N2 O3	(M+Na)+	-0.65
450.3751	1	206702.16	C25 H50 N2 O3	(M+Na)+	-1.04
465.3447	1	4507.73	C25 H50 N2 O3	(M+K)+	1.23
852.7735	1	92.21	C25 H50 N2 O3	(2M+NH4)+[-H2O]	16.53
853.7806	1	91.81	C25 H50 N2 O3	(2M+H)+	-10.53
875.7545	1	192208.4	C25 H50 N2 O3	(2M+Na)+	-1.1

--- End Of Report ---

HRMS of *tert*-butyl (1-(dimethylamino)-1-oxooctadecan-2-yl)carbamate **(3)**.

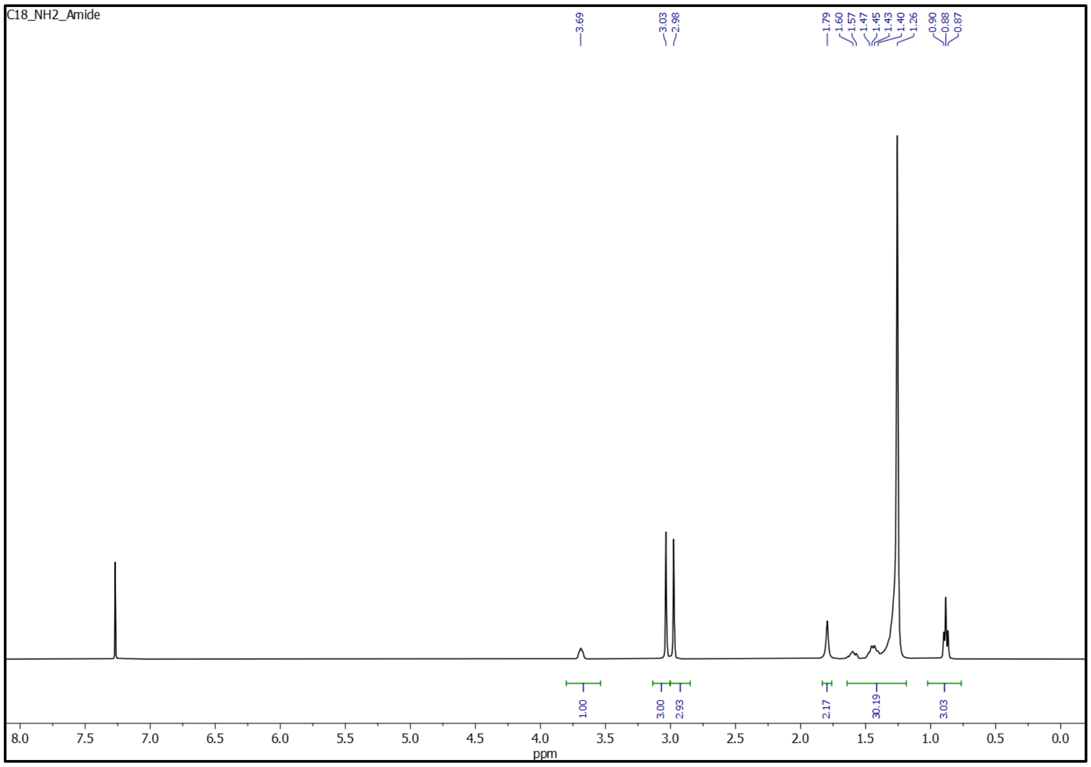

^{1}H NMR of 2-amino-*N,N*-dimethyloctadecanamide **(4)** in $CDCl_3$.

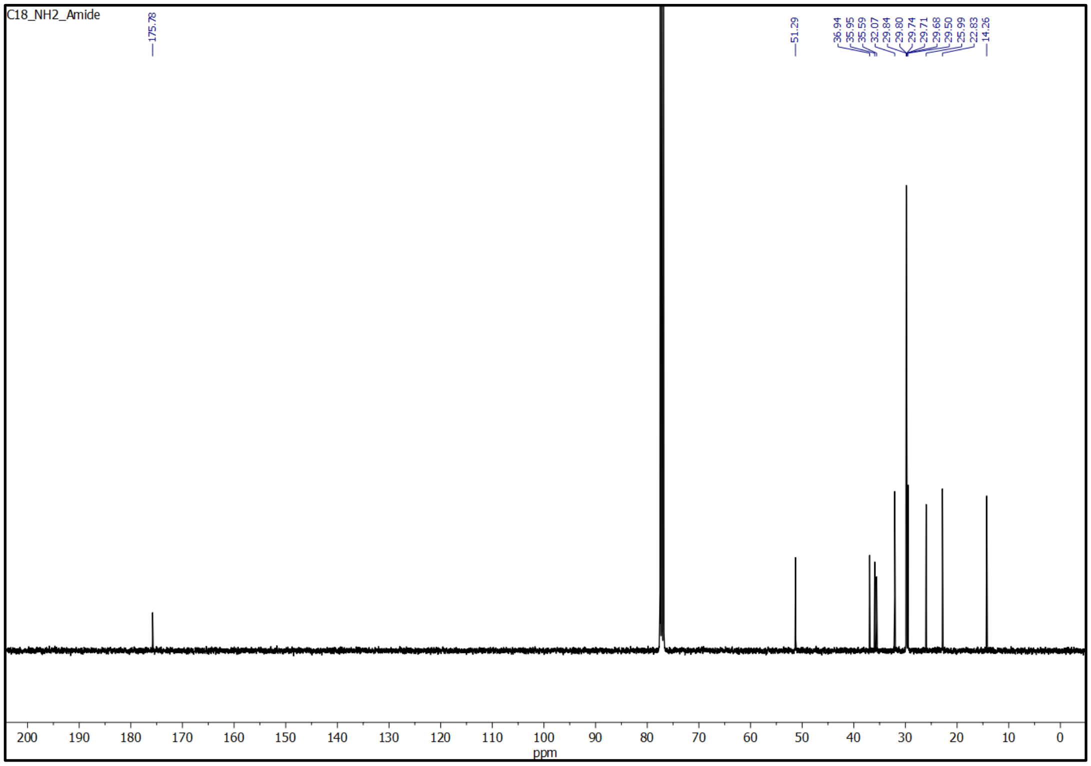

^{13}C NMR of 2-amino-*N,N*-dimethyloctadecanamide **(4)** in $CDCl_3$.

Compound Table

Label	Tgt Score	Mass Error (ppm)	MFG Formula	Tgt Formula	Obs. RT	Ref. Mass	Obs. Mass
Cpd 1: C20 H42 N2 O	97.37	1.79	C20 H42 N2 O	C20 H42 N2 O	0.121	326.3297	326.3303

Obs. m/z	Obs. RT	Obs. Mass	Tgt Formula	Tgt Mass	Tgt Mass Error	Find Cpds Algorit
675.6496	0.121	326.3303	C20 H42 N2 O	326.3297	1.79	Find By Formula

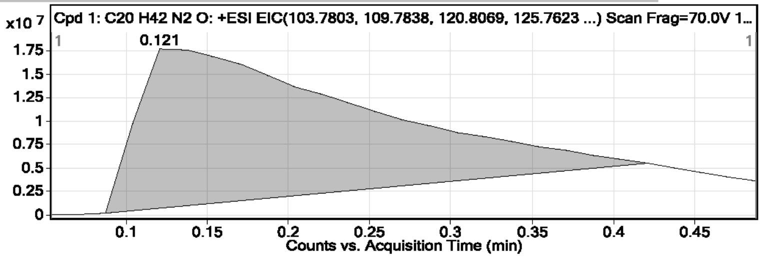

MS Zoomed Spectrum

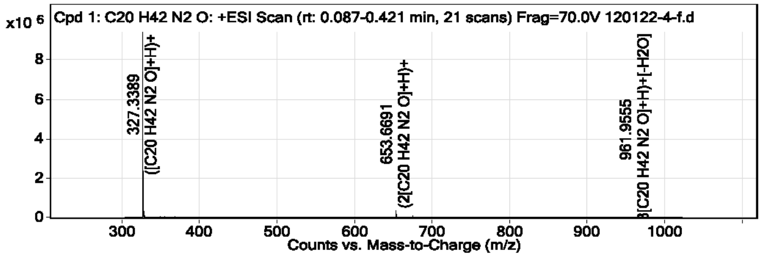

MS Spectrum Peak List

Obs. m/z	Charge	Abund	Formula	Ion/Isotope	Tgt Mass Error (ppm)
327.3389	1	9521176.69	C20 H42 N2 O	(M+H)+	-5.97
349.3198	1	54063.03	C20 H42 N2 O	(M+Na)+	-2.61
365.2964	1	1104.33	C20 H42 N2 O	(M+K)+	-9.72
653.6691	1	353355.58	C20 H42 N2 O	(2M+H)+	-3.64
657.6293	1	947.62	C20 H42 N2 O	(2M+Na)+[-H2O]	13.35
675.6496	1	80335.54	C20 H42 N2 O	(2M+Na)+	-1.37
691.6311	1	271.52	C20 H42 N2 O	(2M+K)+	-12.24
961.9555	1	1601.69	C20 H42 N2 O	(3M+H)+[-H2O]	31.61
1001.9818	1	532.7	C20 H42 N2 O	(3M+Na)+	-3.45
1017.9232	1	126.36	C20 H42 N2 O	(3M+K)+	28.63

--- End Of Report ---

HRMS of 2-amino-*N,N*-dimethyloctadecanamide **(4)**.

Compound Table

Label	Tgt Score	Mass Error (ppm)	MFG Formula	Tgt Formula	Obs. RT	Ref. Mass	Obs. Mass
Cpd 1: C20 H44 N2	97.58	-0.65	C20 H44 N2	C20 H44 N2	0.137	312.3504	312.3502

Obs. m/z	Obs. RT	Obs. Mass	Tgt Formula	Tgt Mass	Tgt Mass Error	Find Cpds Algorith
313.3577	0.137	312.3502	C20 H44 N2	312.3504	-0.65	Find By Formula

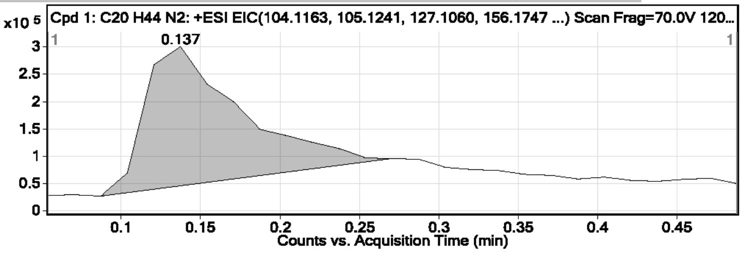

MS Zoomed Spectrum

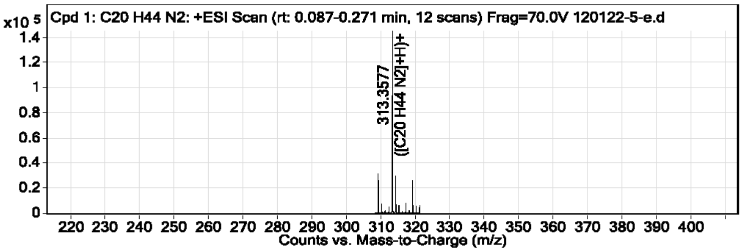

MS Spectrum Peak List

Obs. m/z	Charge	Abund	Formula	Ion/Isotope	Tgt Mass Error (ppm)
313.3577	1	147800.43	C20 H44 N2	(M+H)+	0.06
314.3602	1	30156.85	C20 H44 N2	(M+H)+	2.45
315.3618	1	3588.07	C20 H44 N2	(M+H)+	7.51
316.3576	1	287.96	C20 H44 N2	(M+H)+	30.98

--- End Of Report ---

HRMS of *N,N*-dimethyloctadecane-1,2-diamine **(5)**.

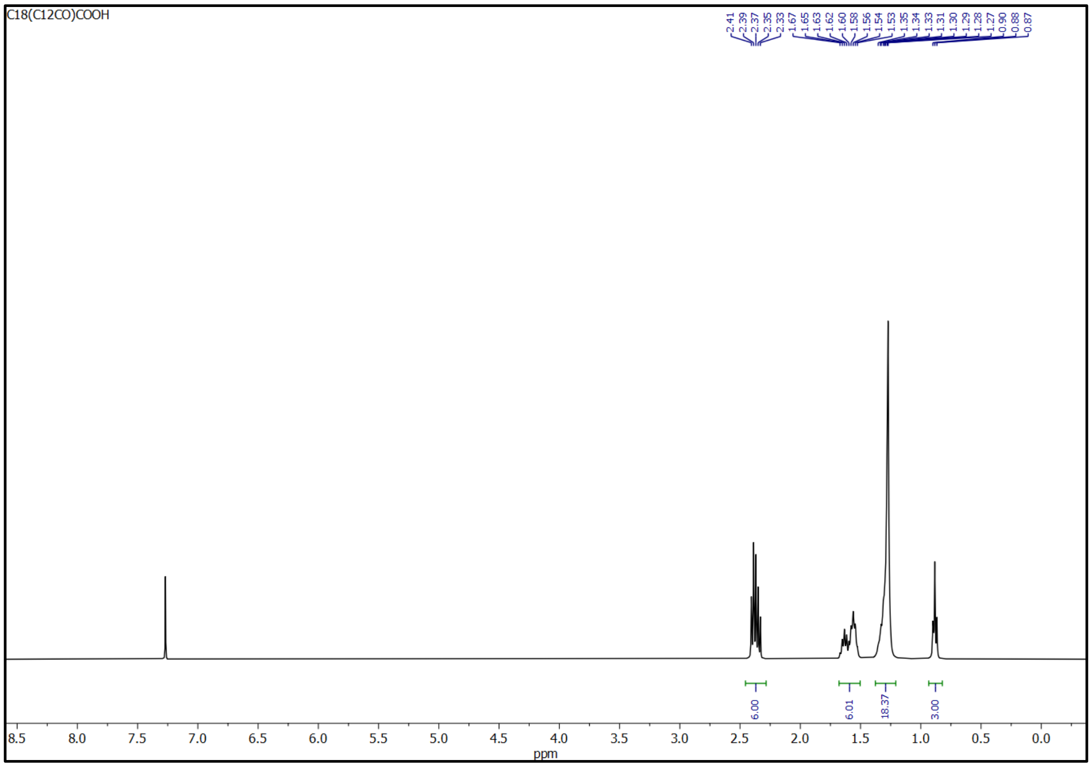

^{1}H NMR of 12-Oxooctadecanoic acid (**6**) in $CDCl_3$.

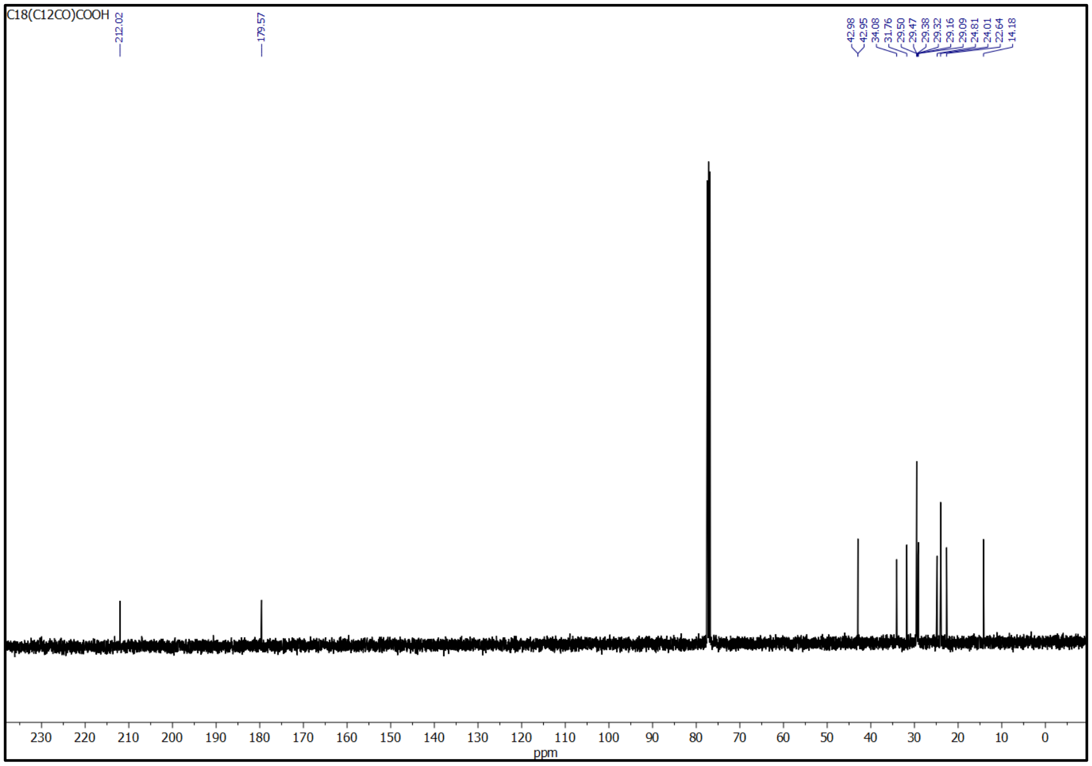

^{13}C NMR of 12-Oxooctadecanoic acid **(6)** in $CDCl_3$.

Compound Table

Label	Tgt Score	Mass Error (ppm)	MFG Formula	Tgt Formula	Obs. RT	Ref. Mass	Obs. Mass
Cpd 1: C18 H34 O3	87.01	-6.99	C18 H34 O3	C18 H34 O3	0.118	298.2508	298.2487

Obs. m/z	Obs. RT	Obs. Mass	Tgt Formula	Tgt Mass	Tgt Mass Error	Find Cpds Algorith
333.2182	0.118	298.2487	C18 H34 O3	298.2508	-6.99	Find By Formula

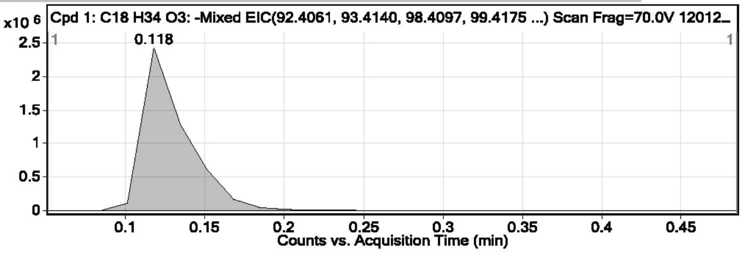

MS Zoomed Spectrum

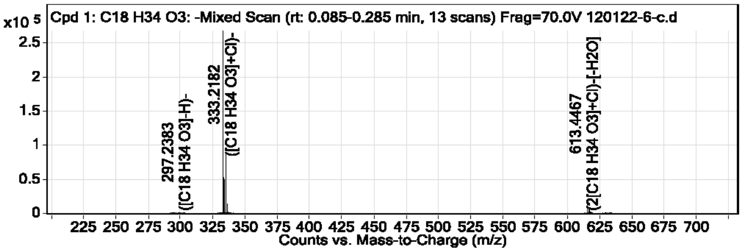

MS Spectrum Peak List

Obs. m/z	Charge	Abund	Formula	Ion/Isotope	Tgt Mass Error (ppm)
297.2383	1	276.79	C18 H34 O3	(M-H)-	17.65
298.2409	1	43.53	C18 H34 O3	(M-H)-	20.21
333.2182	1	269071.86	C18 H34 O3	(M+Cl)-	5.88
334.2212	1	49954.53	C18 H34 O3	(M+Cl)-	7.28
335.2159	1	86922.78	C18 H34 O3	(M+Cl)-	5.95
336.2174	1	13817.5	C18 H34 O3	(M+Cl)-	10.66
337.2181	1	1226.07	C18 H34 O3	(M+Cl)-	16.37
613.4467	1	327.07	C18 H34 O3	(2M+Cl)-[-H2O]	22.37
614.448	1	103.31	C18 H34 O3	(2M+Cl)-[-H2O]	25.8
631.4515	1	92.27	C18 H34 O3	(2M+Cl)-	30.94

--- End Of Report ---

HRMS of 12-Oxooctadecanoic acid **(6).**

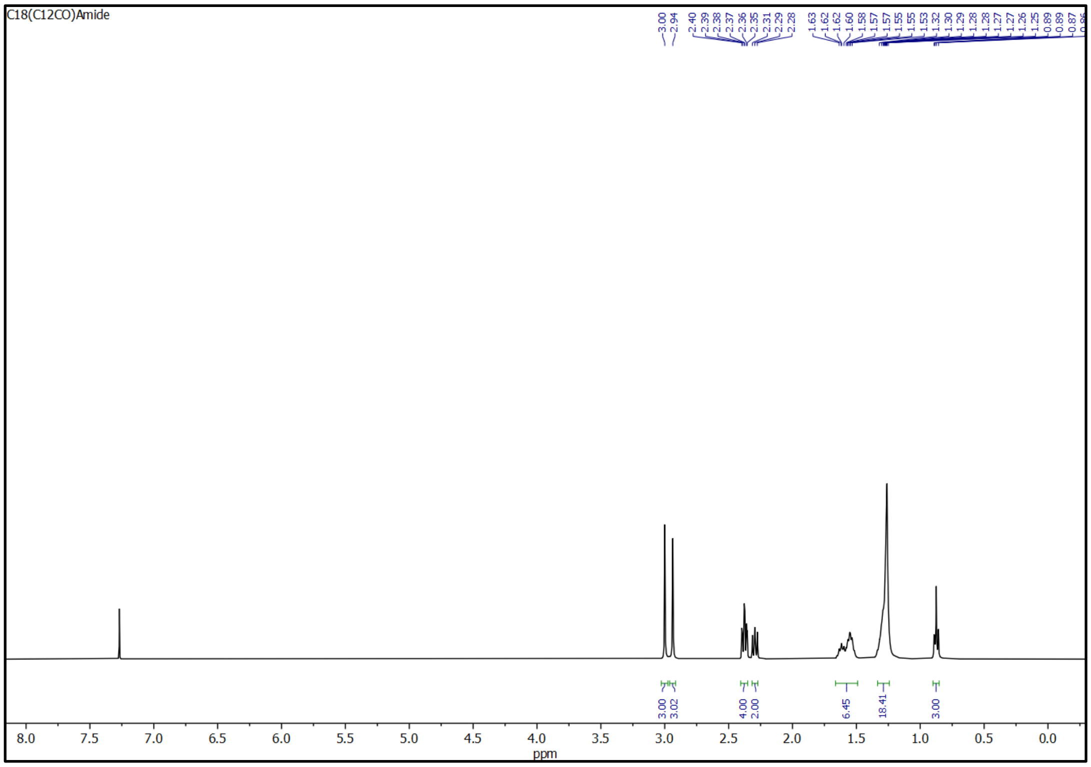

^{1}H NMR of *N,N*-Dimethyl-12-oxooctadecanamide **(7)** in $CDCl_3$.

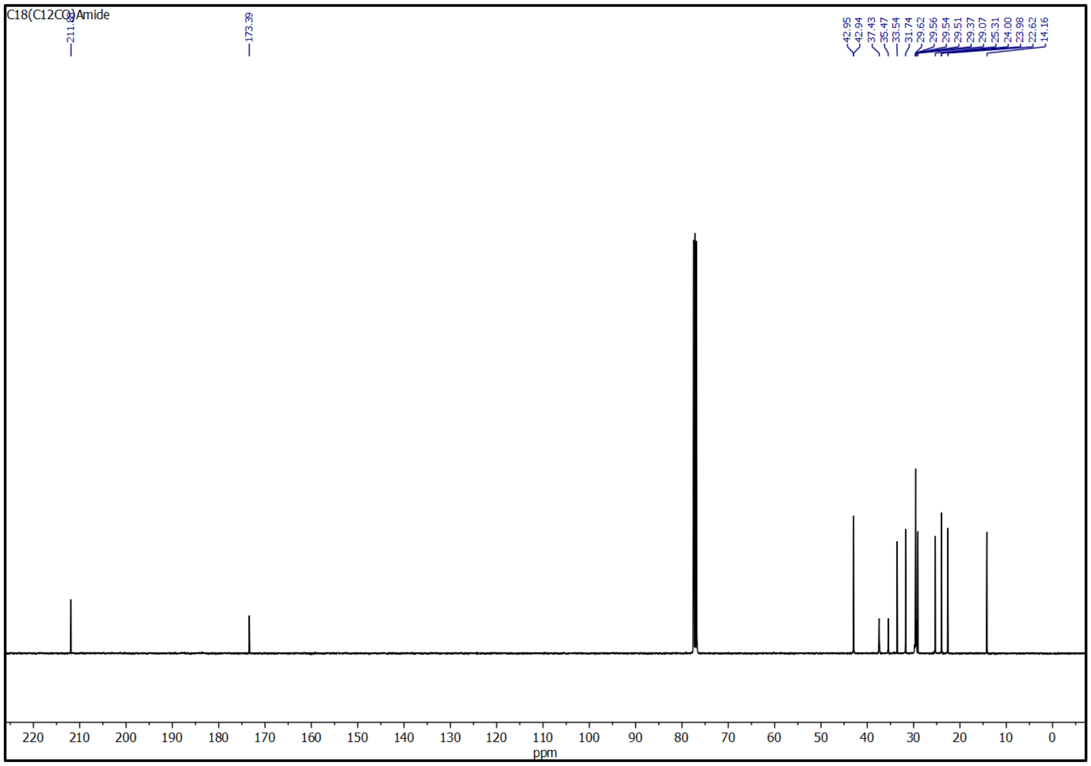

^{13}C NMR of *N,N*-Dimethyl-12-oxooctadecanamide **(7)** in $CDCl_3$.

Compound Table

Label	Tgt Score	Mass Error (ppm)	MFG Formula	Tgt Formula	Obs. RT	Ref. Mass	Obs. Mass
Cpd 1: C20 H39 N O2	94.74	3.34	C20 H39 N O2	C20 H39 N O2	0.179	325.2981	325.2992

Obs. m/z	Obs. RT	Obs. Mass	Tgt Formula	Tgt Mass	Tgt Mass Error	Find Cpds Algorith
348.2886	0.179	325.2992	C20 H39 N O2	325.2981	3.34	Find By Formula

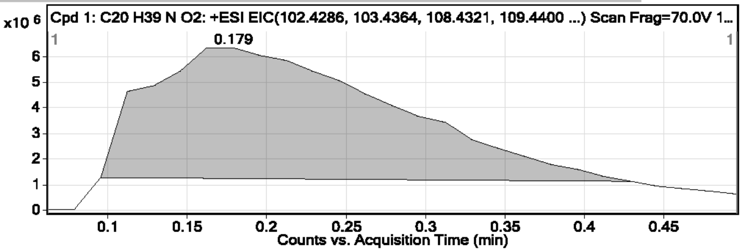

MS Zoomed Spectrum

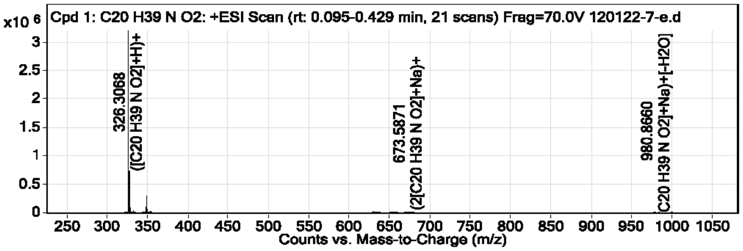

MS Spectrum Peak List

Obs. m/z	Charge	Abund	Formula	Ion/Isotope	Tgt Mass Error (ppm)
326.3068	1	3215000.15	C20 H39 N O2	(M+H)+	-4.39
327.3104	1	736558.41	C20 H39 N O2	(M+H)+	-5.43
328.313	1	92339.19	C20 H39 N O2	(M+H)+	-4.15
330.2767	1	2437.65	C20 H39 N O2	(M+Na)+[-H2O]	0.02
348.2886	1	300145.06	C20 H39 N O2	(M+Na)+	-3.68
349.291	1	62348.12	C20 H39 N O2	(M+Na)+	-0.98
633.574	1	2345.06	C20 H39 N O2	(2M+H)+[-H2O]	29.83
655.5578	1	371.25	C20 H39 N O2	(2M+Na)+[-H2O]	25.88
673.5871	1	2483.69	C20 H39 N O2	(2M+Na)+	-2.62
980.866	1	109.34	C20 H39 N O2	(3M+Na)+[-H2O]	7.06

--- End Of Report ---

HRMS of *N,N*-Dimethyl-12-oxooctadecanamide **(7)**.

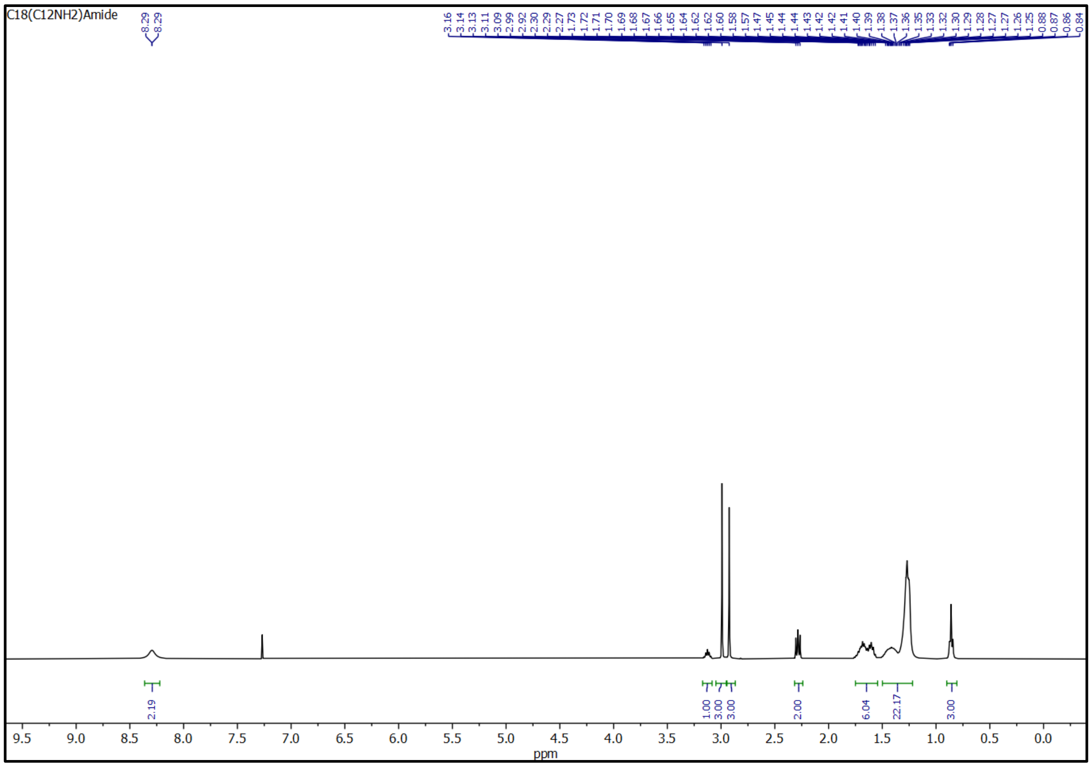

^{1}H NMR of 12-Amino-*N,N*-dimethyloctadecanamide **(8)** in $CDCl_3$.

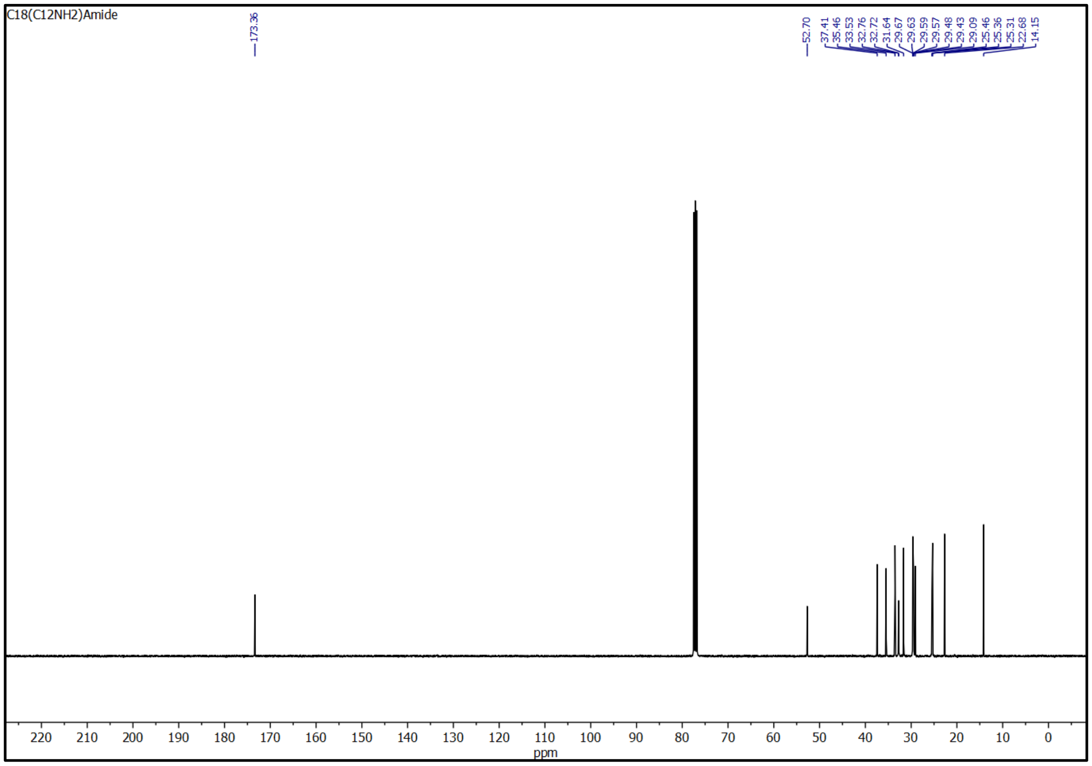

^{13}C NMR of 12-Amino-*N,N*-dimethyloctadecanamide **(8)** in $CDCl_3$.

Compound Table

Label	Tgt Score	Mass Error (ppm)	Tgt Formula	Obs. RT	Ref. Mass	Obs. Mass
Cpd 1: C20 H42 N2 O	83.18	5.95	C20 H42 N2 O	0.133	326.3297	326.3317

Obs. m/z	Obs. RT	Obs. Mass	Tgt Formula	Tgt Mass	Tgt Mass Error	Find Cpds Algorith
327.3393	0.133	326.3317	C20 H42 N2 O	326.3297	5.95	Find By Formula

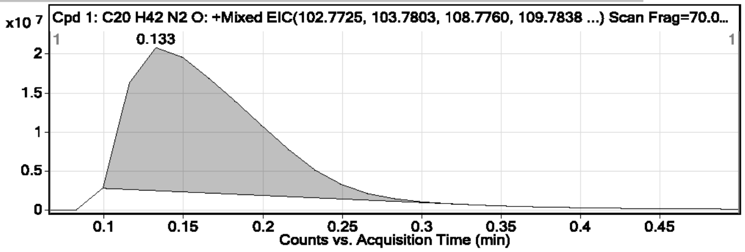

MS Zoomed Spectrum

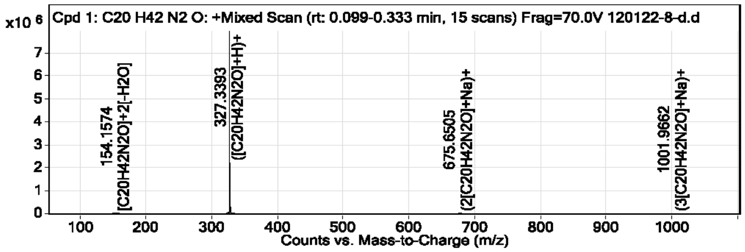

MS Spectrum Peak List

Obs. m/z	Charge	Abund	Formula	Ion/Isotope	Tgt Mass Error (ppm)
154.1574	2	7432.64	C20H42N2O	M+2[-H2O]	10.64
327.3393	1	8084832.95	C20H42N2O	(M+H)+	-7
328.341	1	2200980.82	C20H42N2O	(M+H)+	-2.43
329.3442	1	280896.6	C20H42N2O	(M+H)+	-3.01
330.3481	1	19738.93	C20H42N2O	(M+H)+	-5.87
675.6505	1	1394.62	C20H42N2O	(2M+Na)+	-2.8
1001.9662	1	139.97	C20H42N2O	(3M+Na)+	12.12
1002.9602	1	133.98	C20H42N2O	(3M+Na)+	21.33

--- End Of Report ---

HRMS of 12-Amino-*N,N*-dimethyloctadecanamide **(8)**.

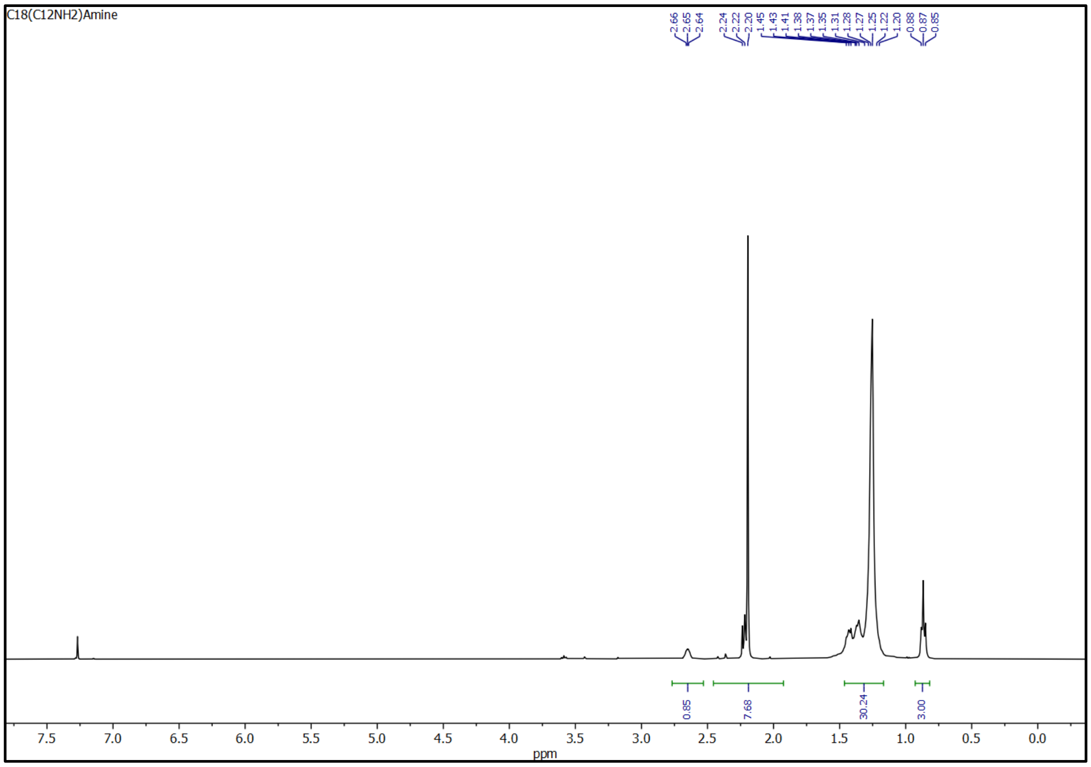

^{1}H NMR of *N,N*-Dimethyloctadecane-1,12-diamine **(9)** in $CDCl_3$.

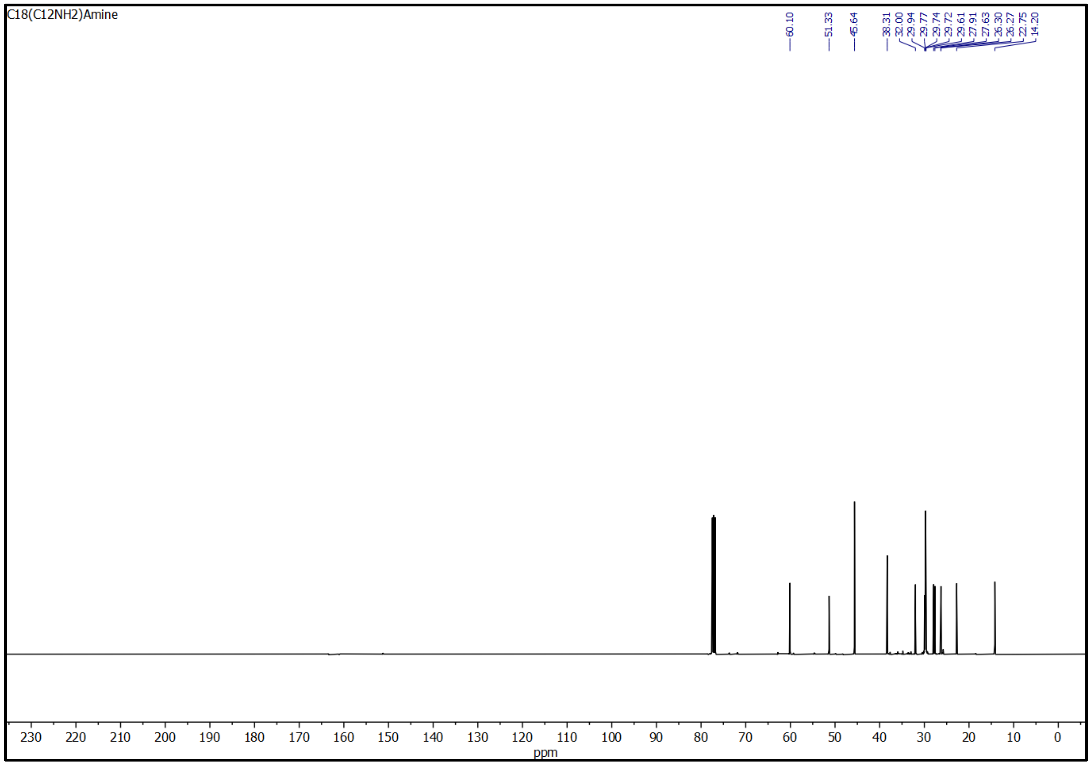

^{13}C NMR of *N,N*-Dimethyloctadecane-1,12-diamine **(9)** in $CDCl_3$.

Compound Table

Label	Tgt Score	Mass Error (ppm)	MFG Formula	Tgt Formula	Obs. RT	Ref. Mass	Obs. Mass
Cpd 1: C20 H44 N2	96.99	1.36	C20 H44 N2	C20 H44 N2	0.218	312.3504	312.3509

Obs. m/z	Obs. RT	Obs. Mass	Tgt Formula	Tgt Mass	Tgt Mass Error	Find Cpds Algorith
313.3584	0.218	312.3509	C20 H44 N2	312.3504	1.36	Find By Formula

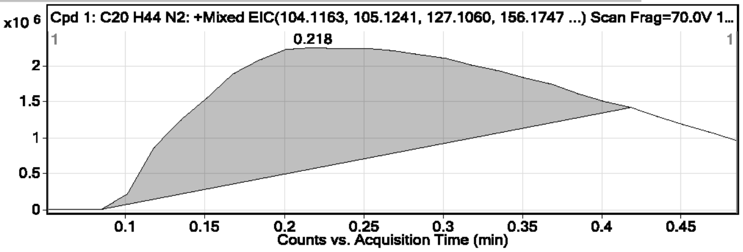

MS Zoomed Spectrum

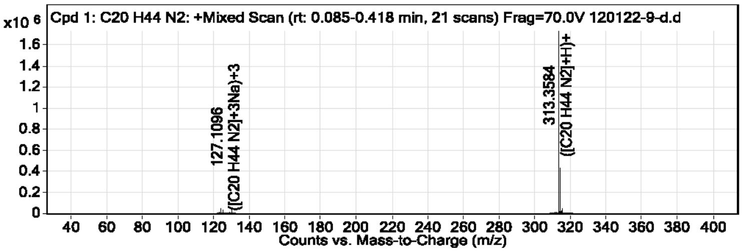

MS Spectrum Peak List

Obs. m/z	Charge	Abund	Formula	Ion/Isotope	Tgt Mass Error (ppm)
127.1096	3	3601.81	C20 H44 N2	(M+3Na)+3	-27.82
127.4434	3	91.54	C20 H44 N2	(M+3Na)+3	-23.12
313.3584	1	1758028.6	C20 H44 N2	(M+H)+	-2.23
314.3607	1	437044.9	C20 H44 N2	(M+H)+	0.65
315.3612	1	46208.69	C20 H44 N2	(M+H)+	9.43
316.354	1	3502.41	C20 H44 N2	(M+H)+	42.18

--- End Of Report ---

HRMS of *N,N*-Dimethyloctadecane-1,12-diamine **(9)**.

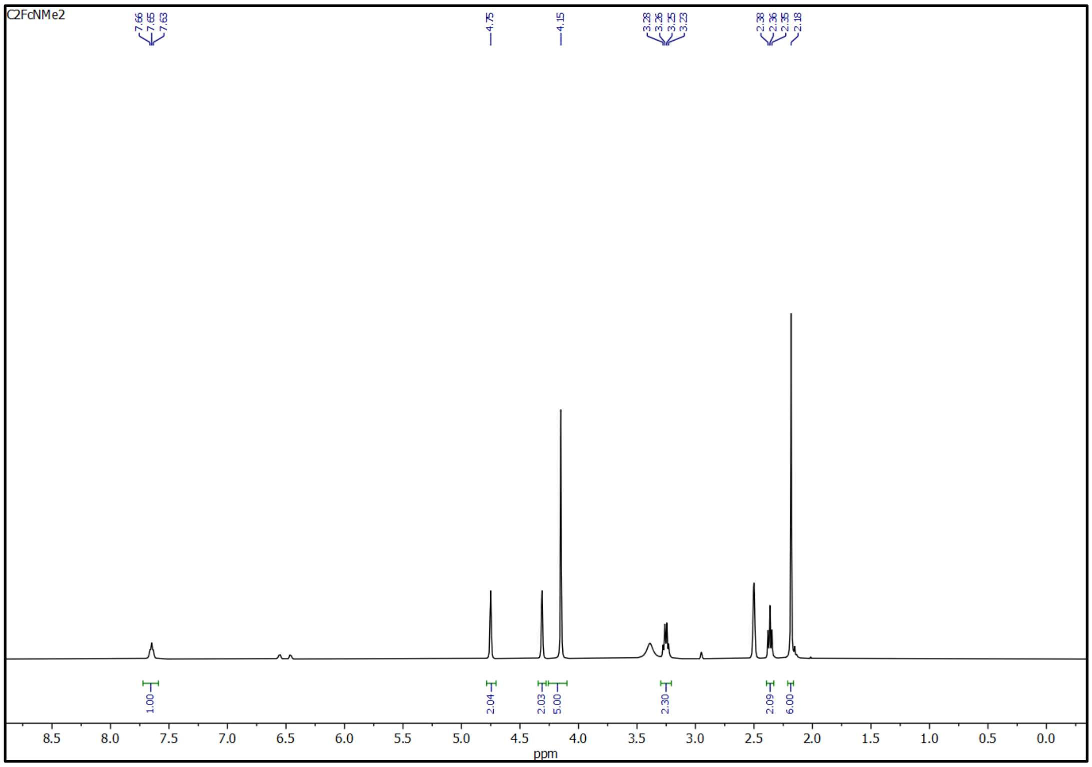

^{1}H NMR of *N*-(2-(dimethylamino)ethyl)ferrocenamide **(10)** in DMSO-d_6.

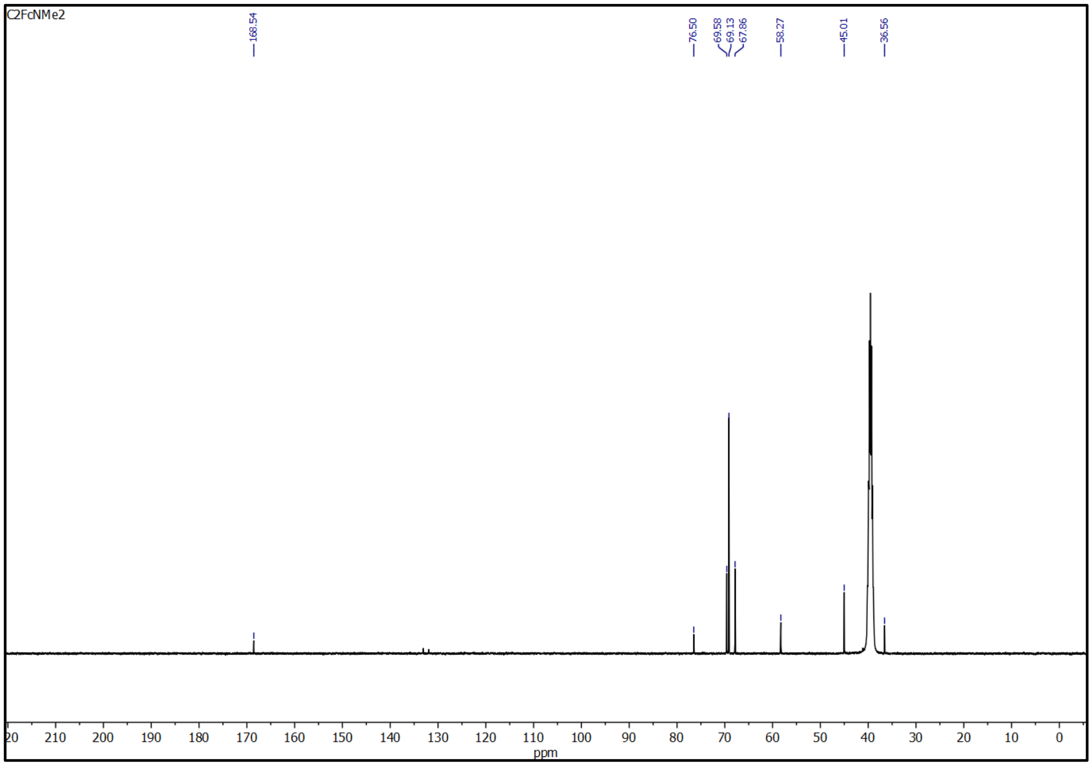

^{13}C NMR of *N*-(2-(dimethylamino)ethyl)ferrocenamide **(10)** in DMSO-d_6.

Compound Table

Label	Tgt Score	Mass Error (ppm)	Tgt Formula	Obs. RT	Ref. Mass	Obs. Mass
Cpd 1: C15 H20 Fe N2 O	69.29	-0.83	C15 H20 Fe N2 O	0.141	298.0972	298.0969

Obs. m/z	Obs. RT	Obs. Mass	Tgt Formula	Tgt Mass	Tgt Mass Error	Find Cpds Algorith
301.0995	0.141	298.0969	C15 H20 Fe N2 O	298.0972	-0.83	Find By Formula

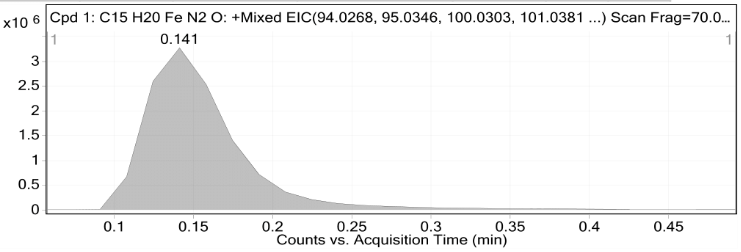

MS Zoomed Spectrum

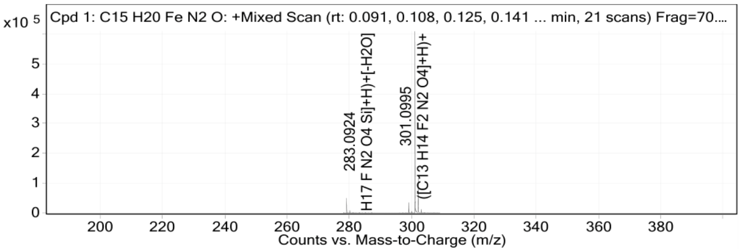

MS Spectrum Peak List

Obs. m/z	Charge	Abund	Formula	Ion/Isotope	Tgt Mass Error (ppm)
283.0924	1	802.06	C12 H17 F N2 O4 Si	(M+H)+[-H2O]	-5.49
301.0995	1	617842.08	C13 H14 F2 N2 O4	(M+H)+	-0.25
302.1025	1	118958.92	C13 H14 F2 N2 O4	(M+H)+	0.04
303.1037	1	12693.04	C13 H14 F2 N2 O4	(M+H)+	3.47
304.1053	1	1053.69	C13 H14 F2 N2 O4	(M+H)+	6.36

--- End Of Report ---

HRMS of *N*-(2-(dimethylamino)ethyl)ferrocenamide **(10)**.

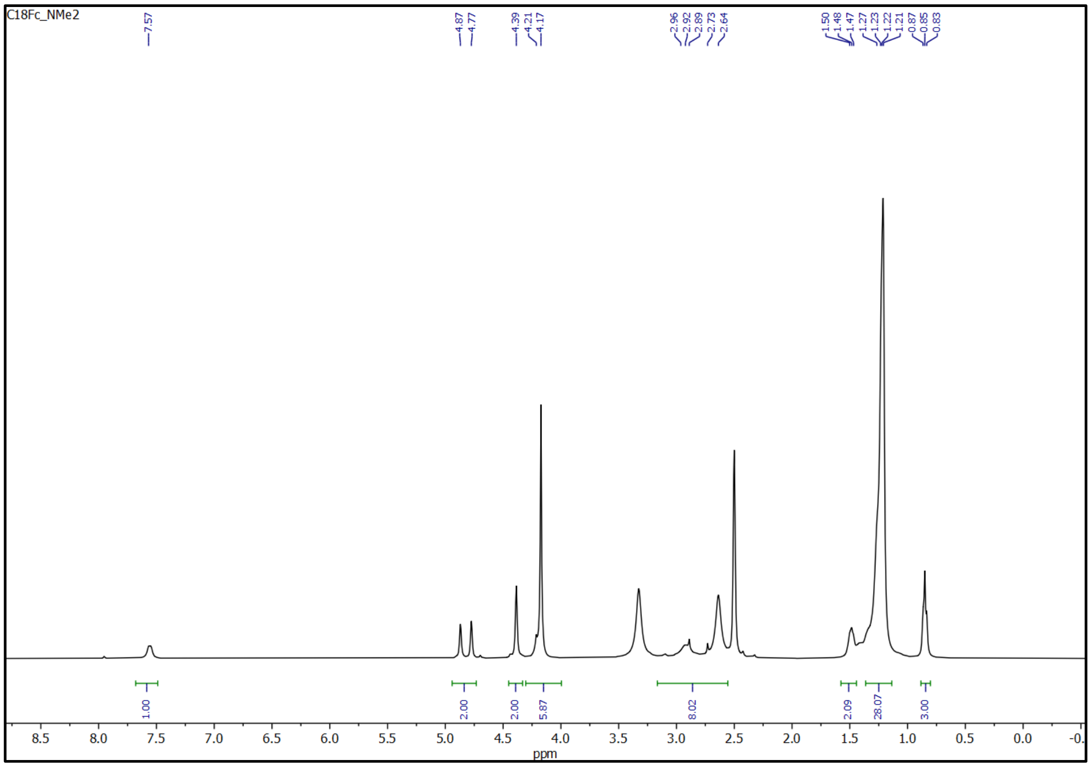

^{1}H NMR of *N*-(1-(dimethylamino)octadecan-2-yl)ferrocenamide **(11)** in DMSO-d_6.

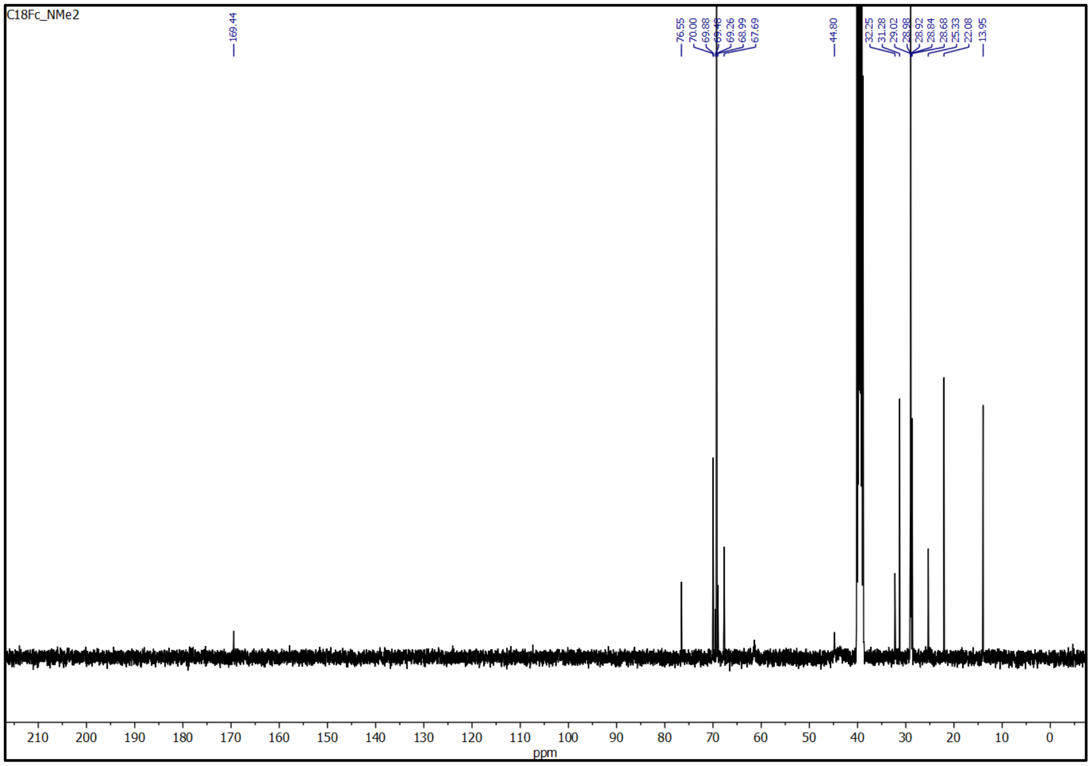

^{13}C NMR of *N*-(1-(dimethylamino)octadecan-2-yl)ferrocenamide **(11)** in DMSO-d_6.

Compound Table

Label	Tgt Score	Mass Error (ppm)	Tgt Formula	Obs. RT	Ref. Mass	Obs. Mass
Cpd 1: C31 H52 Fe N2 O	62.37	-0.9	C31 H52 Fe N2 O	0.122	522.3476	522.3471

Obs. m/z	Obs. RT	Obs. Mass	Tgt Formula	Tgt Mass	Tgt Mass Error	Find Cpds Algorith
525.3507	0.122	522.3471	C31 H52 Fe N2 O	522.3476	-0.9	Find By Formula

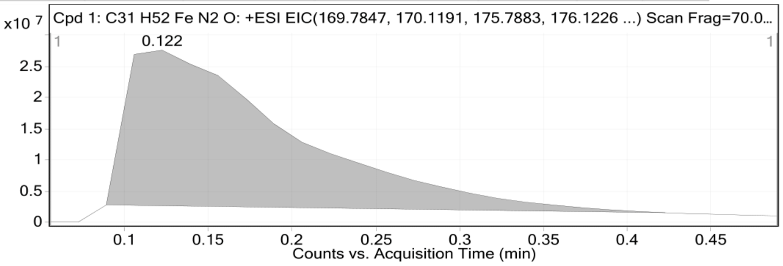

MS Zoomed Spectrum

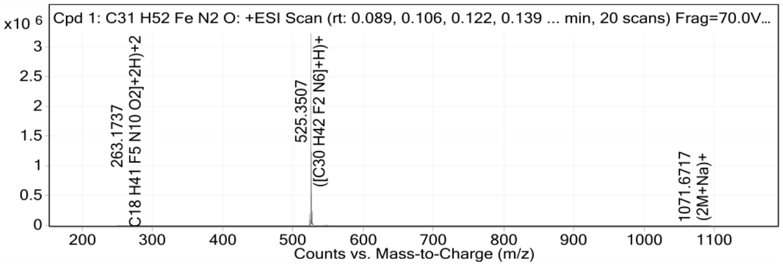

MS Spectrum Peak List

Obs. m/z	Charge	Abund	Formula	Ion/Isotope	Tgt Mass Error (ppm)
263.1737	2	707.29	C18 H41 F5 N10 O2	(M+2H)+2	1.05
523.3548	1	195382.05	C33 H41 N5 O	(M+NH4)+[-H2O]	-0.79
524.3556	1	73366.52	C33 H41 N5 O	(M+NH4)+[-H2O]	3.41
525.3507	1	3235290.04	C30 H42 F2 N6	(M+H)+	0.84
526.3536	1	1243836.95	C30 H42 F2 N6	(M+H)+	1.02
527.3565	1	243394.19	C30 H42 F2 N6	(M+H)+	1.31
528.3584	1	27148.12	C30 H42 F2 N6	(M+H)+	3.38
529.3647	1	2594.44	C30 H42 F2 N6	(M+H)+	-3.1
547.3308	1	19873.74	C31 H48 N2 O3 Si	(M+Na)+	3.34
1071.6717	1	3126.99		(2M+Na)+	3.43

--- End Of Report ---

HRMS of *N*-(1-(dimethylamino)octadecan-2-yl)ferrocenamide **(11)**.

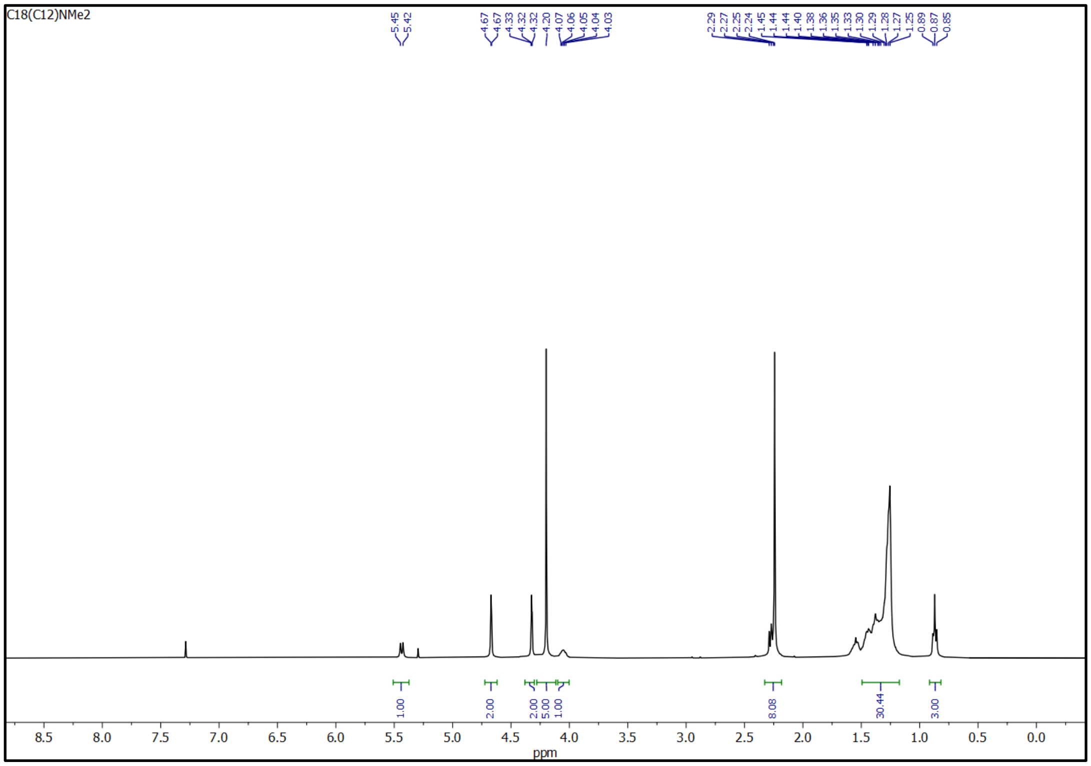

^{1}H NMR of *N*-(1-(dimethylamino)octadecan-12-yl)ferrocenamide **(12)** in $CDCl_3$.

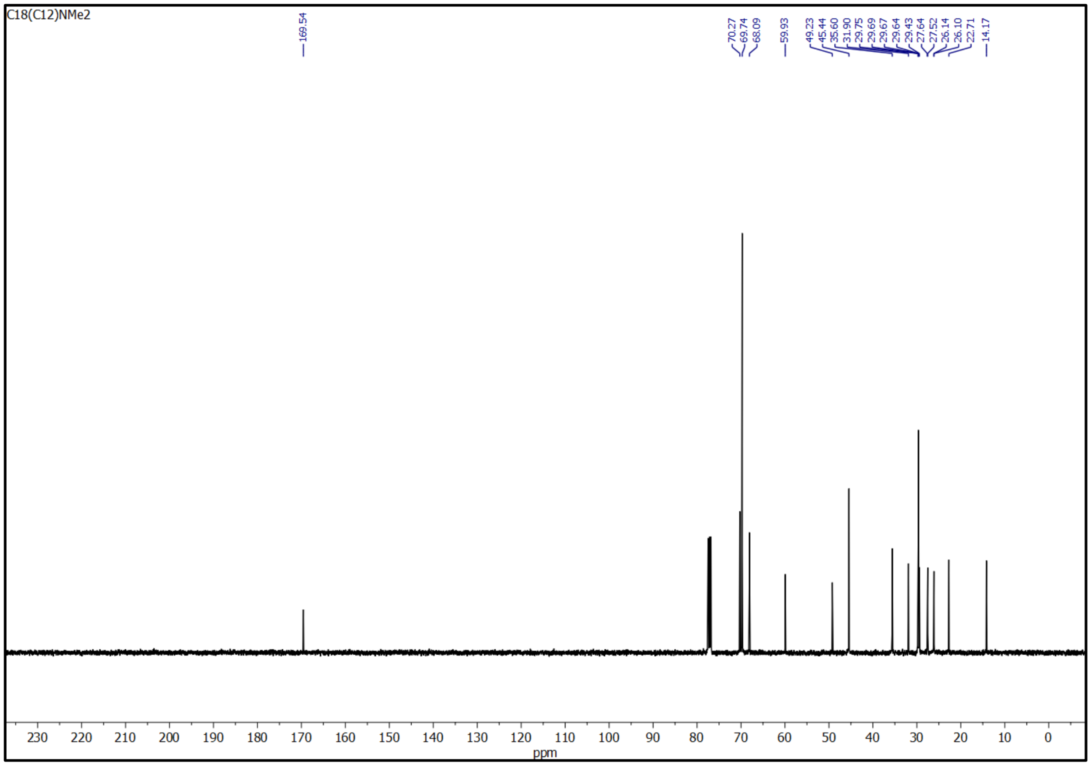

^{13}C NMR of *N*-(1-(dimethylamino)octadecan-12-yl)ferrocenamide **(12)** in $CDCl_3$.

Compound Table

Label	Tgt Score	Mass Error (ppm)	Tgt Formula	Obs. RT	Ref. Mass	Obs. Mass
Cpd 1: C31 H52 Fe N2 O	62.7	1.11	C31 H52 Fe N2 O	0.138	522.3476	522.3482

Obs. m/z	Obs. RT	Obs. Mass	Tgt Formula	Tgt Mass	Tgt Mass Error	Find Cpds Algorith
525.3505	0.138	522.3482	C31 H52 Fe N2 O	522.3476	1.11	Find By Formula

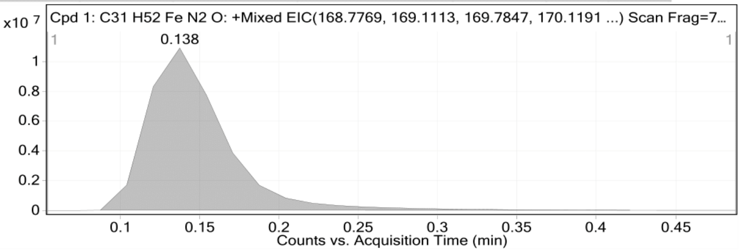

MS Zoomed Spectrum

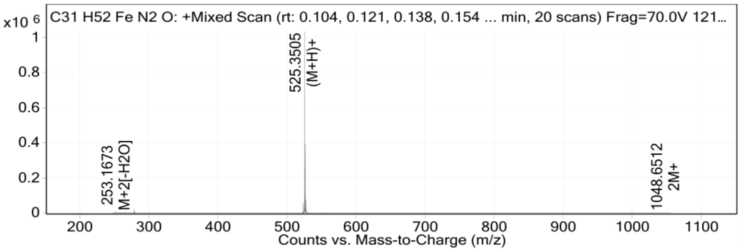

MS Spectrum Peak List

Obs. m/z	Charge	Abund	Ion/Isotope	Tgt Mass Error (ppm)
253.1673	2	1456.36	M+2[-H2O]	-6.52
263.1707	2	1435.98	(M+2H)+2	30.65
276.1643	2	304	(M+2Na)+2[-H2O]	-32.11
523.3542	1	62183.63	M+	-7.39
524.3566	1	20692.94	M+	-27.09
525.3505	1	1043762.71	(M+H)+	-0.57
526.3535	1	395194.09	(M+H)+	-0.42
527.3563	1	77841.87	(M+H)+	-0.58
528.3587	1	8395.25	(M+H)+	-0.33
529.3603	1	805.95	(M+H)+	1.06

--- End Of Report ---

HRMS of *N*-(1-(dimethylamino)octadecan-12-yl)ferrocenamide **(12)**.

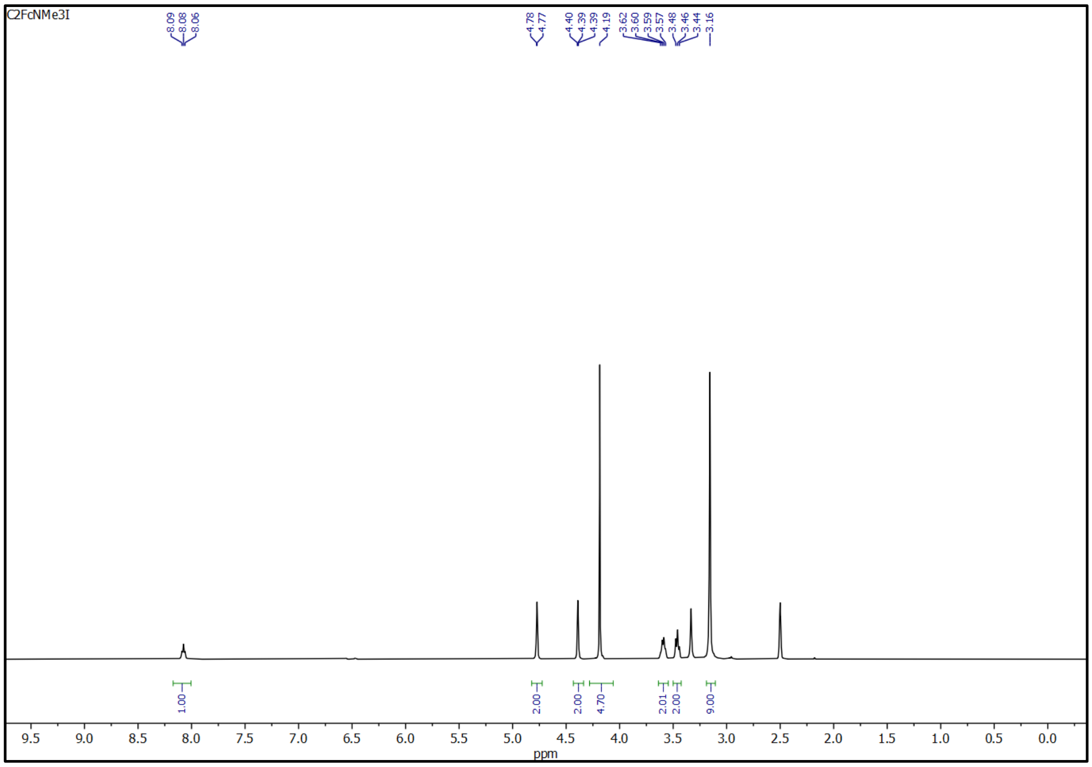

^{1}H NMR of *N*-(2-(trimethylaminium iodide)ethyl)ferrocenamide **(13)** in DMSO-d_6.

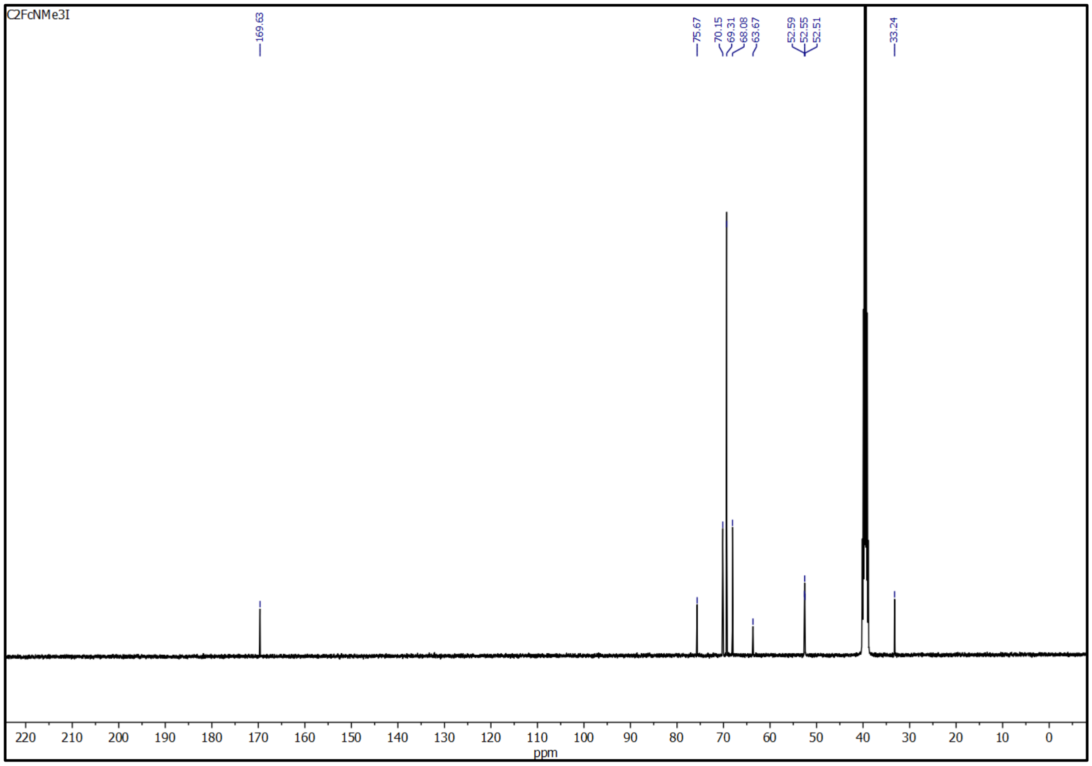

^{13}C NMR of *N*-(2-(trimethylaminium iodide)ethyl)ferrocenamide **(13)** in DMSO-d_6.

Compound Table

Label	Tgt Score	Mass Error (ppm)	Tgt Formula	Obs. RT	Ref. Mass	Obs. Mass
Cpd 1: C16 H23 Fe N2 O	61.89	1.08	C16 H23 Fe N2 O	0.186	313.1207	313.121

Obs. m/z	Obs. RT	Obs. Mass	Tgt Formula	Tgt Mass	Tgt Mass Error	Find Cpds Algorith
315.1159	0.186	313.121	C16 H23 Fe N2 O	313.1207	1.08	Find By Formula

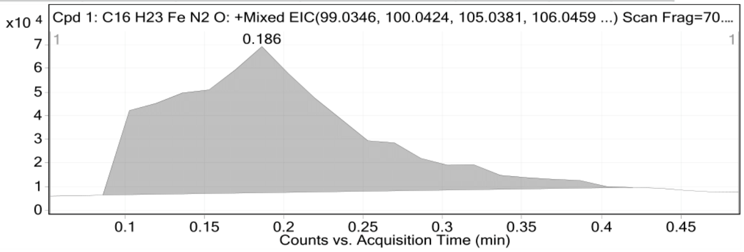

MS Zoomed Spectrum

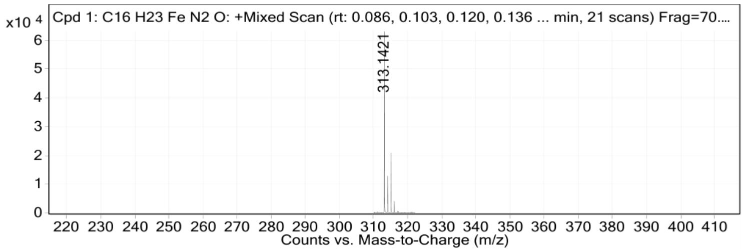

MS Spectrum Peak List

Obs. m/z	Charge	Abund	Formula	Ion/Isotope	Tgt Mass Error (ppm)
313.1421		65807.81			
315.1159	1	21043.28	C15 H13 F2 N6	M+	1.69
316.1174	1	4245.61	C15 H13 F2 N6	M+	5.13
317.1246	1	637.48	C15 H13 F2 N6	M+	-9.39

--- End Of Report ---

HRMS of *N*-(2-(trimethylaminium iodide)ethyl)ferrocenamide **(13)**.

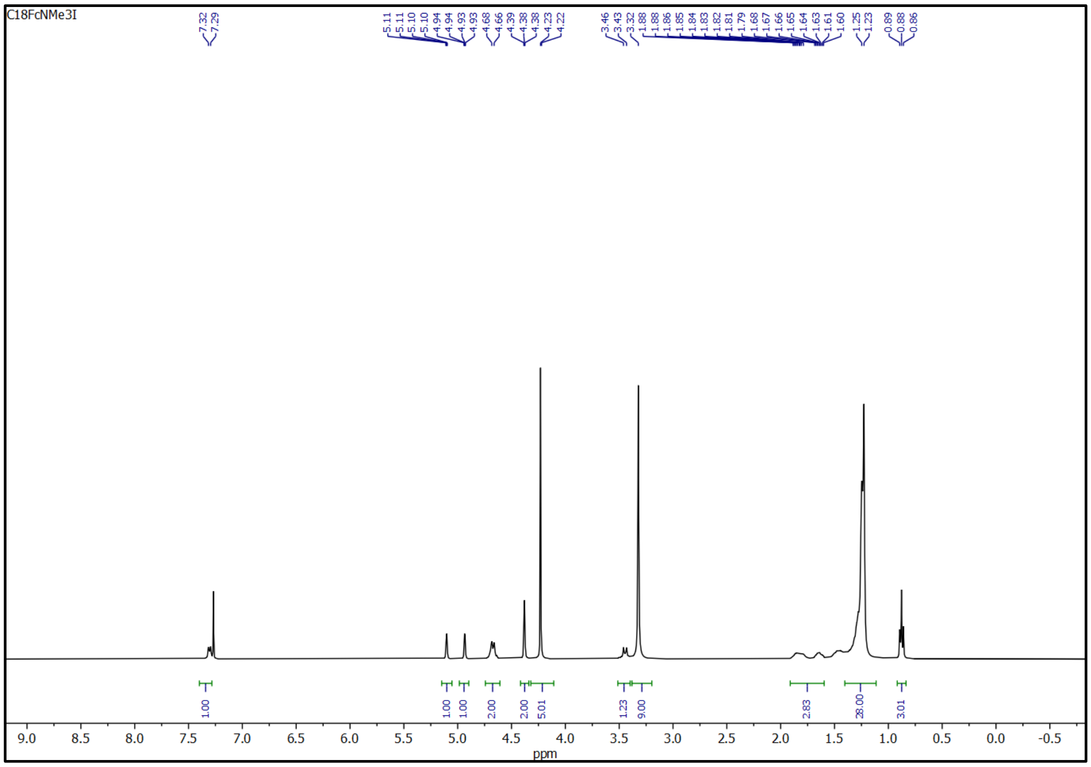

^{1}H NMR of *N*-(1-(trimethylaminium iodide)octadecan-2-yl)ferrocenamide **(14)** in $CDCl_3$.

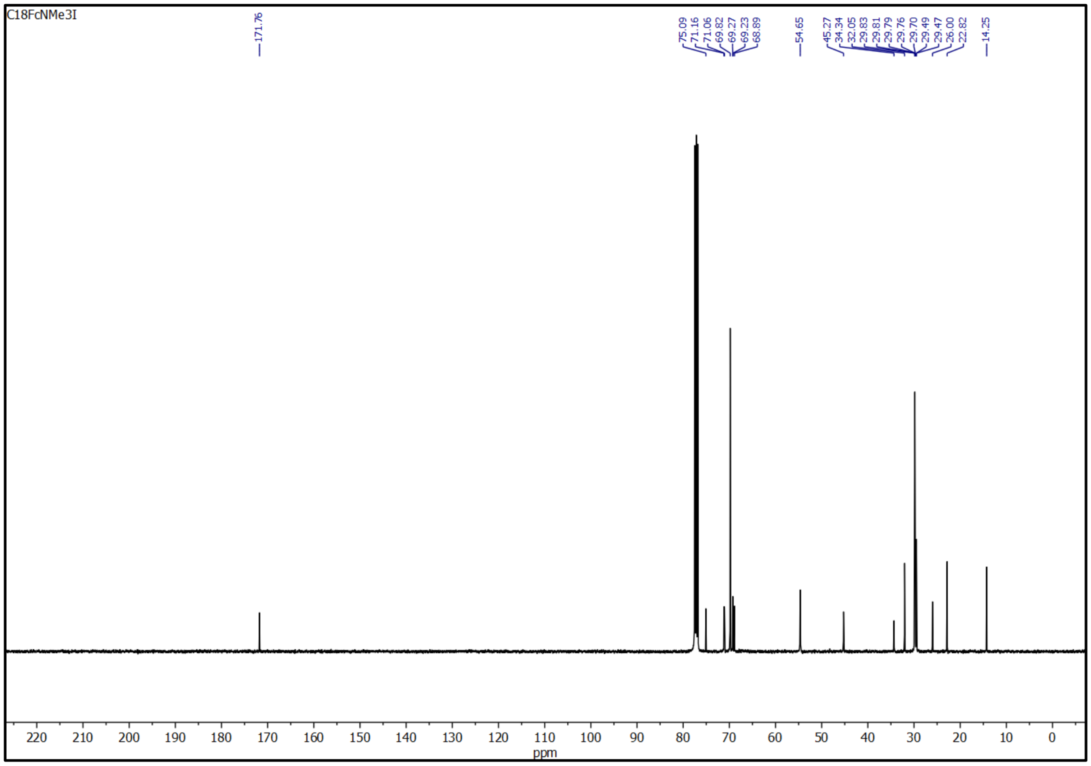

^{13}C NMR of of *N*-(1-(trimethylaminium iodide)octadecan-2-yl)ferrocenamide **(14)** in $CDCl_3$.

Compound Table

Label	Tgt Score	Mass Error (ppm)	Tgt Formula	Obs. RT	Ref. Mass	Obs. Mass
Cpd 1: C32 H55 Fe N2 O	63.65	-0.64	C32 H55 Fe N2 O	0.119	537.3711	537.3707

Obs. m/z	Obs. RT	Obs. Mass	Tgt Formula	Tgt Mass	Tgt Mass Error	Find Cpds Algorith
539.3659	0.119	537.3707	C32 H55 Fe N2 O	537.3711	-0.64	Find By Formula

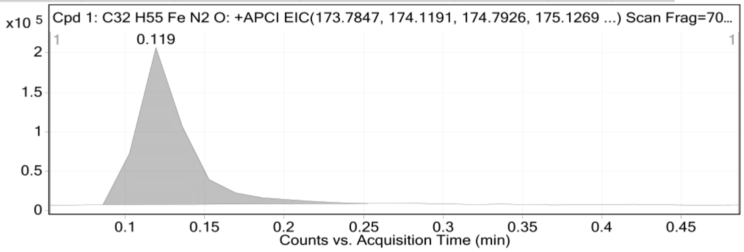

MS Zoomed Spectrum

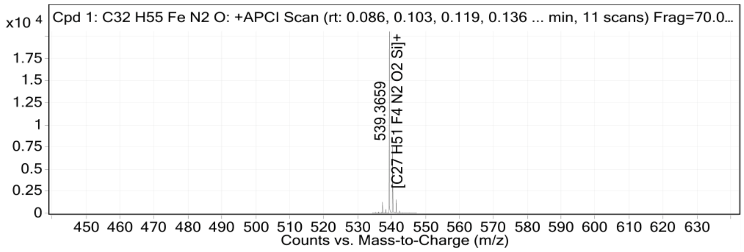

MS Spectrum Peak List

Obs. m/z	Charge	Abund	Formula	Ion/Isotope	Tgt Mass Error (ppm)
539.3659	1	20605.03	C27 H51 F4 N2 O2 Si	M+	-1.58
539.3659		20605.03			
540.3683	1	7558.08	C27 H51 F4 N2 O2 Si	M+	-0.92
541.3691	1	1596.01	C27 H51 F4 N2 O2 Si	M+	-2.72
542.3694	1	281.26	C27 H51 F4 N2 O2 Si	M+	-1.24

--- End Of Report ---

HRMS of *N*-(1-(trimethylaminium iodide)octadecan-2-yl)ferrocenamide **(14)**.

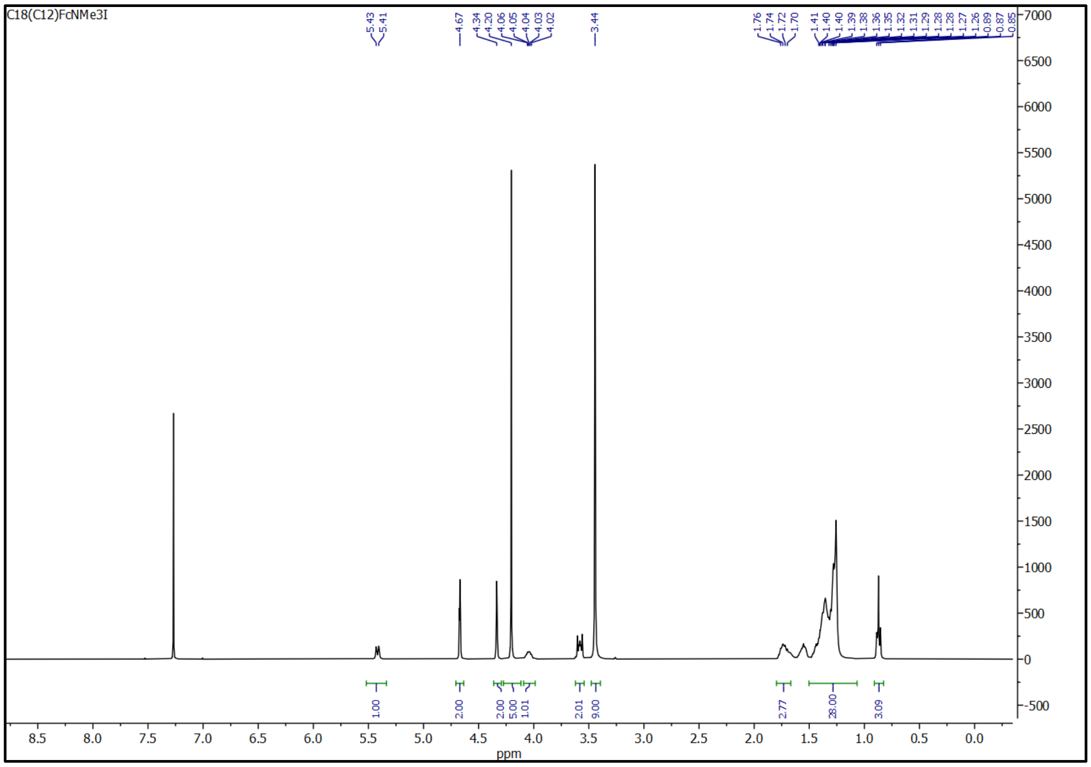

^{1}H NMR of *N*-(1-(trimethylaminium iodide)octadecan-12-yl)ferrocenamide **(15)** in $CDCl_3$.

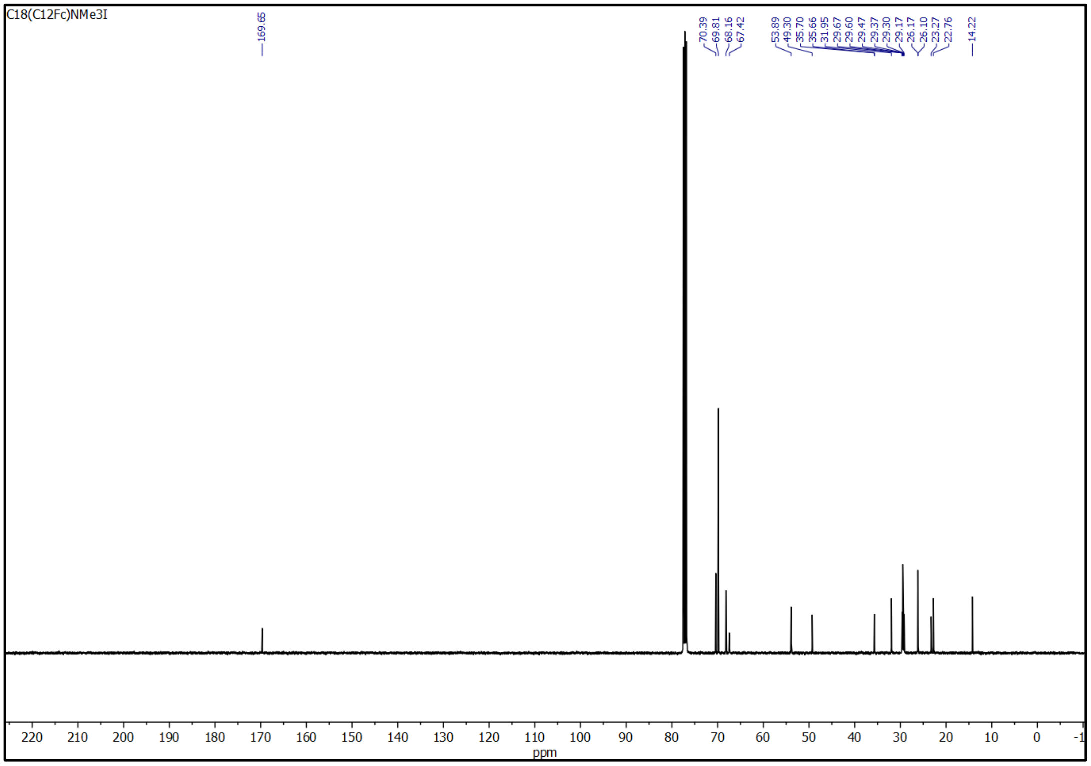

^{13}C NMR of *N*-(1-(trimethylaminium iodide)octadecan-12-yl)ferrocenamide **(15)** in $CDCl_3$.

Compound Table

Label	Tgt Score	Mass Error (ppm)	Tgt Formula	Obs. RT	Ref. Mass	Obs. Mass
Cpd 1: C32 H55 Fe N2 O	44.03	-7.53	C32 H55 Fe N2 O	0.117	537.3711	537.367

Obs. m/z	Obs. RT	Obs. Mass	Tgt Formula	Tgt Mass	Tgt Mass Error	Find Cpds Algorith
539.3635	0.117	537.367	C32 H55 Fe N2 O	537.3711	-7.53	Find By Formula

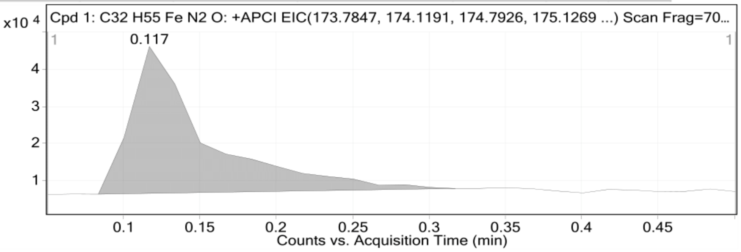

MS Zoomed Spectrum

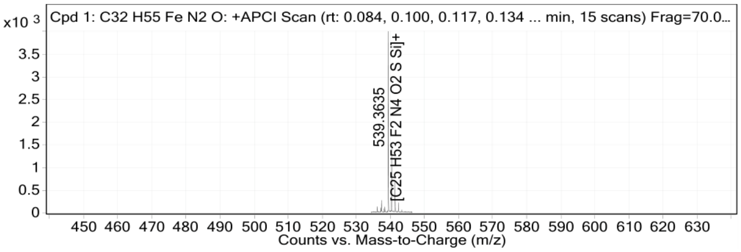

MS Spectrum Peak List

Obs. m/z	Charge	Abund	Formula	Ion/Isotope	Tgt Mass Error (ppm)
539.3635	1	4036.94	C25 H53 F2 N4 O2 S Si	M+	-2.52
540.3666	1	1530.4	C25 H53 F2 N4 O2 S Si	M+	-3.62
541.3545	1	697.21	C25 H53 F2 N4 O2 S Si	M+	14.27

--- End Of Report ---

HRMS of *N*-(1-(trimethylaminium iodide)octadecan-12-yl)ferrocenamide **(15)**.

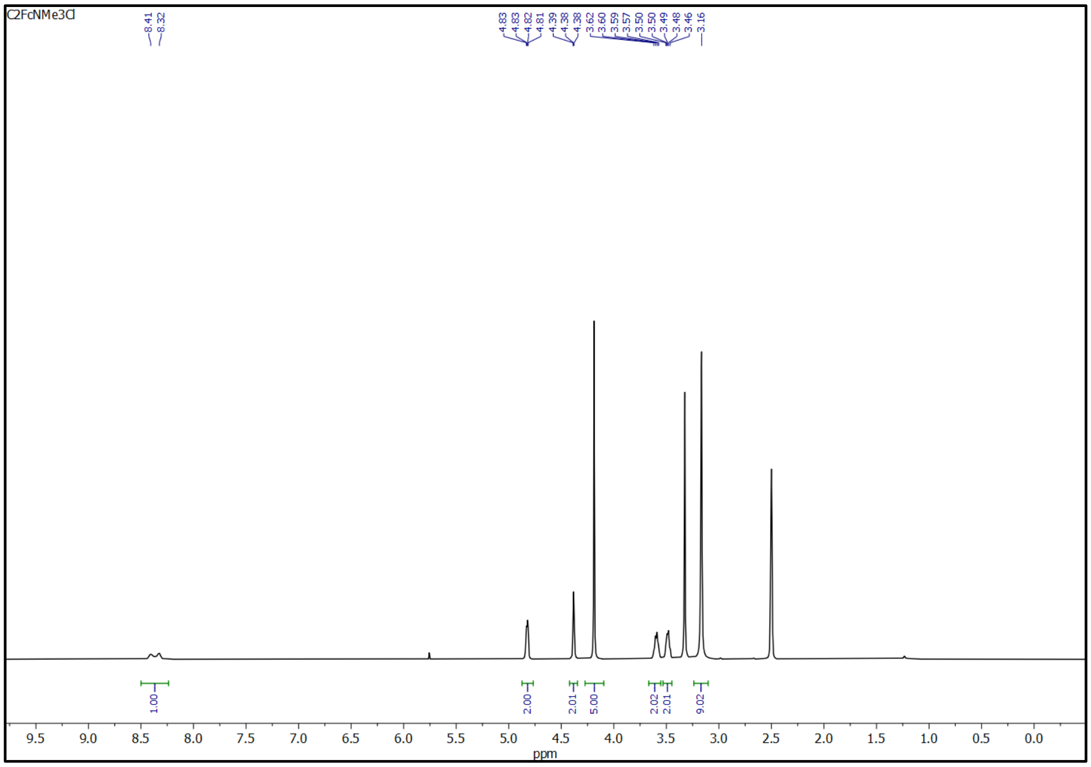

^{1}H NMR of *N*-(2-(trimethylaminium chloride)ethyl)ferrocenamide **(16)** in DMSO-d_6.

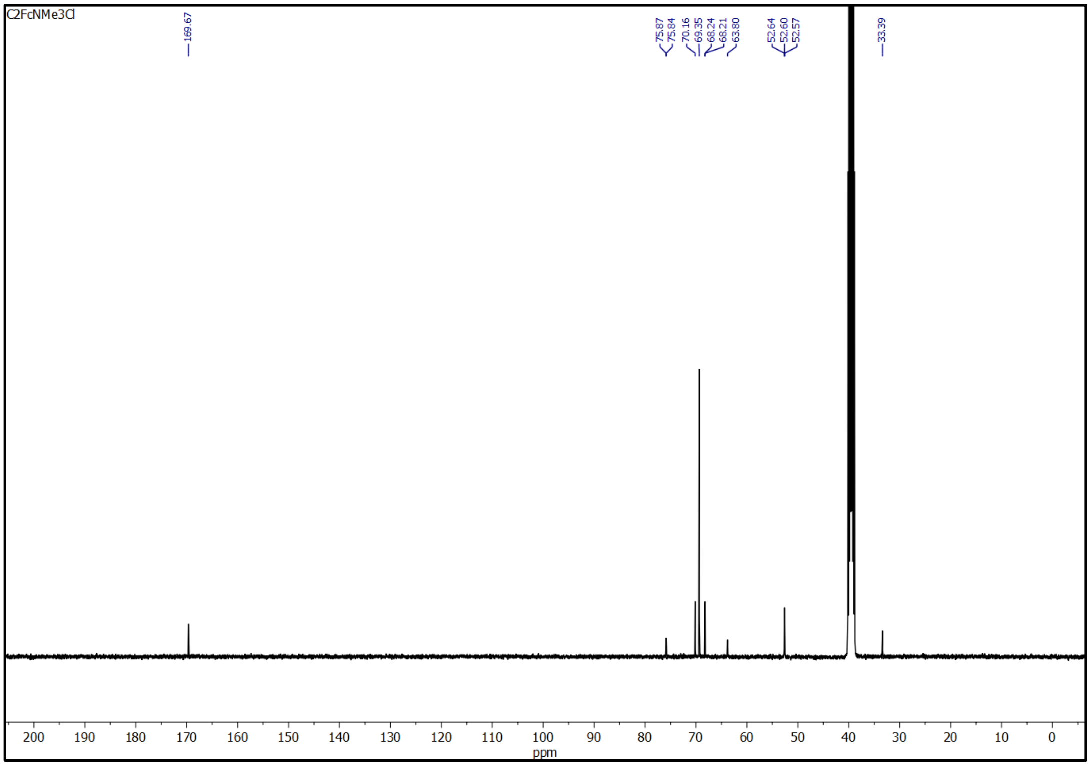

^{13}C NMR of *N*-(2-(trimethylaminium chloride)ethyl)ferrocenamide **(16)** in DMSO-d_6.

Compound Table

Label	Tgt Score	Mass Error (ppm)	Tgt Formula	Obs. RT	Ref. Mass	Obs. Mass
Cpd 1: C16 H23 Fe N2 O	69.11	-0.24	C16 H23 Fe N2 O	0.123	313.1207	313.1206

Obs. m/z	Obs. RT	Obs. Mass	Tgt Formula	Tgt Mass	Tgt Mass Error	Find Cpds Algorith
315.1154	0.123	313.1206	C16 H23 Fe N2 O	313.1207	-0.24	Find By Formula

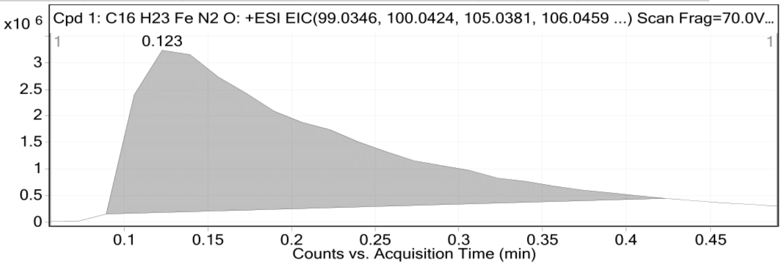

MS Zoomed Spectrum

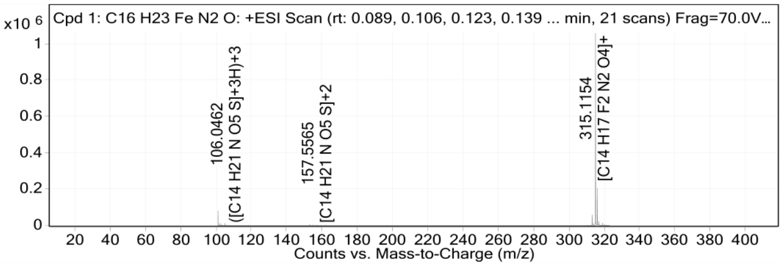

MS Spectrum Peak List

Obs. m/z	Charge	Abund	Formula	Ion/Isotope	Tgt Mass Error (ppm)
106.0462	3	647.8	C14 H21 N O5 S	(M+3H)+3	-8.81
157.5565	2	276.38	C14 H21 N O5 S	M+2	0.02
315.1154	1	1059192.26	C14 H17 F2 N2 O4	M+	-1.04
316.118	1	210710.6	C14 H17 F2 N2 O4	M+	0.57
317.1195	1	22574.3	C14 H17 F2 N2 O4	M+	3.13
318.121	1	1820.07	C14 H17 F2 N2 O4	M+	6.35

--- End Of Report ---

HRMS of *N*-(2-(trimethylaminium chloride)ethyl)ferrocenamide **(16)**.

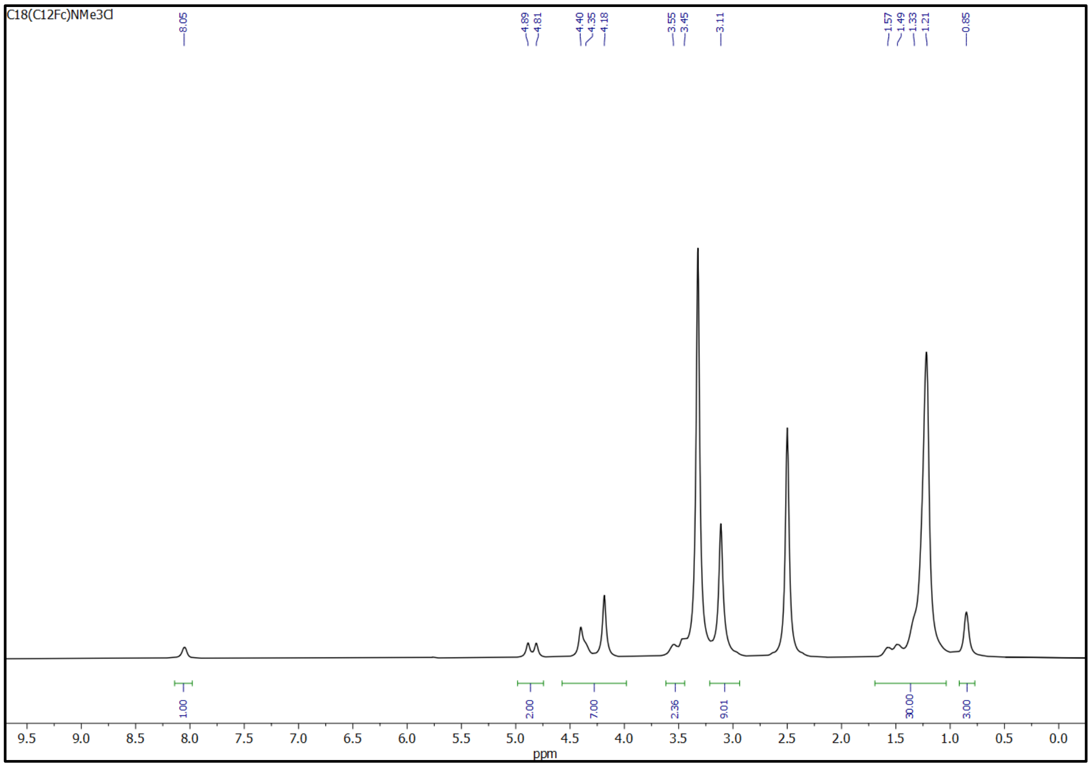

^{1}H NMR of *N*-(1-(trimethylaminium chloride)octadecan-2-yl)ferrocenamide **(17)** in DMSO-d$_6$.

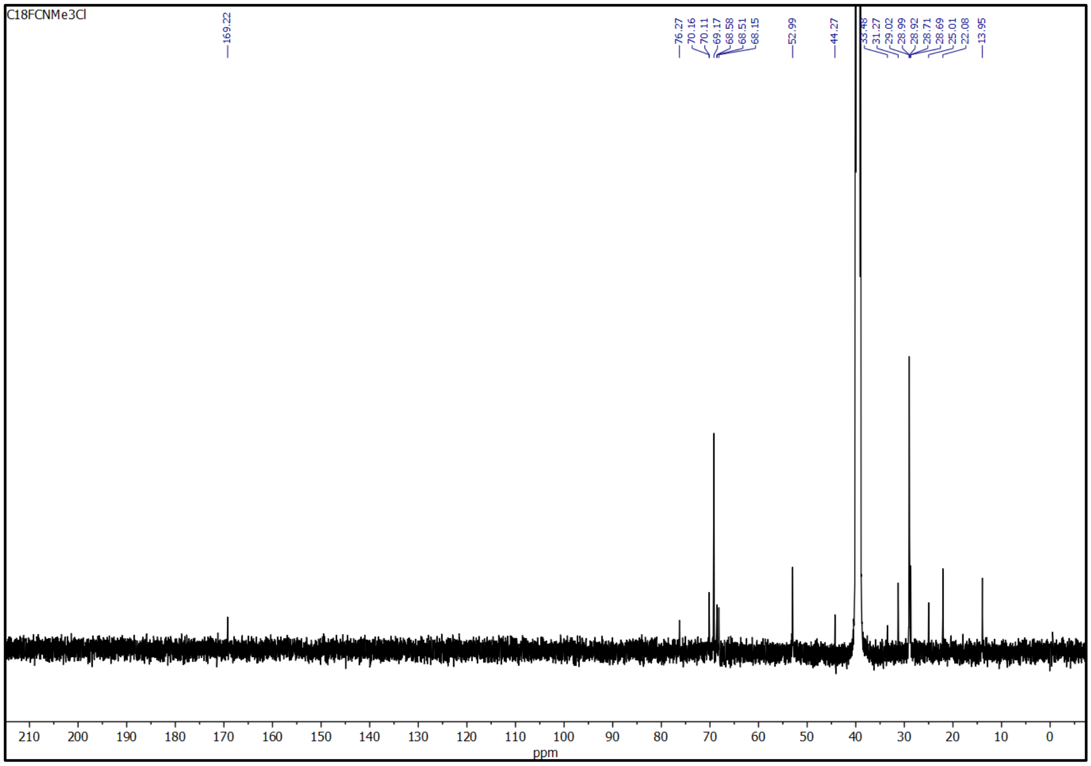

^{13}C NMR of *N*-(1-(trimethylaminium chloride)octadecan-2-yl)ferrocenamide **(17)** in DMSO-d$_6$.

Compound Table

Label	Tgt Score	Mass Error (ppm)	Tgt Formula	Obs. RT	Ref. Mass	Obs. Mass
Cpd 1: C32 H55 Fe N2 O	63.02	-1.47	C32 H55 Fe N2 O	0.13	537.3711	537.3703

Obs. m/z	Obs. RT	Obs. Mass	Tgt Formula	Tgt Mass	Tgt Mass Error	Find Cpds Algorith
539.3653	0.13	537.3703	C32 H55 Fe N2 O	537.3711	-1.47	Find By Formula

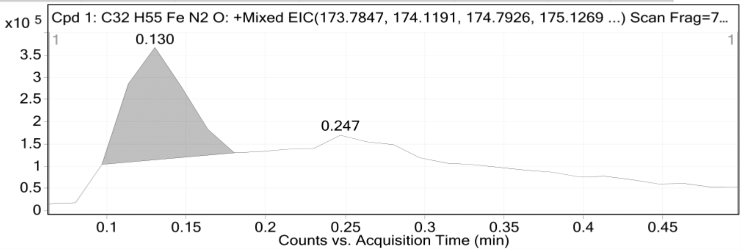

MS Zoomed Spectrum

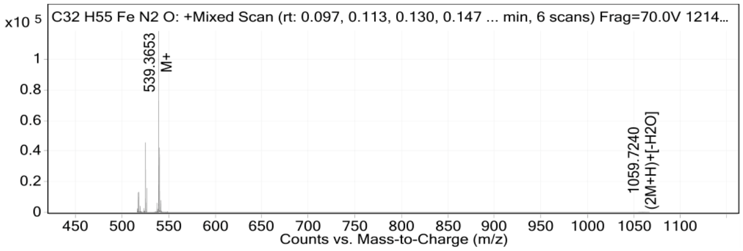

MS Spectrum Peak List

Obs. m/z	Charge	Abund	Ion/Isotope	Tgt Mass Error (ppm)
521.3567	1	123.15	M+[-H2O]	-2.63
539.3653	1	119566.49	M+	1.06
539.3653		119566.49		
540.3678	1	42341.65	M+	2.07
541.3695	1	7990.28	M+	4.05
542.3707	1	914.45	M+	6.53
544.3464	1	138.58	(M+Na)+[-H2O]	-2.33
1059.724	1	218.19	(2M+H)+[-H2O]	9.7
1060.7368	1	127.67	(2M+H)+[-H2O]	0.5
1061.7527	1	107.43	(2M+H)+[-H2O]	-21.53

--- End Of Report ---

HRMS of *N*-(1-(trimethylaminium chloride)octadecan-2-yl)ferrocenamide **(17)**.

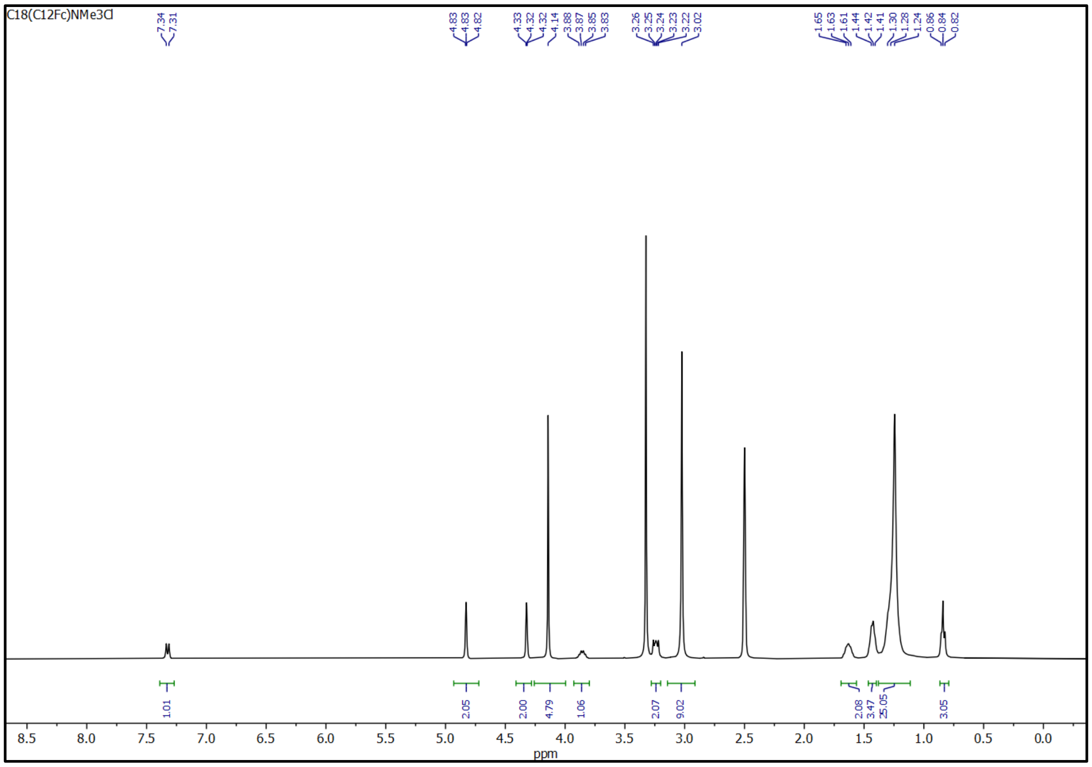

^{1}H NMR of *N*-(1-(trimethylaminium chloride)octadecan-12-yl)ferrocenamide **(18)** in DMSO-d$_6$.

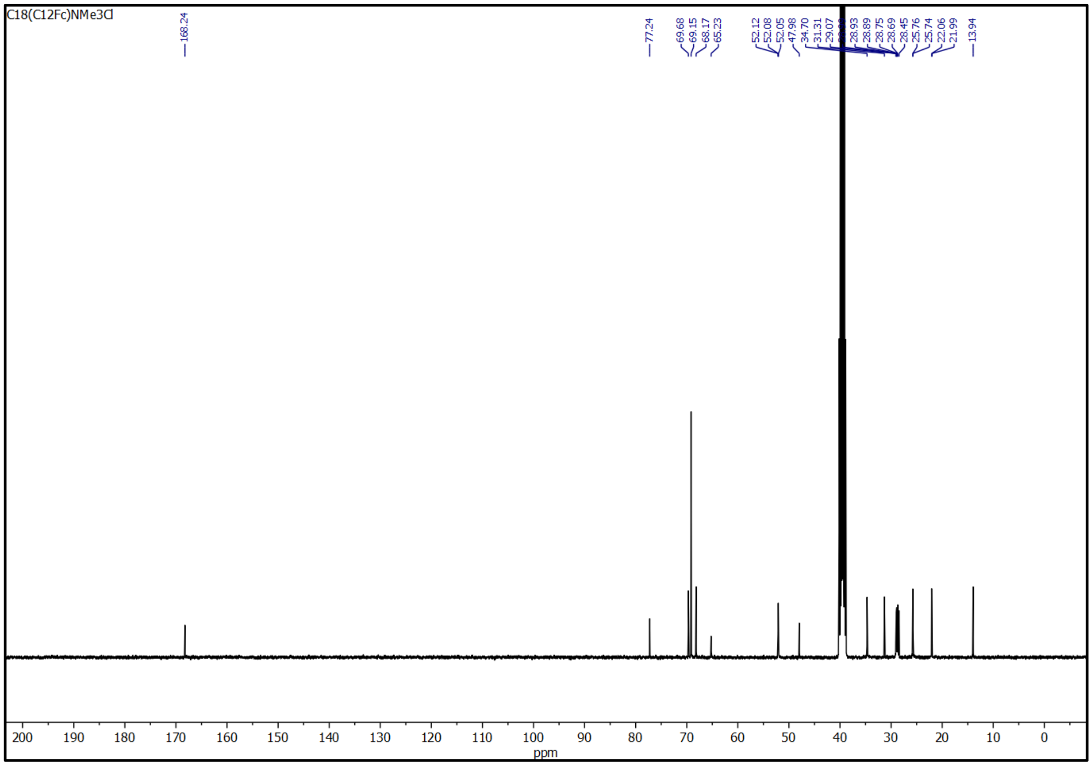

^{13}C NMR of *N*-(1-(trimethylaminium chloride)octadecan-12-yl)ferrocenamide **(18)** in DMSO-d$_6$.

Compound Table

Label	Tgt Score	Mass Error (ppm)	Tgt Formula	Obs. RT	Ref. Mass	Obs. Mass
Cpd 1: C32 H55 Fe N2 O	62.55	-1.56	C32 H55 Fe N2 O	0.117	537.3711	537.3702

Obs. m/z	Obs. RT	Obs. Mass	Tgt Formula	Tgt Mass	Tgt Mass Error	Find Cpds Algorith
539.3661	0.117	537.3702	C32 H55 Fe N2 O	537.3711	-1.56	Find By Formula

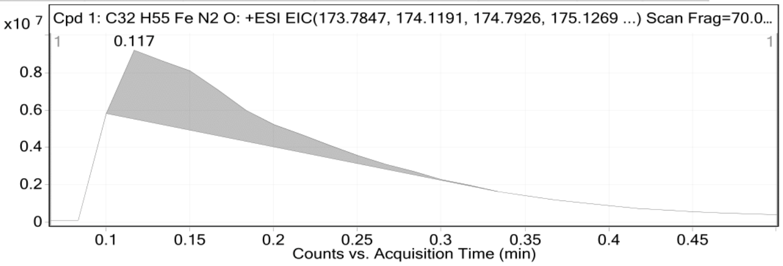

MS Zoomed Spectrum

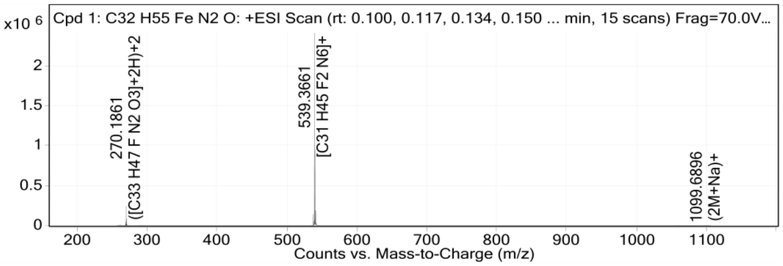

MS Spectrum Peak List

Obs. m/z	Charge	Abund	Formula	Ion/Isotope	Tgt Mass Error (ppm)
269.6825	2	46937.51	C27 H58 F N O2 S2 Si	M+2	0.1
270.1861	2	240275.27	C33 H47 F N2 O3	(M+2H)+2	-1.12
270.6874	2	86422.18	C33 H47 F N2 O3	(M+2H)+2	0.28
271.1885	2	17043.18	C33 H47 F N2 O3	(M+2H)+2	1.68
271.689	2	2275.72	C33 H47 F N2 O3	(M+2H)+2	5.41
539.3661	1	2434039.52	C31 H45 F2 N6	M+	1.28
540.3697	1	947302.43	C31 H45 F2 N6	M+	0.3
541.3719	1	187597.61	C31 H45 F2 N6	M+	1.81
542.3732	1	20711.17	C31 H45 F2 N6	M+	4.86
1099.6896	1	248.95		(2M+Na)+	33.77

--- End Of Report ---

HRMS of *N*-(1-(trimethylaminium chloride)octadecan-12-yl)ferrocenamide **(18)**.

References

(1) Wan, L. J.; Terashima, M.; Noda, H.; Osawa, M. Molecular Orientation and Ordered Structure of Benzenethiol Adsorbed on Gold(111). *J. Phys. Chem. B* **2000**, *104*, 3563–3569.

(2) Awad, M. I.; El-Deab, M. S.; Ohsaka, T. Tailor-Designed Platinum Nanoparticles Electrodeposited onto Gold Electrode. *J. Electrochem. Soc.* **2007**, *154*, B810.

(3) Cuharuc, A. S.; Zhang, G.; Unwin, P. R. Electrochemistry of Ferrocene Derivatives on Highly Oriented Pyrolytic Graphite (HOPG): Quantification and Impacts of Surface Adsorption. *Phys. Chem. Chem. Phys* **2016**, *18*, 4966–4977.

(4) Patel, A. N.; Collignon, M. G.; O'Connell, M. A.; Hung, W. O. Y.; McKelvey, K.; Macpherson, J. V.; Unwin, P. R. A New View of Electrochemistry at Highly Oriented Pyrolytic Graphite. *J. Am. Chem. Soc.* **2012**, *134*, 20117–20130.

(5) Wang, Y.; Laborda, E.; Ward, K. R.; Tschulik, K.; Compton, R. G. A Kinetic Study of Oxygen Reduction Reaction and Characterization on Electrodeposited Gold Nanoparticles of Diameter between 17 nm and 40 nm in 0.5 M Sulfuric Acid. *Nanoscale* **2013**, *5*, 9699–9708.

(6) Marshall-Roth, T.; Libretto, N. J.; Wrobel, A. T.; Anderton, K. J.; Pegis, M. L.; Ricke, N. D.; Voorhis, T. Van; Miller, J. T.; Surendranath, Y. A Pyridinic Fe-N_4 Macrocycle Models the Active Sites in Fe/N-Doped Carbon Electrocatalysts. *Nat. Commun.* **2020**, *11*, 1–14.

(7) Wuttig, A.; Ryu, J.; Surendranath, Y. Electrolyte Competition Controls Surface Binding of CO Intermediates to CO_2 Reduction Catalysts. *J. Phys. Chem. C* **2021**, *125*, 17042–17050.

(8) Yaguchi, M.; Uchida, T.; Motobayashi, K.; Osawa, M. Speciation of Adsorbed Phosphate at Gold Electrodes: A Combined Surface-Enhanced Infrared Absorption Spectroscopy and DFT Study. *J. Phys. Chem. Lett.* **2016**, *7*, 3097–3102.

(9) Wuttig, A.; Yaguchi, M.; Motobayashi, K.; Osawa, M.; Surendranath, Y. Inhibited Proton Transfer Enhances Au-Catalyzed CO_2-to-Fuels Selectivity. *Proc. Natl. Acad. Sci. U. S. A.* **2016**, *113*, E4585–E4593.

(10) Latouche, C.; Palazzetti, F.; Skouteris, D.; Barone, V. High-Accuracy Vibrational Computations for Transition-Metal Complexes Including Anharmonic Corrections: Ferrocene, Ruthenocene, and Osmocene as Test Cases. *J. Chem. Theory Comput.* **2014**, *10*, 4565–4573.

(11) Randles, J. E. B. A Cathode Ray Polarograph. Part II.—The Current-Voltage Curves. *Trans. Faraday Soc.* **1948**, *44*, 327–338.

(12) Ševčík, A. Oscillographic Polarography with Periodical Triangular Voltage. *Collect. Czech. Chem. Commun.* **1948**, *13*, 349–377.

(13) Elgrishi, N.; Rountree, K. J.; McCarthy, B. D.; Rountree, E. S.; Eisenhart, T. T.; Dempsey, J. L. A Practical Beginner's Guide to Cyclic Voltammetry. *J. Chem. Educ.* **2018**, *95*, 197–206.

(14) Janz, G. J.; Oliver, B. G.; Lakshminarayanan, G. R.; Mayer, G. E. Electrical Conductance, Diffusion, Viscosity, and Density of Sodium Nitrate, Sodium Perchlorate, and Sodium Thiocyanate in Concentrated Aqueous Solutions. *J. Phys. Chem.* **1970**, *74*, 1285–1289.

(15) Hnedkovsky, L.; Hefter, G. Densities and Apparent Molar Volumes of Aqueous Solutions of $NaClO_4$, $KClO_4$, and KCl at Temperatures from 293 to 343 K. *J. Chem. Eng. Data* **2021**, *66*, 3645–3658.

(16) Popenoe, D. D.; Deinhammer, R. S.; Porter, M. D. Infrared Spectroelectrochemical Characterization of Ferrocene-Terminated Alkanethiolate Monolayers at Gold. *Langmuir* **1992**, *8*, 2521–2530.

(17) Culbertson, S. M.; Porter, N. A. Unsymmetrical Azo Initiators Increase Efficiency of Radical Generation in Aqueous Dispersions, Liposomal Membranes, and Lipoproteins. *J. Am. Chem. Soc.* **2000**, *122*, 4032–4038.

(18) Abraham, S.; Lan, Y.; Lam, R. S. H.; Grahame, D. A. S.; Kim, J. J. H.; Weiss, R. G.; Rogers, M. A. Influence of Positional Isomers on the Macroscale and Nanoscale Architectures of Aggregates of Racemic Hydroxyoctadecanoic Acids in Their Molecular Gel, Dispersion, and Solid States. *Langmuir* **2012**, *28*, 4955–4964.

(19) Yearick, K.; Ekoue-Kovi, K.; Iwaniuk, D. P.; Natarajan, J. K.; Alumasa, J.; De Dios, A. C.; Roepe, P. D.; Wolf, C. Overcoming Drug Resistance to Heme-Targeted Antimalarials by Systematic Side Chain Variation of 7-Chloro-4-Aminoquinolines. *J. Med. Chem.* **2008**, *51*, 1995–1998.

(20) MacPhail, R. A.; Strauss, H. L.; Snyder, R. G.; Elliger, C. A. C-H Stretching Modes and the Structure of n-Alkyl Chains. 2. Long, All-Trans Chains. *J. Phys. Chem.* **1984**, *88*, 334–341.

(21) Himmelhaus, M.; Eisert, F.; Buck, M.; Grunze, M. Self-Assembly of *n*-Alkanethiol Monolayers. A Study by IR-Visible Sum Frequency Spectroscopy (SFG). *J. Phys. Chem. B* **2000**, *104*, 576–584.

(22) Arnold, R.; Terfort, A.; Wöll, C. Determination of Molecular Orientation in Self-Assembled Monolayers Using IR Absorption Intensities: The Importance of Grinding Effects. *Langmuir* **2001**, *17*, 4980–4989.

(23) Viana, A. S.; Jones, A. H.; Abrantes, L. M.; Kalaji, M. Redox Induced Orientational Changes in a Series of Short Chain Ferrocenyl Alkyl Thiols Self-Assembled on Gold(111) Electrodes. *J. Electroanal. Chem.* **2001**, *500*, 290–298.

(24) Viana, R. B.; da Silva, A. B. F.; Pimentel, A. S. Infrared Spectroscopy of Anionic, Cationic, and Zwitterionic Surfactants. *Adv. Phys. Chem.* **2012**, *2012*, 1–14.

(25) Ye, S.; Sato, Y.; Uosaki, K. Redox-Induced Orientation Change of a Self-Assembled Monolayer of 11-Ferrocenyl-1-Undecanethiol on a Gold Electrode Studied by *in Situ* FT-IRRAS. *Langmuir* **1997**, *13*, 3157–3161.

(26) Ye, S.; Haba, T.; Sato, Y.; Shimazu, K.; Uosaki, K. Coverage Dependent Behavior of Redox Reaction Induced Structure Change and Mass Transport at an 11-Ferrocenyl-1-

Undecanethiol Self-Assembled Monolayer on a Gold Electrode Studied by an *insitu* IRRAS–EQCM Combined System. *Phys. Chem. Chem. Phys.* **1999**, *1*, 3653–3659.

(27) Rudnev, A. V.; Zhumaev, U.; Utsunomiya, T.; Fan, C.; Yokota, Y.; Fukui, K. I.; Wandlowski, T. Ferrocene-Terminated Alkanethiol Self-Assembled Monolayers: An Electrochemical and in Situ Surface-Enhanced Infra-Red Absorption Spectroscopy Study. *Electrochim. Acta* **2013**, *107*, 33–44.

(28) Chidsey, C. E. D. Free Energy and Temperature Dependence of Electron Transfer at the Metal-Electrolyte Interface. *Science* **1991**, *4996*, 919–922.

(29) Chidsey, C. E. D.; Bertozzi, C. R.; Putvinski, T. M.; Mujsce, A. M. Coadsorption of Ferrocene-Terminated and Unsubstituted Alkanethiols on Gold: Electroactive Self-Assembled Monolayers. *J. Am. Chem. Soc.* **1990**, *112*, 4301–4306.

(30) Jangid, V.; Brunel, D.; Sanchez-Adaime, E.; Bharwal, A. K.; Dumur, F.; Duché, D.; Abel, M.; Koudia, M.; Buffeteau, T.; Nijhuis, C. A.; et al. Improving Orientation, Packing Density, and Molecular Arrangement in Self-Assembled Monolayers of Bianchoring Ferrocene-Triazole Derivatives by “Click” Chemistry. *Langmuir* **2022**, *38*, 3585–3596.

(31) Nerngchamnong, N.; Thompson, D.; Cao, L.; Yuan, L.; Jiang, L.; Roemer, M.; Nijhuis, C. A. Nonideal Electrochemical Behavior of Ferrocenyl-Alkanethiolate SAMs Maps the Microenvironment of the Redox Unit. *J. Phys. Chem. C* **2015**, *119*, 21978–21991.

(32) Patel, D. A.; Weller, A. M.; Chevalier, R. B.; Karos, C. A.; Landis, E. C. Ordering and Defects in Self-Assembled Monolayers on Nanoporous Gold. *Appl. Surf. Sci.* **2016**, *387*, 503–512.

(33) Kobayashi, Y.; Yokota, Y.; Wong, R. A.; Hong, M.; Takeya, J.; Osawa, S.; Ishiwari, F.; Shoji, Y.; Harimoto, T.; Sugimoto, K.; et al. Single-Molecule Observation of Redox Reactions Enabled by Rigid and Isolated Tripodal Molecules. *J. Phys. Chem. C* **2023**, *127*, 127–746.

(34) Zhang, S.; Lyu, X.; Hurtado Torres, C.; Darwish, N.; Ciampi, S. Non-Ideal Cyclic Voltammetry of Redox Monolayers on Silicon Electrodes: Peak Splitting Is Caused by Heterogeneous Photocurrents and Not by Molecular Disorder. *Langmuir* **2022**, *38*, 743–750.

(35) Patel, D. A.; Chevalier, R. B.; Weller, A. M.; Shakespeare, C. C.; Soares, E. J.; Landis, E. C. Porosity Effects on the Ordering and Stability of Self-Assembled Monolayers on Nanoporous Gold. *J. Phys. Chem. C* **2020**, *124*, 26851–26863.

(36) Pensa, E.; Karpowicz, R.; Jabłoński, A.; Trzybiński, D.; Woźniak, K.; Šakić, D.; Vrček, V.; Long, N. J.; Albrecht, T.; Kowalski, K. Gold-Induced Desulfurization in a Bis(Ferrocenyl) Alkane Dithiol. *Organometallics* **2019**, *38*, 2227–2232.

(37) Wong, R. A.; Yokota, Y.; Wakisaka, M.; Inukai, J.; Kim, Y. Discerning the Redox-Dependent Electronic and Interfacial Structures in Electroactive Self-Assembled Monolayers. *J. Am. Chem. Soc.* **2018**, *140*, 13672–13679.

(38) Dionne, E. R.; Dip, C.; Toader, V.; Badia, A. Micromechanical Redox Actuation by Self-Assembled Monolayers of Ferrocenylalkanethiolates: Evens Push More Than Odds. *J. Am. Chem. Soc.* **2018**, *140*, 10063–10066.

(39) Qi, L.; Tian, H.; Shao, H.; Yu, H. Z. Host-Guest Interaction at Molecular Interfaces: Cucurbit[7]Uril as a Sensitive Probe of Structural Heterogeneity in Ferrocenyl Self-Assembled Monolayers on Gold. *J. Phys. Chem. C* **2018**, *122*, 15986–15995.

(40) Dionne, E. R.; Badia, A. Electroactive Self-Assembled Monolayers Detect Micelle Formation. *ACS Appl. Mater. Interfaces* **2017**, *9*, 5607–5621.

(41) Laviron, E. General Expression of the Linear Potential Sweep Voltammogram in the Case of Diffusionless Electrochemical Systems. *J. Electroanal. Chem. Interfacial Electrochem.* **1979**, *101*, 19–28.

(42) Auletta, T.; van Veggel, F. C. J. M.; Reinhoudt, D. N. Self-Assembled Monolayers on Gold of Ferrocene-Terminated Thiols and Hydroxyalkanethiols. *Langmuir* **2002**, *18*, 1288–1293.

(43) Smalley, J. F.; Finklea, H. O.; Chidsey, C. E. D.; Linford, M. R.; Creager, S. E.; Ferraris, J. P.; Chalfant, K.; Zawodzinsk, T.; Feldberg, S. W.; Newton, M. D. Heterogeneous Electron-Transfer Kinetics for Ruthenium and Ferrocene Redox Moieties through Alkanethiol Monolayers on Gold. *J. Am. Chem. Soc.* **2003**, *125*, 2004–2013.

(44) Bain, C. D.; Troughton, E. B.; Tao, Y. T.; Evall, J.; Whitesides, G. M.; Nuzzo, R. G. Formation of Monolayer Films by the Spontaneous Assembly of Organic Thiols from Solution onto Gold. *J. Am. Chem. Soc.* **1989**, *111*, 321–335.

(45) Duffin, T. J.; Nerngchamnong, N.; Thompson, D.; Nijhuis, C. A. Direct Measurement of the Local Field within Alkyl-Ferrocenyl-Alkanethiolate Monolayers: Importance of the Supramolecular and Electronic Structure on the Voltammetric Response and Potential Profile. *Electrochim. Acta* **2019**, *311*, 92–102.

(46) Tender, L.; Carter, M. T.; Murray, R. W. Cyclic Voltammetric Analysis of Ferrocene Alkanethiol Monolayer Electrode Kinetics Based on Marcus Theory. *Anal. Chem.* **1994**, *66*, 3173–3181.

(47) Chen, Y.; Li, C. W.; Kanan, M. W. Aqueous CO_2 Reduction at Very Low Overpotential on Oxide-Derived Au Nanoparticles. *J. Am. Chem. Soc.* **2012**, *134*, 19969–19972.

(48) Yoon, Y.; Yan, B.; Surendranath, Y. Suppressing Ion Transfer Enables Versatile Measurements of Electrochemical Surface Area for Intrinsic Activity Comparisons. *J. Am. Chem. Soc.* **2018**, *140*, 2397–2400.
